# Development and Validation of a Personalized Model With Transfer Learning for Acute Kidney Injury Risk Estimation Using Electronic Health Records

**DOI:** 10.1001/jamanetworkopen.2022.19776

**Published:** 2022-07-01

**Authors:** Kang Liu, Xiangzhou Zhang, Weiqi Chen, Alan S. L. Yu, John A. Kellum, Michael E. Matheny, Steven Q. Simpson, Yong Hu, Mei Liu

**Affiliations:** 1 Big Data Decision Institute, Jinan University, Guangzhou, Guangdong, China; 2 Division of Nephrology and Hypertension and the Jared Grantham Kidney Institute, School of Medicine, University of Kansas Medical Center, Kansas City; 3 Center for Critical Care Nephrology, Department of Critical Care Medicine, University of Pittsburgh School of Medicine, Pittsburgh, Pennsylvania; 4 Department of Biomedical Informatics, Vanderbilt University School of Medicine, Nashville, Tennessee; 5 Department of Medicine, Vanderbilt University School of Medicine, Nashville, Tennessee; 6 Department of Biostatistics, Vanderbilt University School of Medicine, Nashville, Tennessee; 7 Geriatrics Research Education and Clinical Care Center, Veterans Affairs Tennessee Valley Healthcare System, Nashville; 8 Division of Pulmonary, Critical Care, and Sleep Medicine, Department of Internal Medicine, University of Kansas Medical Center, Kansas City; 9 Division of Medical Informatics, Department of Internal Medicine, University of Kansas Medical Center, Kansas City

## Abstract

**Importance:**

Acute kidney injury (AKI) is a heterogeneous syndrome prevalent among hospitalized patients. Personalized risk estimation and risk factor identification may allow effective intervention and improved outcomes.

**Objective:**

To develop and validate personalized AKI risk estimation models using electronic health records (EHRs), examine whether personalized models were beneficial in comparison with global and subgroup models, and assess the heterogeneity of risk factors and their outcomes in different subpopulations.

**Design, Setting, and Participants:**

This diagnostic study analyzed EHR data from 1 tertiary care hospital and used machine learning and logistic regression to develop and validate global, subgroup, and personalized risk estimation models. Transfer learning was implemented to enhance the personalized model. Predictor outcomes across subpopulations were analyzed, and metaregression was used to explore predictor interactions. Adults who were hospitalized for 2 or more days from November 1, 2007, to December 31, 2016, were included in the analysis. Patients with moderate or severe kidney dysfunction at admission were excluded. Data were analyzed between August 28, 2019, and May 8, 2022.

**Exposures:**

Clinical and laboratory variables in the EHR.

**Main Outcomes and Measures:**

The main outcome was AKI of any severity, and AKI was defined using the Kidney Disease: Improving Global Outcomes serum creatinine criteria. Performance of the models was measured with area under the receiver operating characteristic curve (AUROC), area under the precision-recall curve, and calibration.

**Results:**

The study cohort comprised 76 957 inpatient encounters. Patients had a mean (SD) age of 55.5 (17.4) years and included 42 159 men (54.8%). The personalized model with transfer learning outperformed the global model for AKI estimation in terms of AUROC among general inpatients (0.78 [95% CI, 0.77-0.79] vs 0.76 [95% CI, 0.75-0.76]; *P* < .001) and across the high-risk subgroups (0.79 [95% CI, 0.78-0.80] vs 0.75 [95% CI, 0.74-0.77]; *P* < .001) and low-risk subgroups (0.74 [95% CI, 0.73-0.75] vs 0.71 [95% CI, 0.70-0.72]; *P* < .001). The AUROC improvement reached 0.13 for the high-risk subgroups, such as those undergoing liver transplant and cardiac surgery. Moreover, the personalized model with transfer learning performed better than or comparably with the best published models in well-studied AKI subgroups. Predictor outcomes varied significantly between patients, and interaction analysis uncovered modifiers of the predictor outcomes.

**Conclusions and Relevance:**

Results of this study demonstrated that a personalized modeling with transfer learning is an improved AKI risk estimation approach that can be used across diverse patient subgroups. Risk factor heterogeneity and interactions at the individual level highlighted the need for agile, personalized care.

## Key Points

**Question:**

Are personalized models more accurate than traditional models in estimating acute kidney injury across subpopulations of hospitalized patients?

**Findings:**

In this diagnostic study involving 76 957 inpatient encounters, a new personalized model with transfer learning framework yielded improved and more equitable acute kidney injury estimation as well as accounted for the heterogeneity of risk factors and their variations in different patient subgroups.

**Meaning:**

Findings of this study suggest the advancement of clinical risk estimation toward personalized estimation and highlight the need for agile, personalized patient care.

## Teaser

This diagnostic study assesses the approaches to estimating acute kidney injury risk in hospitalized patients and proposes a novel model that uses machine learning.

## Introduction

Acute kidney injury (AKI) is a life-threatening clinical syndrome that is characterized by rapid reduction in kidney function and has complex etiologies and pathogenesis. Prevalence of hospital-acquired AKI varies by patient population, affecting 7% to 18% of general inpatients and greater than 50% of patients in the intensive care unit.^1,2,3^ Complex risk factors and their interactions hinder physicians from forecasting AKI risk.

Artificial intelligence has made substantial progress in AKI risk estimation. However, risk estimation models are predominantly built with predefined study cohorts, which are also known as global models.^4,5,6^ Existing global models for AKI estimation have achieved an area under the receiver operating characteristic curve (AUROC) of 0.66 to 0.80 in internal validation studies and 0.65 to 0.71 in external validation studies.^4^ Several studies have estimated AKI risk in patients in the intensive care unit using structured and unstructured electronic health record (EHR) data.^7,8,9,10^ Tomašev et al^5^ proposed a state-of-the-art deep learning–based AKI estimation model using the EHR system from the US Department of Veterans Affairs, but this model may have inherent gender bias. Koyner et al^6^ developed and externally validated^11^ a gradient boosting machine model with an AUROC higher than 0.85 for estimating moderate-to-severe AKI. A previous study^12^ revealed variable performance of the gradient boosting machine for AKI estimation across 6 health systems. Despite substantial progress, none of these global models has been evaluated in diverse subpopulations.

Global models can capture knowledge that is generalizable to a population but may ignore information that is specific to an individual or a subpopulation.^13,14,15,16^ An alternative is subgroup modeling,^17,18,19,20,21,22,23^ which is stratifying a population into subgroups according to patient differences and then building models for each subgroup. These subgroups, however, are defined by preexisting knowledge. For highly heterogeneous diseases, such as AKI, for which the underlying mechanisms are not yet fully elucidated, exhaustive subgroup modeling is impossible.

Personalized modeling is a promising approach to precise and equitable risk estimation.^24,25,26,27,28,29^ It builds an estimation model on demand for an incoming patient from an individualized cohort of similar patients. The model is optimized for the index patient rather than the average patient in a heterogeneous population. However, the number of similar patients in a high-dimensional EHR system is often small, which can be a factor in severe overfitting. Moreover, there is no systematic investigation on the necessity and feasibility of personalized modeling for AKI risk estimation.

In this diagnostic study, we explored personalized modeling by addressing the diminishing sample challenge. The first objective was to develop and validate personalized AKI risk estimation models using EHRs. The second objective was to examine whether personalized models were effective compared with global and subgroup models. The third objective was to assess the heterogeneity of risk factors and their outcomes in different subpopulations.

## Methods

The data set used in this diagnostic study met the Health Insurance Portability and Accountability Act deidentification criteria, and thus the study was deemed to be non–human participant research by the University of Kansas Medical Center Institutional Review Board. Data acquisition was approved by the University of Kansas Medical Center Data Request Oversight Committee. We followed the Transparent Reporting of a Multivariable Prediction Model for Individual Prognosis or Diagnosis (TRIPOD) reporting guideline.

### Study Cohort and Data Extraction

We used a retrospective cohort from previous AKI studies^30,31,32,33^ and extracted deidentified data from EHRs at the University of Kansas Medical Center, a tertiary care hospital.^34,35^ All adults who were hospitalized at the University of Kansas Health System for 2 or more days from November 1, 2007, to December 31, 2016, were included (representing 179 370 inpatient encounters). Inpatient stays were the unit of analysis, and some patients had multiple admissions. We excluded those with (1) fewer than 2 serum creatinine measurements, or (2) moderate-to-severe kidney dysfunction at admission (ie, estimated glomerular filtration rate <60 mL/min/1.73 m^2^ using the Modification of Diet in Renal Disease equation,^36^ or serum creatinine level >1.3 mg/dL [to convert to micromoles per liter, multiply by 88.4] within 24 hours of admission).

We extracted 1892 structured EHR variables, including demographic characteristics, vital signs, medications, medical history, admission diagnoses, and laboratory tests (eTable 1 in the Supplement).^37^ Race and ethnicity data were indicated in the EHR and included the following categories: African American, Asian, White, and other (including American Indian or Alaskan Native, Native Hawaiian or Other Pacific Islander, 2 races, and unreported race).

The estimation point was 1 day before onset for patients with AKI and 1 day before the last serum creatinine measurement for patients without AKI. Data preprocessing, missing data handling, and patient characteristics are described in eAppendix 1 and eTable 2 in the Supplement.

### AKI Definition and Evaluation 

Acute kidney injury was defined using the Kidney Disease: Improving Global Outcomes serum creatinine criteria.^38^ Baseline serum creatinine level was the last measurement within 2 days before admission or the first measurement after admission. All serum creatinine measures during a hospital stay were examined on a rolling basis to ascertain the presence and onset of AKI.

The personalized model with transfer learning was developed and evaluated through 5-fold cross-validation (eFigures 1 and 2 and eTables 3-7 in the Supplement). We compared the performances of the global, subgroup, and personalized models for estimating AKI in general, high-risk, and low-risk inpatients. No data balancing approach was used. Benchmarking models (eAppendix 4 in the Supplement) included global model, global model with transfer learning, subgroup model, subgroup model with transfer learning, personalized model, and personalized model with transfer learning.

We also conducted a literature review to compare the personalized model with transfer learning against models in published studies. To assess the role of sample size in AKI estimation, we randomly sampled a percentage of the study cohort to build the global model and controlled the number of similar patients for training the personalized models at the same level. The global model with smaller sample sizes was repeated 10 times to obtain the mean performance. To evaluate the models across patient subgroups, we identified 20 high-risk subgroups in the cohort using admission diagnoses and 20 known subgroups from the literature. To analyze heterogeneity of patients in subgroups, we calculated absolute Pearson correlation coefficient among the top 50 important predictors in different subgroups. Higher correlations among important predictors in a subgroup indicated that patients in the subgroup were more homogeneous with respect to each other and more heterogeneous with respect to general patients. To compare the personalized model with transfer learning and the subgroup model for each test patient in a subgroup, we built the personalized model with transfer learning using 10% of the overall training samples (eTable 3 in the Supplement) who exhibited the highest similarity to the test patient.

Model performance was measured with AUROC, area under the precision-recall curve (AUPRC), and calibration, and these measures were compared using the DeLong test,^39^ *z* test, and bootstrapping (eAppendix 5 in the Supplement). Comparisons between the personalized model with transfer learning and existing models in previous studies were conducted with *z* tests. Predictor importance was estimated by calculating the coefficients in logistic regression, AUROC gain, and interclass score difference^40^ (eAppendix 6 in the Supplement).

### Personalized Model With Transfer Learning 

The personalized model with transfer learning contains 4 modules (eAppendix 2 in the Supplement): (1) similar sample matching, which identifies similar patients for a given target patient; (2) transfer learning, which transfers knowledge from the global model to initialize training of personalized models (eAppendix 3 in the Supplement); (3) personalized modeling, which continues learning from similar patients; and (4) similarity measure optimization, which optimizes similarity measures in similar sample matching.

To identify similar patients, we applied the k-nearest neighbor algorithm and calculated distances between patients using all 1892 structured EHR variables. Each variable in the distance calculation was weighted by the similarity measure optimization module, which iteratively optimized the weights according to performance of personalized models during training. To address the diminishing sample problem after similar sample matching, we leveraged transfer learning^41^ using logistic regression as the base learner, and initialized the coefficient of each variable in the personalized logistic regression with its corresponding coefficient from the global logistic regression.

### Statistical Analysis 

To understand risk factor interactions in personalized models, we performed metaregression using PyMARE (Tal Yarkoni, Taylor Salo, and Thomas Nichols). Each personalized model was treated as an independent study. Study-level effect size of each target variable was calculated using its coefficients from the personalized models of patients who had the variable recorded, and the remaining variables were treated as study-level covariates. Because personalized models are not independent from each other, we performed subgroup analysis to verify the risk factor interactions by controlling the moderator found by metaregression and compared the outcomes of target predictors in patients who were exposed vs those who were not exposed to the moderator according to the coefficients in the logistic regression model or odds ratios that were calculated from the raw data (eAppendix 7 in the Supplement).

Significance of result in metaregression was based on the 2-sided *P* value returned by PyMARE (eAppendixes 5 and 7 in the Supplement). Significance of the changes in predictor outcomes in 2 subgroup models were calculated with the *z* test. Data were analyzed between August 28, 2019, and May 8, 2022.

## Results

The study cohort comprised 76 957 inpatient encounters. Of these hospitalized patients, the mean (SD) age was 55.5 (17.4) years and 42 159 were male individuals (54.8%) and 34 798 were female individuals (45.2%) (eTable 2 in the Supplement), about whom we collected a total of 1892 variables. Acute kidney injury occurred in 7259 patients (9.4%).

### Risk Estimation in General Inpatients

**Figure 1.**
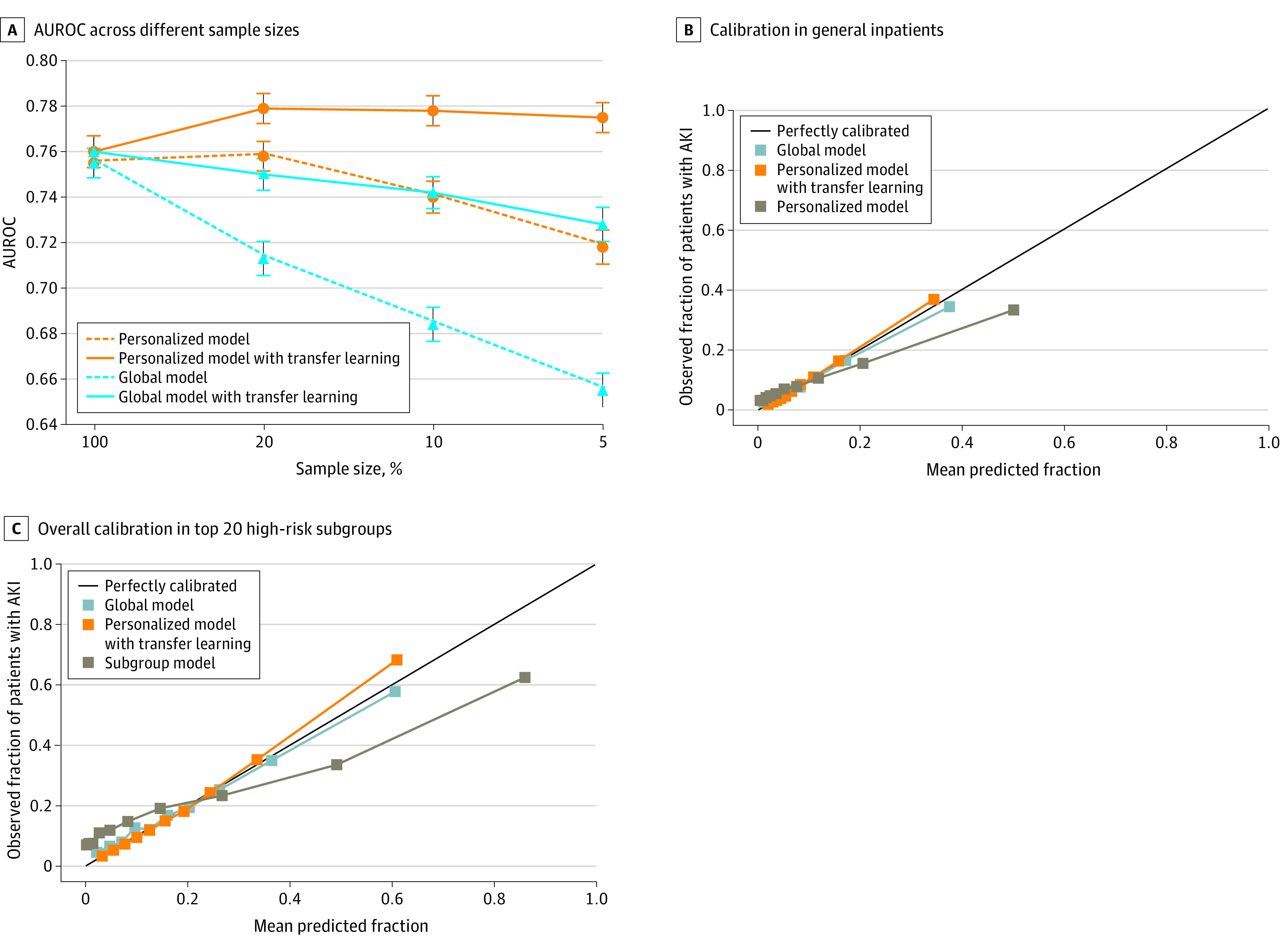
Comparison of Model Performance in General Inpatients In panels B and C, personalized models used 10% of overall training sample as the threshold for number of similar patients. Global model used 100% of training samples. AKI indicates acute kidney injury; AUROC, area under the receiver operating characteristic curve.

The personalized models outperformed the global models whenever the training sample size was less than 100% of the entire cohort (Figure 1). The personalized model with transfer learning, even at small sample sizes, outperformed the global model that was trained with a 100% sample size (0.78 [95% CI, 0.77-0.79] vs 0.76 [95% CI, 0.75-0.76]; *P* < .001). The remaining analyses used the personalized model with transfer learning that was built with a 10% sample size (eTable 3 in the Supplement). The AUROC for the personalized model with transfer learning that was trained with a 10% training sample size vs the global model that was trained with a 100% sample size was 0.78 (95% CI, 0.77-0.79) vs 0.76 (95% CI, 0.75-0.76; *P* < .001), and the AUPRC was 0.37 (95% CI, 0.36-0.39) vs 0.32 (95% CI, 0.31-0.33; *P* < .001), respectively (eFigure 3 in the Supplement). Calibration of the personalized model with transfer learning was nearly perfect and was better than the global model (0.0001 vs 0.0002; *P* = .13; personalized model with transfer learning performed better in 18 716 of 20 000 bootstrapping tests) (Figure 1B).

Transfer learning was associated with significantly mitigated deterioration of model estimations with smaller sample sizes (Figure 1A). The AUROC margins using 5% vs 100% sample size deteriorated only by 0.03 for the global model with transfer learning vs 0.10 for the global model. Transfer learning also was associated with improved calibration (0.0001 vs 0.0035; *P* < .001; personalized model with transfer learning performed better in all 20 000 bootstrapping tests) (Figure 1B). Nevertheless, the global model with transfer learning that used a smaller sample size still underperformed the global model with a 100% sample size, suggesting that the personalized model with transfer learning outperformed the global model primarily because of the personalized approach.

### Risk Estimation in High-Risk Admission Subgroups

**Figure 2.**
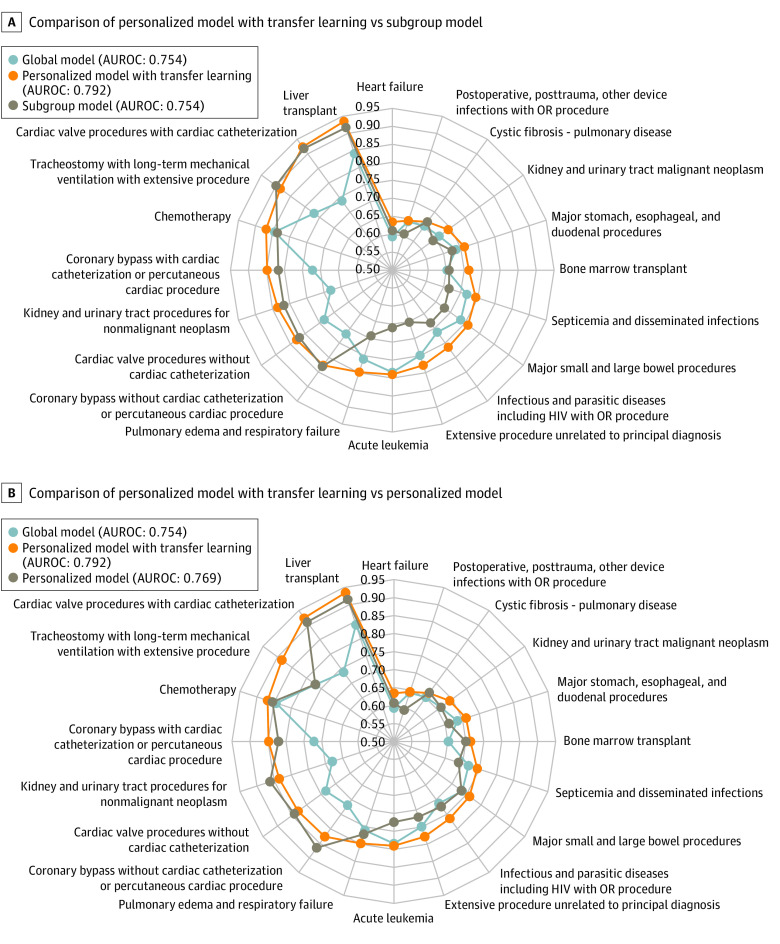
Radar Chart of the Area Under the Receiver Operating Characteristic Curve (AUROC) for Personalized and Subgroup Models Across 20 High-Risk Subgroups OR indicates operating room.

We compared global, subgroup, and personalized modeling in 20 subgroups stratified by admission diagnoses who had the highest AKI incidence (eTables 8 and 9 in the Supplement). The personalized model with transfer learning outperformed the global model (100% sample size) in all 20 subgroups, with a mean AUROC increase of 0.06 (95% CI, 0.005-0.17; *P* < .05 in 13 subgroups) (Figure 2A). Across the high-risk subgroups, the AUROC for the personalized model with transfer learning vs global model was 0.79 (95% CI, 0.78-0.80) vs 0.75 (95% CI, 0.74-0.77; *P* < .001), and the AUPRC was 0.58 (95% CI, 0.56-0.60) vs 0.48 (95% CI, 0.46-0.51; *P* < .001). Among patients with lower risk, the AUROC for the personalized model with transfer learning vs global model was 0.74 (95% CI, 0.73-0.75) vs 0.71 (95% CI, 0.70-0.72; *P* < .001), and the AUPRC was 0.22 (95% CI, 0.20-0.23) vs 0.20 (95% CI, 0.18-0.21; *P* < .001) (eFigure 4 in the Supplement).

The personalized model with transfer learning also outperformed the subgroup models in 17 of 20 subgroups (mean AUROC increase, 0.05; 95% CI, –0.01 to 0.128; *P* < .05 in 7 subgroups) (Figure 2A). Across the high-risk subgroups, AUROCs of the personalized model with transfer learning vs subgroup models were as follows: 0.79 (95% CI, 0.78-0.80) vs 0.75 (95% CI, 0.74-0.77; *P* < .001), and the AUPRCs were 0.58 (95% CI, 0.56-0.60) vs 0.52 (95% CI, 0.50-0.54; *P* < .001) (eFigure 4 in the Supplement). Overall calibration of the personalized model with transfer learning across the 20 subgroups was significantly better than that of the subgroup models (0.0006 vs 0.0113; *P* < .001; personalized model with transfer learning performed better in all 20 000 bootstrapping tests) (Figure 1C; eFigure 5 in the Supplement). The personalized models with transfer learning performed better than the subgroup models with transfer learning in 18 of 20 subgroups. Overall, the AUROC across the 20 subgroups was 0.79 (95% CI, 0.78-0.80) vs 0.78 (95% CI, 0.77-0.79; *P* < .001), and the AUPRC was 0.58 (95% CI, 0.56-0.60) vs 0.56 (95% CI, 0.54-0.58; *P* < .001) (eFigures 4 and 6, eTables 9 and 10 in the Supplement). We also evaluated the performance of the personalized model with transfer learning in retrieving patients with AKI and found that the personalized models can identify 11.97% to 20.95% more patients with AKI than the global models and 7.18% to 13.45% more than the subgroup models (eTables 11 and 12 in the Supplement).

The personalized model with transfer learning adapted well to subpopulations with different levels of heterogeneity. In subgroups of patients with cardiac surgery (mean [SD] Pearson correlation in 4 subgroups, 0.24 [0.27], 0.13 [0.21], 0.22 [0.25], and 0.25 [0.27]), liver transplant (mean [SD] Pearson correlation, 0.22 [0.20]), kidney and urinary tract procedures for non–malignant neoplasm (mean [SD] Pearson correlation, 0.10 [0.16]), and tracheostomy with long-term mechanical ventilation with extensive procedure (mean [SD] Pearson correlation, 0.15 [0.17]), the important predictors had greater correlation, suggesting that patients with AKI in these subgroups had a similar manifestation (eFigure 7 in the Supplement). It is hard for the global model to capture important predictors in patients in these subgroups because they are different from general patients, whereas both the personalized model with transfer learning (mean [SD] AUROC increase, 0.13 [0.03]) and the subgroup models performed well. However, the correlations between important predictors were lower in the subgroups with pulmonary edema and respiratory failure (mean [SD] Pearson correlation, 0.09 [0.15]), acute leukemia (mean [SD] Pearson correlation, 0.09 [0.15]), extensive procedure unrelated to principal diagnosis (mean [SD] Pearson correlation, 0.10 [0.15]), infectious and parasitic diseases (mean [SD] Pearson correlation, 0.09 [0.15]), septicemia and disseminated infections (mean [SD] Pearson correlation, 0.08 [0.16]), and major small and large bowel procedures (mean [SD] Pearson correlation, 0.09 [0.16]) (eFigure 7 in the Supplement). This finding indicates higher heterogeneity among patients in these subgroups. The personalized model with transfer learning can still capture the heterogeneity in these subgroups and outperformed the global model (0.77 [95% CI, 0.75-0.79] vs 0.74 [95% CI, 0.72-0.76]; *P* < .001), but in this scenario the subgroup models performed the worst (mean [SD] AUROC difference, 0.10 [0.02]).

### Risk Estimation in Known AKI Subgroups

The Table shows a comparison of the AUROC for the models in 20 well-studied AKI subgroups from the literature (eTable 13 in the Supplement).^17,20,22,42,43,44,45,46,47,48,49,50,51,52,53,54,55,56,57,58,59,60,61,62,63,64,65,66,67^ The personalized model with transfer learning was superior to each of the current models, significantly outperforming the global model in 16 subgroups, the subgroup model in 11 subgroups, and the subgroup model with transfer learning in 9 subgroups. For example, among patients older than 65 years, AUROC was 0.76 (95% CI, 0.74-0.77) for the personalized model with transfer learning, 0.73 (95% CI, 0.72-0.75; *P* < .001) for the global model, 0.71 (95% CI, 0.70-0.72; *P* < .001) for the subgroup model, and 0.73 (95% CI, 0.72-0.75; *P* < .001) for the subgroup model with transfer learning. The AUPRC comparison is shown in eTable 14 in the Supplement. Among patients older than 65 years, AUPRC was 0.37 (95% CI, 0.35-0.39) for the personalized model with transfer learning, 0.31 (95% CI, 0.28-0.33; *P* < .001) for the global model, 0.28 (95% CI, 0.26-0.30; *P* < .001) for the subgroup model, and 0.32 (95% CI, 0.30-0.35; *P* < .001) for the subgroup model with transfer learning.

**Table.** Comparison of the Personalized Model With Transfer Learning With Models in Selected Published Studies in 20 Subgroups^a^

Subgroup of patients	Current study					Previous studies			
	Sample size (AKI incidence), No.	Personalized model with transfer learning AUROC (95% CI)	Global model AUROC (95% CI)	Subgroup model AUROC (95% CI)	Subgroup model with transfer learning AUROC (95% CI)	Study location	Sample size	AUROC	Source
Liver transplant	240 (121)	0.94 (0.90-0.97)	0.84 (0.78-0.90)^b^	0.92 (0.88-0.95)	0.91 (0.88-0.95)	Korea	1211	0.61-0.90	Lee et al,^17^ 2018
						Korea	538	0.85-0.86	Park et al,^42^ 2015^b^
PCI	1496 (113)	0.76 (0.71-0.81)	0.72 (0.66-0.77)^b^	0.69 (0.63-0.74)^b^	0.71 (0.65-0.76)^b^	Canada	7888	0.65-0.76	Ma et al,^43^ 2019
						United States	947 012	0.71	Tsai et al,^22^ 2014^b^
						United States	1 917 960	0.72-0.79	Huang et al,^44^ 2018
						United States	3 038 537	0.78-0.79	Huang et al,^45^ 2019
PCI or cardiac catheterization	2487 (179)	0.78 (0.74-0.81)	0.74 (0.70-0.78)^b^	0.75 (0.72-0.79)	0.77 (0.73-0.81)	United States	1507	0.73-0.74	Blanco et al,^46^ 2021
PCI and AMI	539 (40)	0.78 (0.69-0.86)	0.71 (0.62-0.80)^b^	0.71 (0.61-0.81)	0.70 (0.61-0.80)^b^	United States	1144	0.76	Zambetti et al,^47^ 2017
						United States	148 797	0.74	Tsai et al,^22^ 2014
PCI and non-AMI	957 (73)	0.76 (0.70-0.81)	0.72 (0.66-0.79)	0.66 (0.59-0.74)^b^	0.69 (0.62-0.76)^b^	United States	274 854	0.70	Tsai et al,^22^ 2014^b^
AMI	692 (52)	0.76 (0.69-0.83)	0.71 (0.63-0.78)^b^	0.68 (0.60-0.77)^b^	0.72 (0.64-0.80)	China	1495	0.68-0.82	Sun et al,^48^ 2020
						China	6014	0.73-0.81	Xu et al,^49^ 2019
CABG	962 (264)	0.84 (0.81-0.87)	0.72 (0.68-0.76)^b^	0.83 (0.80-0.87)	0.84 (0.81-0.87)	China	1748	0.74	Lin et al,^50^ 2020
						Turkey	193	0.5-0.84	Gursoy et al,^51^ 2015
CABG or valve surgery	1657 (440)	0.85 (0.82-0.87)	0.73 (0.71-0.76)^b^	0.86 (0.84-0.88)^b^	0.85 (0.83-0.87)	Brazil	818	0.85	Palomba et al,^52^ 2007
						China	8385	0.74-0.82	Jiang et al,^20^ 2016
CABG or valve surgery recipient and older age									
Age, y									
>65	673 (221)	0.82 (0.78-0.85)	0.68 (0.63-0.72)^b^	0.84 (0.81-0.87)	0.81 (0.77-0.84)	China	848	0.63-0.80	Hu et al,^53^ 2021
56-65	580 (140)	0.85 (0.81-0.89)	0.78 (0.73-0.82)^b^	0.82 (0.78-0.87)	0.78 (0.74-0.83)^b^				
Cardiac surgery	1803 (466)	0.84 (0.82-0.86)	0.73 (0.70-0.76)^b^	0.85 (0.82-0.87)	0.85 (0.82-0.87)	Australia and New Zealand	22 731	0.67-0.72	Coulson et al,^54^ 2021
						Norway	5029	0.82	Berg et al,^19^ 2013
						United Kingdom	30 854	0.68-0.74	Birnie et al,^55^ 2014^b^
TKA	1398 (57)	0.71 (0.63-0.79)	0.71 (0.64-0.79)	0.53 (0.43-0.62)^b^	0.63 (0.55-0.71)^b^	Korea	5757	0.78-0.89	Ko et al,^56^ 2022
Orthopedic surgery	5659 (332)	0.77 (0.75-0.79)	0.75 (0.73-0.78)	0.73 (0.71-0.76)^b^	0.77 (0.74-0.79)	United Kingdom	10 615	0.70-0.73	Bell et al,^57^ 2015^b^
GI surgery	2144 (291)	0.75 (0.72-0.78)	0.72 (0.69-0.75)^b^	0.69 (0.66-0.73)^b^	0.75 (0.71-0.78)	United Kingdom and Ireland	4544	0.65	Patel et al,^58^ 2019^b^
GI cancers	196 (15)	0.83 (0.71-0.94)	0.73 (0.60-0.86)^b^	0.65 (0.50-0.80)	0.71 (0.53-0.89)	China	6495	0.7-0.79	Li et al,^59^ 2020
Hematologic malignant neoplasm	1324 (215)	0.76 (0.73-0.80)	0.74 (0.70-0.77)^b^	0.69 (0.65-0.73)^b^	0.74 (0.70-0.77)^b^	China	2395	0.76-0.81	Li et al,^60^ 2020^b^
Cisplatin	222 (21)	0.66 (0.52-0.80)	0.71 (0.60-0.82)	0.59 (0.45-0.73)	0.63 (0.48-0.77)	United States	4481	0.70	Motwani et al,^61^ 2018
Vancomycin	13 287 (1920)	0.76 (0.74-0.77)	0.73 (0.71-0.74)^b^	0.71 (0.70-0.73)^b^	0.74 (0.73-0.75)^b^	China	524	0.79	Xu et al,^62^ 2020
Vancomycin user and older age									
Age, y									
>65	3814 (521)	0.75 (0.73-0.77)	0.72 (0.70-0.74)^b^	0.67 (0.65-0.70)^b^	0.73 (0.70-0.75)^b^	China	255	0.74	Pan et al,^63^ 2020
56-65	3436 (499)	0.76 (0.73-0.78)	0.73 (0.70-0.75)^b^	0.66 (0.63-0.69)^b^	0.73 (0.70-0.76)^b^				
Sepsis	2306 (349)	0.74 (0.72-0.77)	0.72 (0.69-0.75)^b^	0.67 (0.64-0.70)^b^	0.73 (0.70-0.75)	United States	2917	0.79	Deng et al,^64^ 2020^b^
						United States	15 726	0.71	Fan et al,^65^ 2021^b^
Older age									
Age, y									
>65	16 609 (1759)	0.76 (0.74-0.77)	0.73 (0.72-0.75)^b^	0.71 (0.70-0.72)^b^	0.73 (0.72-0.75)^b^	United States	25 521	0.66-0.66	Kate et al,^66^ 2016^b^ and Kate et al,^67^ 2020^b^
56-65	14 437 (1537)	0.77 (0.76-0.78)	0.75 (0.74-0.77)^b^	0.72 (0.71-0.74)^b^	0.75 (0.74-0.76)^b^	United States	44 691	0.57-0.72	

Abbreviations: AKI, acute kidney injury; AMI, acute myocardial infarction; AUROC, area under the receiver operating characteristic curve; CABG, coronary artery bypass graft; GI, gastrointestinal; PCI, percutaneous coronary intervention; TKA, total knee arthroplasty.

^a^ For studies that used multiple modeling approaches, we reported only the AUROC of the logistic regression model and the best model.

^b^ *P* < .05 compared with personalized model with transfer learning.

In addition, the personalized model with transfer learning compared favorably with 30 previously published subgroup models. Among 32 comparisons, the personalized model with transfer learning had superior performance vs the published models in 9 comparisons and worse in only 2 comparisons. For example, in the subgroup with percutaneous coronary intervention and nonacute myocardial infarction, the AUROC was 0.76 (95% CI, 0.70-0.81) for the personalized model with transfer learning, whereas the reported AUROC of the previous model built with a sample size that was 280 times greater than the present sample size was 0.70 (95% CI, 0.69-0.71; *P* = .04). In the subgroup with hematologic malignant neoplasm, the personalized model with transfer learning performed as well as the logistic regression model (0.76 vs 0.76) in Li et al^60^ but was worse than their bayesian network model (0.76 [95% CI, 0.73-0.80] vs 0.81 [95% CI, 0.79-0.84]). For the sepsis subgroup, both previous studies used the MIMIC III (Medical Information Mart for Intensive Care) data set, and the model in the present study was significantly better than one of the logistic regression models^65^ (0.74 [95% CI, 0.72-0.77] vs 0.71 [95% CI, 0.70-0.73]) but worse than the other^64^ (0.79; 95% CI, 0.76-0.82).

The published studies incorporated specific factors (eg, details on surgery and nephrotoxin exposure) and estimated glomerular filtration rate or serum creatinine level (kidney function indicators that we avoided) for the subgroups and had larger sample sizes. When the subgroup model with transfer learning and the subgroup model were retrained for the 2 subgroups on the present data, the personalized model with transfer learning outperformed the subgroup model with a mean AUROC increase of 0.08 (0.76 [95% CI, 0.73-0.80] vs 0.69 [95% CI, 0.65-0.73]; *P* < .001 in the subgroup with hematologic malignant neoplasm and 0.74 [95% CI, 0.72-0.77] vs 0.67 [95% CI, 0.64-0.70]; *P* < .001 in the subgroup with sepsis) and outperformed the subgroup model with transfer learning with a mean AUROC increase of 0.02 (0.76 [95% CI, 0.73-0.8] vs 0.74 [95% CI, 0.70-0.77]; *P* = .007 in the subgroup with hematologic malignant neoplasm; 0.74 [95% CI 0.72-0.77] vs 0.73 [0.70-0.75; *P* = .054] in the subgroup with sepsis).

### Heterogeneity of Predictor Outcome Across Subgroups

**Figure 3.**
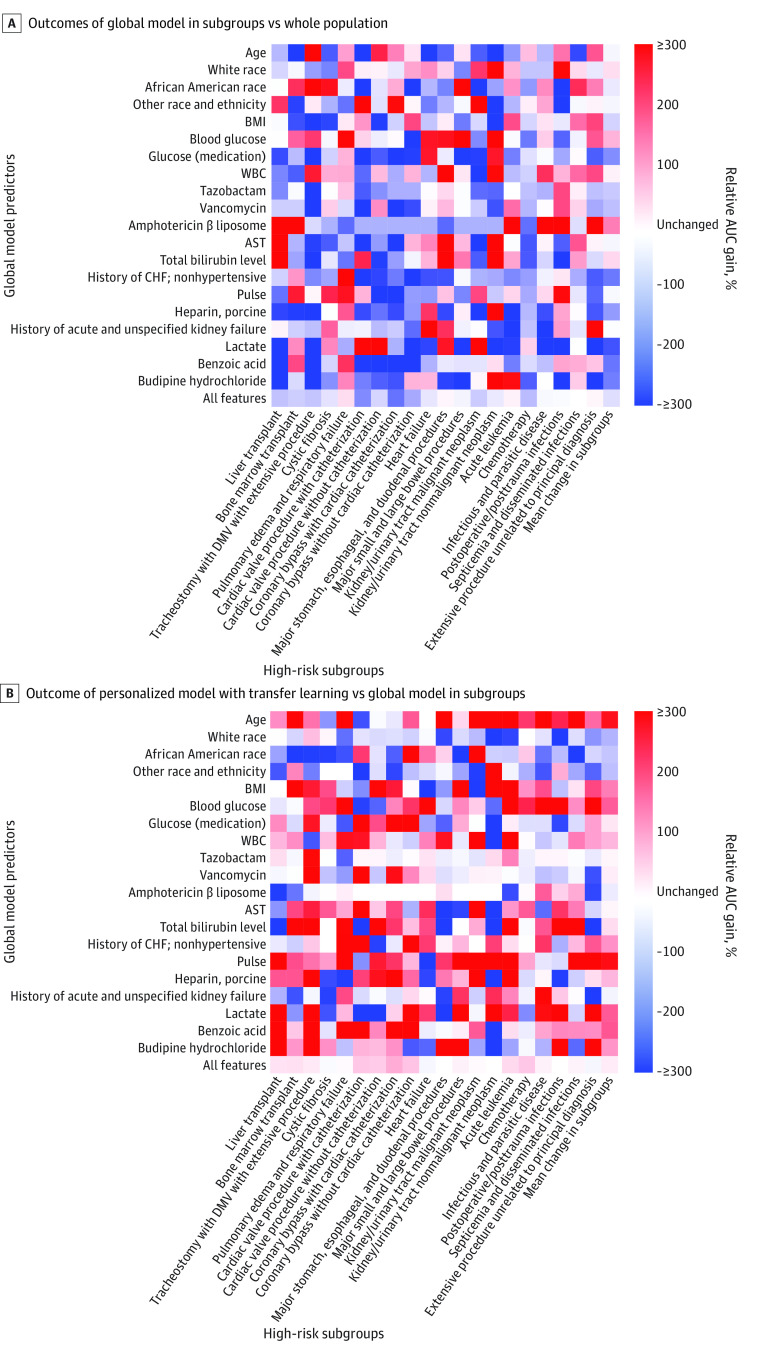
Heatmaps of Outcomes of Top 20 Global Model Predictors Across 20 High-Risk Subgroups A, Relative effect was calculated as follows: (area under the curve [AUC] gain of predictor when global model was used in subgroup − AUC gain of predictor when global model was used in whole population) / (AUC gain of predictor when global model was used in whole population). Red represents increased and blue represents decreased predictive effect in subgroups vs whole population. B, Relative effect was calculated as follows: (AUC gain of predictor when personalized model with transfer learning was used in subgroup − AUC gain of predictor when global model was used in subgroup) / (AUC gain of predictor when global model was used in general patients). Red represents increased and blue represents decreased predictive effect in personalized model with transfer learning vs global model. Other race and ethnicity included American Indian or Alaskan Native, Native Hawaiian or Other Pacific Islander, 2 races, and unreported race. AST indicates aspartate aminotransferase; BMI, body mass index; CHF, congestive heart failure; DMV, durative mechanical ventilation; WBC, white blood cell.

Figure 3A shows the outcome of each of the top 20 predictors in the global model when applied to the 20 high-risk subgroups compared with when they were applied to the entire population. The outcome of the predictors decreased in 240 of 400 predictor-subgroup combinations, with a mean decrease of 47% across all combinations; the distribution of several predictors that had large outcome change was similar between general patients and patients in subgroups (eFigures 8-10 in the Supplement). The AUROC of the global model across the 20 high-risk sugbroups based on the top 20 predictors was only 0.61 (95% CI, 0.60-0.63). By contrast, when these same predictors were applied to the same subgroups with the personalized model with transfer learning (compared with the global model), the predictors were associated with more benefits in most combinations (Figure 3B), and the AUROC was 0.65 (95% CI, 0.63-0.66; *P* < .001). This finding suggests that one reason the personalized model with transfer learning outperformed the global model in different subgroups was that the outcome of many factors that appeared highly predictive in the population as a whole changed when applied to certain subgroups.

**Figure 4.**
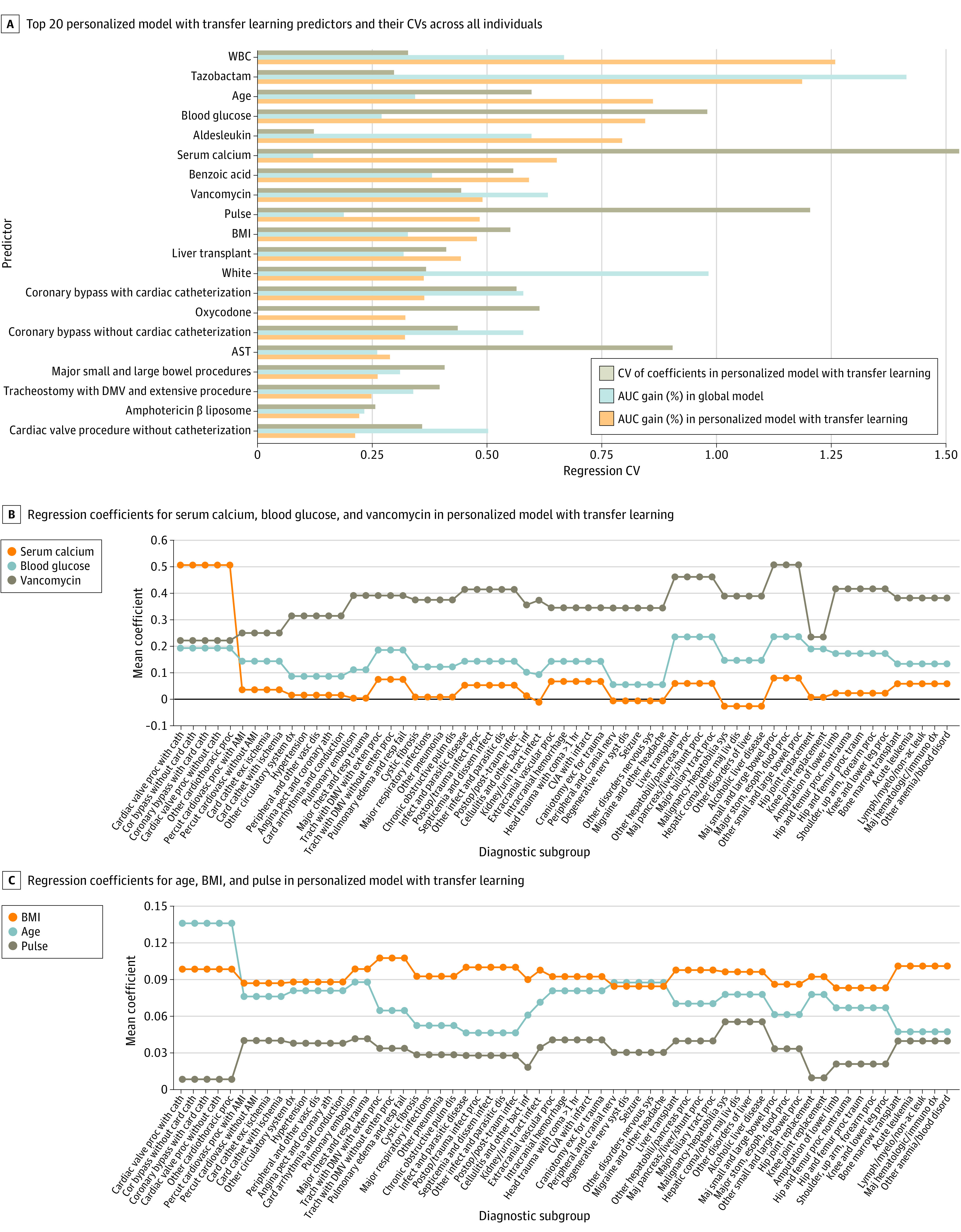
Outcomes of Top 20 Personalized Model With Transfer Learning Predictors Across 15 Large Diagnostic Subgroups To improve the stability of results, the 63 subgroups in Figure 4B and C were further abstracted into 15 large subgroups, both full name of the 63 diagnostic subgroups and their classification are provided in eTable 15 in the Supplement. Predictors outcome in each of the 63 subgroups are presented in eFigures 16 and 17 in the Supplement. AMI indicates acute myocardial infarction; AST, aspartate aminotransferase; AUC, area under the curve; BMI, body mass index; DMV, durative mechanical ventilation; WBC, white blood cell.

The outcomes of the top predictors for general patients estimated by the personalized model with transfer learning differed from those estimated by the global model (Figure 4A; eFigure 11 in the Supplement). The coefficient of variation of the regression coefficients for these features was high (mean of 59.4%) (Figure 4A), with similar results among the top 200 predictors (eFigure 12 in the Supplement), indicating that the varying outcome of these features across different types of patients was being modeled in the personalized model with transfer learning. This finding is illustrated in Figure 4B, which shows how the coefficients changed across subgroups for 6 well-known predictors with high interindividual variability. For example, the coefficients for age (0.14; 182.41% of mean coefficient among the 63 small subgroups) and serum calcium (0.51; 760.89% of mean coefficient among the 63 small subgroups) were the highest in the cardiac surgery subgroup, whereas the coefficients for pulse (0.008; 25.21% of mean coefficient among the 63 small subgroups) and vancomycin (0.22; 61.22% of mean coefficient among the 63 small subgroups) were the lowest in this subgroup. Predictor outcome estimates between personalized and subgroup models are provided in eFigures 13-17 in the Supplement.

### Predictor Interactions

The personalized model can facilitate risk factor interaction analysis. Patterns of changes in predictor outcome in subgroups were similar between the personalized model with transfer learning and the subgroup models, and conflicting results were mostly not significant (eFigures 15-17, eTables 16 and 17 in the Supplement). We explored interactions between medications and diagnoses within the personalized model with transfer learning (eAppendix 7, eTables 16 and 17 in the Supplement). Some interactions identified were supported by previous evidence, such as an important outcome of serum calcium level specific to patients who had cardiac surgery, mechanical ventilation, and burns^68,69,70,71,72,73,74,75,76^ or an interaction of both gastrointestinal surgery and tazobactam with vancomycin.^77,78^

Unknown interactions were also identified. For example, the association between serum calcium level and AKI varied with aldesleukin exposure. Among patients using aldesleukin, AKI incidence for those with normal serum calcium level was 93%, but it was only 32% in patients with abnormal serum calcium level.

Other hypothesized interactions were refuted. For example, previous studies suggested that infection-induced AKI was more common in older patients.^79,80^ However, when we examined patients who were admitted for systemic infection, the association of age with AKI risk decreased significantly. For example, for the subgroup admitted with septicemia and disseminated infections, AKI incidence was 13.4% (103 of 771) in patients older than 65 years, compared with 16.6% (150 of 906) in patients younger than 45 years. Similar results were obtained when analyzing patients who were exposed to antibiotics (Figure 4B; eTables 16 and 17 in the Supplement).

## Discussion

Clinical risk estimation models are commonly trained as global models. This study found that global models were significantly associated with patient heterogeneity and did not work equitably well across subpopulations. The most important predictors for the whole population that can be identified by a global model can be completely different from the predictors that are important within subpopulations; thus, estimations using a global model for patients with heterogeneous conditions cannot be trusted.

We developed the personalized model with transfer learning to improve AKI risk estimation in hospitalized patients. Results of the analyses showed that the personalized model with transfer learning outperformed the global model across general, high-risk, and low-risk subgroups (eFigure 4 in the Supplement). The personalized model with transfer learning also outperformed traditional subgroup models in most high-risk subgroups and performed better than or comparably to the state-of-the-art expert-driven subgroup models in the literature. In-depth analyses revealed that personalized modeling can dynamically adapt to subpopulations with varying levels of heterogeneity and sample sizes and can enable a deeper understanding of risk factor outcomes. Moreover, we found that transfer learning addressed the diminishing sample challenge and was associated with significantly improved performance for both the personalized and subgroup models. Because the transfer learning technique we developed preserves model interpretability, the approach has high translational potential.

Multiple factors and their interactions can affect AKI risk. This study uncovered significant variation in predictor outcomes across patients, and after considering this variation in the personalized models, the importance of predictors changed significantly compared with their importance in the global model. Using metaregression, we identified many significant predictor interactions. Some of these have been previously reported, but novel interactions were also identified.

We believe this study has substantial implications. First, it reached a milestone in clinical risk estimation because it advanced general estimation toward personalized estimation. Integration of transfer learning with personalized modeling eliminated the algorithm dependency on large training data. Second, because personalized models are dynamically built for an incoming patient, it is not necessary to build static models with the same target for various subpopulations as was done in previous research. Third, the present study found that the variation in AKI predictor outcomes in the personalized models was highly correlated with modifiable factors (Figure 3 and Figure 4; eAppendix 7, eFigure 11, eTables 16-18 in the Supplement). Thus, physicians may need to continuously monitor the changing outcomes of risk factors and adjust the interventions accordingly. The proposed algorithm can run in the background to notify physicians of impending AKI and the risk factors that need modification to avert AKI. It is critical to transform patient care from one that relies on common clinical pathways to one that is agile, personalized, and powered by artificial intelligence.

### Limitations

This study has several limitations. First, we did not use all routinely available EHR variables (ie, all laboratory results). Their inclusion may improve estimation but should not change the overall conclusions. Second, validation of the personalized model with transfer learning was limited to a single hospital. Third, a patient similarity measure was assumed to work uniformly well across subpopulations. Fourth, we chose logistic regression as the base learner because of its interpretability and common use in clinical research. Advanced methods, such as deep learning, could improve performance, albeit at the expense of interpretability. Fifth, we performed metaregression on limited predictors only to assess the interaction effect, but much more can be learned from such analyses. Sixth, we limited the analysis to patients with normal baseline kidney function to avoid confounding acute and chronic kidney disease. Seventh, we limited risk estimation for AKI to the next 24 hours. Previous work showed that important predictors can be different between time windows.^40,81^ Further research is required to ascertain the best approach to matching similar samples with time-series data.

## Conclusions

In this diagnostic study, we found that personalized modeling is an improved approach to AKI risk estimation across diverse patient subgroups. It advanced clinical risk estimation toward personalized estimation. The findings on risk factor heterogeneity and interactions at the individual level reinforced the need to transition to agile, personalized care.

## Notes

Supplement. **eAppendix 1.** Data Extraction **eAppendix 2.** Personalized Model With Transfer Learning (PMTL) **eAppendix 3.** Mechanism of Transfer Learning **eAppendix 4.** Detail of Benchmarking Models **eAppendix 5.** Statistical Analysis **eAppendix 6.** Predictor Importance Estimation **eAppendix 7.** Interaction Analysis for Important Predictors in PMTL **eFigure 1.** Validation Scheme of This Study **eFigure 2.** AUPRC Comparison of Personalized Models With and Without Similarity Measure Learning in General Patients Based on 4 of 5 Data Folds Used for Testing **eFigure 3.** Model Discrimination Comparison in General Inpatients **eFigure 4.** Overall Performance of Personalized and Subgroups Modeling In and Out of Top 20 High-Risk Subgroup **eFigure 5.** Calibration of Global, Subgroup and Personalized Model in Each of Top 20 High-Risk Subgroup **eFigure 6.** Radar Chart of AUROC Comparison for Subgroup Models With Transfer Learning **eFigure 7.** Absolute Pearson Correlation Coefficient Among Top-50 Important Predictors in Different Subgroups **eFigure 8.** Average Value Changes of Top-20 Predictors Determined by Global Model in Subgroups **eFigure 9.** Standard Deviation Changes of Top-20 Predictors Determined by Global Model in Subgroups **eFigure 10.** Wasserstein Distance of Top-20 Predictor Distribution Between Patients in General and Subgroups **eFigure 11.** Top 20 Predictors Where Their Effects Differed Between Global Model and PMTL **eFigure 12.** Coefficient of Variation of the Regression Coefficients of Top-200 Features in PMTL **eFigure 13.** Difference of Feature Effects Between PMTL and Other Models: A Case Study of Subgroup of Cardiac Valve Procedure With Cardiac Catheterization **eFigure 14.** Difference of Feature Effects Between PMTL and Other Models: A Case Study of Subgroup of Infect & Parasitic Disease **eFigure 15.** Effect Change of Factors Estimated by Subgroup Models and Meta-Regression on PMTL **eFigure 16.** Comparison of Coefficients Estimated by PMTL and Subgroup Models, Cases of Age, Serum Calcium and Blood Glucose **eFigure 17.** Comparison of Coefficients Estimated by PMTL and Subgroup Models, Cases of BMI, Pulse and Vancomycin **eTable 1.** Variables Used in This Study **eTable 2.** Characteristics of Patients **eTable 3.** AUROC of Different Models in Validation Set **eTable 4.** AUROC of Personalized Model Among Similar Samples in Validation Set **eTable 5.** Performance of Similarity Measure Learning in Test Set **eTable 6.** Performance of Sample Weighting in Test Set **eTable 7.** Performance (AUROC) of Feature Selection in Dealing With Overfitting in Personalized Modeling **eTable 8.** List of APR-DRG Selected as High-Risk Subgroups **eTable 9.** AUROC of Models in 31 High Risk DRG Subgroups With At Least 20 AKI Patients **eTable 10.** AUPRC of Models in 31 High Risk DRG Subgroups With At Least 20 AKI Patients **eTable 11.** Superiority of PMTL in Recalling AKI Patients in All Top 20 High-Risk Subgroups **eTable 12.** Superiority of PMTL in Recalling AKI Patients in Each of Top 20 High-Risk Subgroups **eTable 13.** Details About 55 AKI Prediction Researches for Specific Subgroup Patients Can Be Identified by Our Data **eTable 14.** AUPRC Comparison of Models in 20 Well-Studied Subgroups **eTable 15.** List of APR-DRG Presented in Fig 4 of Main Text **eTable 16.** Significant Interactions Between 6 Important Predictors and Disease in Meta-Regression and Their Verification **eTable 17.** Significant Interactions Between 6 Important Predictors and Disease in Meta-Regression (Top-5 Mainly) and Their Verification **eTable 18.** Top 50 Features in Patient Similarity Calculation
eReferences
