# Errors in the Supplement

**DOI:** 10.1001/jamanetworkopen.2022.32183

**Published:** 2022-08-30

In the Original Investigation titled “Development and Validation of a Personalized Model With Transfer Learning for Acute Kidney Injury Risk Estimation Using Electronic Health Records,”^1^ published on July 1, 2022, the formula of expected calibration error was omitted in eAppendix 5, and expected calibration error was incorrectly described as Brier score. The Supplement has been corrected.
