# Supplementary material for: Development and Validation of a Personalized Model With Transfer Learning for Acute Kidney Injury Risk Estimation Using Electronic Health Records
Source: JAMA Netw Open. 2022 Jul 1;5(7):e2219776. doi: 10.1001/jamanetworkopen.2022.19776 (PMC9250052; doi:10.1001/jamanetworkopen.2022.19776)
Supplement: Supplement. — eAppendix 1. Data Extraction eAppendix 2. Personalized Model With Transfer Learning (PMTL) eAppendix 3. Mechanism of Transfer Learning eAppendix 4. Detail of Benchmarking Models eAppendix 5. Statistical Analysis eAppendix 6. Predictor Importance Estimation eAppendix 7. Interaction Analysis for Important Predictors in PMTL eFigure 1. Validation Scheme of This Study eFigure 2. AUPRC Comparison of Personalized Models With and Without Similarity Measure Learning in General Patients Based on 4 of 5 Data Folds Used for Testing eFigure 3. Model Discrimination Comparison in General Inpatients eFigure 4. Overall Performance of Personalized and Subgroups Modeling In and Out of Top 20 High-Risk Subgroup eFigure 5. Calibration of Global, Subgroup and Personalized Model in Each of Top 20 High-Risk Subgroup eFigure 6. Radar Chart of AUROC Comparison for Subgroup Models With Transfer Learning eFigure 7. Absolute Pearson Correlation Coefficient Among Top-50 Important Predictors in Different Subgroups eFigure 8. Average Value Changes of Top-20 Predictors Determined by Global Model in Subgroups eFigure 9. Standard Deviation Changes of Top-20 Predictors Determined by Global Model in Subgroups eFigure 10. Wasserstein Distance of Top-20 Predictor Distribution Between Patients in General and Subgroups eFigure 11. Top 20 Predictors Where Their Effects Differed Between Global Model and PMTL eFigure 12. Coefficient of Variation of the Regression Coefficients of Top-200 Features in PMTL eFigure 13. Difference of Feature Effects Between PMTL and Other Models: A Case Study of Subgroup of Cardiac Valve Procedure With Cardiac Catheterization eFigure 14. Difference of Feature Effects Between PMTL and Other Models: A Case Study of Subgroup of Infect & Parasitic Disease eFigure 15. Effect Change of Factors Estimated by Subgroup Models and Meta-Regression on PMTL eFigure 16. Comparison of Coefficients Estimated by PMTL and Subgroup Models, Cases of Age, Serum Calcium and Blood Glucose eFigure 17. Co [file jamanetwopen-e2219776-s001.pdf]

# Supplemental Online Content

Liu K, Zhang X, Chen W, et al. Development and validation of a personalized model with transfer learning for acute kidney injury risk estimation using electronic health records. *JAMA Netw Open*. 2022;5(7):e2219776. doi:10.1001/jamanetworkopen.2022.19776

**eAppendix 1.** Data Extraction
**eAppendix 2.** Personalized Model With Transfer Learning (PMTL)
**eAppendix 3.** Mechanism of Transfer Learning
**eAppendix 4.** Detail of Benchmarking Models
**eAppendix 5.** Statistical Analysis
**eAppendix 6.** Predictor Importance Estimation
**eAppendix 7.** Interaction Analysis for Important Predictors in PMTL
**eFigure 1.** Validation Scheme of This Study
**eFigure 2.** AUPRC Comparison of Personalized Models With and Without Similarity Measure Learning in General Patients Based on 4 of 5 Data Folds Used for Testing
**eFigure 3.** Model Discrimination Comparison in General Inpatients
**eFigure 4.** Overall Performance of Personalized and Subgroups Modeling In and Out of Top 20 High-Risk Subgroup
**eFigure 5.** Calibration of Global, Subgroup and Personalized Model in Each of Top 20 High-Risk Subgroup
**eFigure 6.** Radar Chart of AUROC Comparison for Subgroup Models With Transfer Learning
**eFigure 7.** Absolute Pearson Correlation Coefficient Among Top-50 Important Predictors in Different Subgroups
**eFigure 8.** Average Value Changes of Top-20 Predictors Determined by Global Model in Subgroups
**eFigure 9.** Standard Deviation Changes of Top-20 Predictors Determined by Global Model in Subgroups
**eFigure 10.** Wasserstein Distance of Top-20 Predictor Distribution Between Patients in General and Subgroups
**eFigure 11.** Top 20 Predictors Where Their Effects Differed Between Global Model and PMTL
**eFigure 12.** Coefficient of Variation of the Regression Coefficients of Top-200 Features in PMTL
**eFigure 13.** Difference of Feature Effects Between PMTL and Other Models: A Case Study of Subgroup of Cardiac Valve Procedure With Cardiac Catheterization
**eFigure 14.** Difference of Feature Effects Between PMTL and Other Models: A Case Study of Subgroup of Infect & Parasitic Disease
**eFigure 15.** Effect Change of Factors Estimated by Subgroup Models and Meta-Regression on PMTL
**eFigure 16.** Comparison of Coefficients Estimated by PMTL and Subgroup Models, Cases of Age, Serum Calcium and Blood Glucose
**eFigure 17.** Comparison of Coefficients Estimated by PMTL and Subgroup Models, Cases of BMI, Pulse and Vancomycin
**eTable 1.** Variables Used in This Study
**eTable 2.** Characteristics of Patients
**eTable 3.** AUROC of Different Models in Validation Set
**eTable 4.** AUROC of Personalized Model Among Similar Samples in Validation Set
**eTable 5.** Performance of Similarity Measure Learning in Test Set
**eTable 6.** Performance of Sample Weighting in Test Set
**eTable 7.** Performance (AUROC) of Feature Selection in Dealing With Overfitting in Personalized Modeling
**eTable 8.** List of APR-DRG Selected as High-Risk Subgroups
**eTable 9.** AUROC of Models in 31 High Risk DRG Subgroups With At Least 20 AKI Patients
**eTable 10.** AUPRC of Models in 31 High Risk DRG Subgroups With At Least 20 AKI Patients

© 2022 Liu K et al. *JAMA Network Open.*

**eTable 11.** Superiority of PMTL in Recalling AKI Patients in All Top 20 High-Risk Subgroups
**eTable 12.** Superiority of PMTL in Recalling AKI Patients in Each of Top 20 High-Risk Subgroups
**eTable 13.** Details About 55 AKI Prediction Researches for Specific Subgroup Patients Can Be Identified by Our Data
**eTable 14.** AUPRC Comparison of Models in 20 Well-Studied Subgroups
**eTable 15.** List of APR-DRG Presented in Fig 4 of Main Text
**eTable 16.** Significant Interactions Between 6 Important Predictors and Disease in Meta-Regression and Their Verification
**eTable 17.** Significant Interactions Between 6 Important Predictors and Disease in Meta-Regression (Top-5 Mainly) and Their Verification
**eTable 18.** Top 50 Features in Patient Similarity Calculation
**eReferences**

This supplemental material has been provided by the authors to give readers additional information about their work.

© 2022 Liu K et al. *JAMA Network Open.*

## **eAppendix 1.** Data Extraction

For each inpatient encounter, we extracted 1892 structured EHR variables including demographics, vital signs, medications, medical history, admission diagnoses, and laboratory tests that represent comorbidities correlated with AKI[1] (eTable 1). We did not include SCr/eGFR as predictors because they determine the outcome. Medications were normalized to RxNorm ingredients. Admission diagnoses were represented using All Patients Refined Diagnosis Related Group (APR-DRG). Medical history was captured as major diagnoses in the Clinical Classifications Software (CCS, https://www.hcup-us.ahrq.gov/toolssoftware/ccs/ccs.jsp) for ICD-9-CM mapping.

The prediction point was 1-day prior to onset for AKI patients and 1-day prior to the last SCr record for non-AKI patients. Most recent vitals and lab values recorded before the prediction point were used. Vitals were categorized using standard ranges and missing values were treated as a unique category. Labs were categorized as “unknown”, “present-and-normal”, or “present-and-abnormal”. Medication exposure was a binary variable, "true" for medications taken within 7-days before the prediction point. Medical history was represented as presence/absence of a major diagnosis before the prediction point. Demographics and admission diagnoses were also binary variables.

Feature encoding of Vital and Laboratory Test

| Vitals | Feature value |
|---|---|
| BMI | |
| Unknown | 0 |
| < 18.5 Underweight | 1 |
| 18.5 – 24.9 Normal | 2 |
| 25.0 – 29.9 Overweight | 3 |
| > 30.0 Obese | 4 |
| Temperature | |
| Unknown | 0 |
| < 95.0 Hypothermia | 1 |
| 97.7 – 99.5 Normal | 2 |
| 99.5 – 100.9 Fever | 3 |
| > 104.0 Hyperpyrexia | 4 |
| 95.0 – 97.7 low body temperature | 5 |
| Pulse (resting) | |
| Unknown | 0 |
| 50 – 65 Excellent | 1 |
| 66 – 80 Average | 2 |
| 81 – 100 Poor | 3 |
| < 50 | 4 |
| > 100 | 5 |
| BP (Systolic / diastolic) | |
| Unknown | 0 |
| < 120 / < 80 Normal | 1 |
| 120 – 139 / 80 – 89 Prehypertension | 2 |
| 140 – 159 / 90 – 99 Stage 1 hypertension | 3 |
| > 160 / > 100 Stage 2 hypertension | 4 |
| Laboratory Test | |
| Unknowns | 0 |
| Normal values | 1 |
| Abnormal values | 2 |

© 2022 Liu K et al. *JAMA Network Open.*

**eAppendix 2.** Personalized Model With Transfer Learning (PMTL)

PMTL (as the figure below) mainly contains four modules: 1) Similar Sample Matching – identify similar patients for a target patient; 2) Transfer Learning – leverage general knowledge learned from the global model to initialize training of personalized models[2]; 3) Personalized Modeling – continue learning from data on similar patients; and 4) Similarity Measure Optimization – optimize similarity measures used in Similar Sample Matching. To identify similar patients for each target patient, we applied k-nearest neighbor (k-NN) algorithm and calculated distances between patients using all 1892 variables. Each variable in the distance calculation was weighted by the Similarity Measure Optimization module that iteratively optimizes weights based on performance of personalized models on the training set. To address the diminishing sample size after Similar Sample Matching, we leveraged transfer learning. We used logistic regression (LR) as the base learner in PMTL and leveraged the global solution as a starting point for individual assessment by initializing coefficient of each variable in the personalized LR with corresponding feature's coefficient learned from the global model. More details of PMTL are as follow:

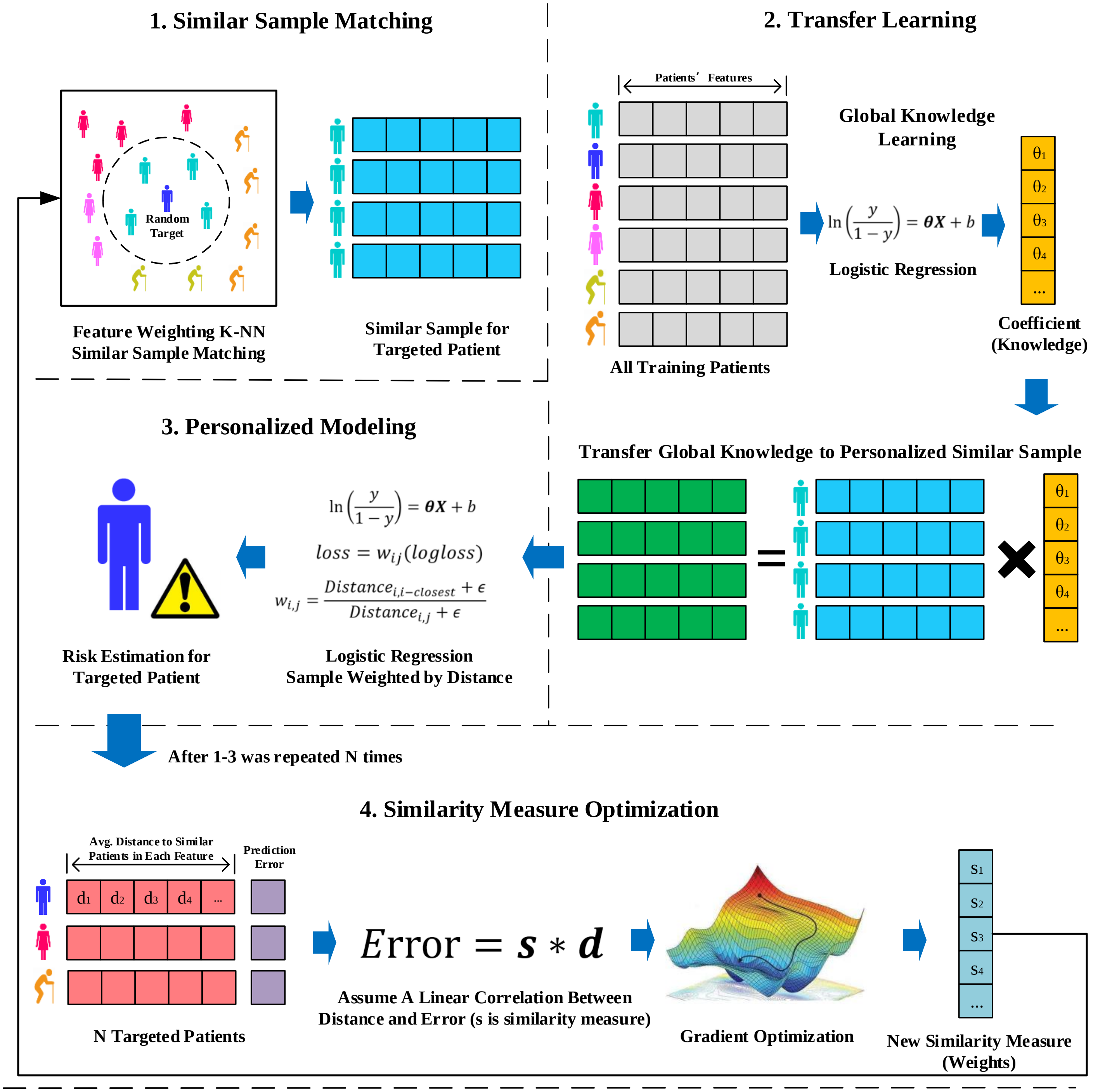


© 2022 Liu K et al. *JAMA Network Open.*

**Similar sample matching:** we utilized k-NN approach[3] for finding similar samples with all 1892 features. However, not all features are of the same importance, thus, k-NN considers weights of features when calculating distances among samples.

$$Similarity\ Meausre = (s_1, s_2, s_3 \dots s_m) \quad (1)$$

$$Distance_{i,j} = \sum_{n=1}^{m} s_n |x_{i,n} - x_{j,n}| \quad (2)$$

Here, $s$ represents the weights of the features when calculating the distance between samples, $m$ represents number of features used for similarity sample matching, $Distance_{i,j}$ represents the distance between samples i and j, and $x_{i,n}$ represents the nth feature of sample i. We used Manhattan distance to reduce the computational complexity during optimization. As k-NN means top k nearest neighbors, the first k samples closest to the target patient form a similar sample set.

**Knowledge transfer:** To cope with overfitting caused by insufficient sample size, transfer learning has been widely used[2,4]. Its main idea is to utilize knowledge in a similar domain (source domain) to help modeling in the target domain (i.e. personalized model in this study)[2]. Source domain should be as relevant or similar as possible to the target domain and has enough samples to learn accurate knowledge. Thus, we selected the global model, i.e., model trained based on all training samples, as the source domain. In this phase, we used all training samples to establish a global logistic regression model. The knowledge to be transferred is the coefficients of each feature in global model.

To achieve transfer learning between global and personalized logistic regression, we proposed a method inspired by widely used Finetune method for deep learning. Specifically, logistic regression is regarded as a special case of neural network, i.e., a neural network with only one hidden layer (Supplementary Text 3). Thus, the transfer learning method is similar to Finetune for logistic regression, i.e., multiplying the sample value of each feature by the regression coefficient of the feature in the global logistic regression model:

$$\boldsymbol{x}' = \boldsymbol{\theta}_{\boldsymbol{global}}\boldsymbol{x} \quad (3)$$

Here $\boldsymbol{x}$ refers to the feature vector of the original sample, $\boldsymbol{x}'$ refers to feature vector after transfer learning, and $\boldsymbol{\theta}_{\boldsymbol{global}}$ is the coefficient vector of features in the global logistic regression model, i.e., the knowledge to be transferred. Proposed transfer learning can provide a warm start for model training, that is tune learning speed and regularization loss for each feature based on its importance in the source domain. We explained these mechanisms in detail and presented experiment results in Supplementary Text 3.

**Personalized modeling:** Personalized logistic regression model for a patient is trained with the k similar sample selected by k-NN in similar sample matching. As samples more similar to the target patient would carry more valuable information for the risk estimation, sample weighting is considered when calculating the log-loss function. The weight of the training sample depends on its distance from the target sample, i.e.:

$$logloss = \sum_{j=1}^{k} w_{i,j} \left[ y_j \log h_\theta(x_j) + (1 - y_j) \log\left(1 - h_\theta(x_j)\right) \right] \quad (4)$$

$$w_{i,j} = \frac{Distance_{i,i\_closest} + \epsilon}{Distance_{i,j} + \epsilon} \quad (5)$$

Here k represents k similar samples selected by k-NN, and $w_{i,j}$ refers to the weight of similar samples j when modeling personalized logistic regression for target patient i. $h_\theta(x_j)$ is the predicted probability of the personalized logistic regression model for similar sample j. $Distance_{i,i\_closest}$ refers to the distance between the target sample i and its most similar sample. $\epsilon$ is a very small value to prevent the numerator or denominator from being 0.

**Similarity measure optimization:** In each iteration of the training process, after personalized models for each randomly selected target patient were built, we evaluated performance of the personalized models based on current similarity measure:

$$Error = (h_\theta(x_i) - y_i)^2 \quad (6)$$

As personalized models are built based on similar samples calculated using the current similarity measure, we assume that Error is generated by the mismatch of similar samples for the target patient. To identify predictors of the mismatch, we calculated the average difference between each target sample and its similar samples for each feature, i.e.:

© 2022 Liu K et al. *JAMA Network Open.*

$$\boldsymbol{d}_i = [E(x_{i,1} - x_{i-similar,1}), E(x_{i,2} - x_{i-similar,2}) \ldots E(x_{i,m} - x_{i-similar,m})] \quad (7)$$

Here $\boldsymbol{d}_i$ records the average distance between the target patient i and all its similar samples i-similar for each feature, and $E(x_{i,m} - x_{i-similar,m})$ refers to the average distance for the mth feature. For the interpretability of the measure and the complexity of optimization, we assume that the estimated Error for a target patient is linearly related to the average distance between the target and its similar sample, i.e.

$$Error = f(\boldsymbol{d}) = \boldsymbol{s} * \boldsymbol{d} \quad (8)$$

Here $\boldsymbol{s}$ represents the coefficient of $\boldsymbol{d}$, which reflects how many estimation errors will be generated when average distance on each feature changed. These coefficients can also reflect importance of features in sample matching. Therefore, the final similarity measure can be determined if we can determine $\boldsymbol{s}$. Different from existing metric learning methods for classification tasks[5], after each iteration, we need to update the similarity measure $\boldsymbol{s}$ by rematching similar samples and rebuilding personalized model to reevaluate current similarity measure, which led to high complexity of similarity measure optimization. Thus, an efficient gradient method was used to optimize the similarity measure. The optimization target is:

$$Min(\sum Error + \frac{c}{2}\boldsymbol{s}^2) \quad (9)$$

Where $\sum Error$ is the sum of the errors of the personalized models over all target samples, $\frac{c}{2}\boldsymbol{s}^2$ is the regularization term, and c is the regularization strength. Specifically, in the (n+1)th iteration of training, we would randomly select N samples from the training samples as target samples, and then match similar samples and build personalized models for each target using current similarity measure. After that, based on each target's estimation Error and its average distance $\boldsymbol{d}$ to its similar sample, we would update similarity measure $\boldsymbol{s}$ using Batch Gradient Descent:

$$\boldsymbol{s}_{n+1} = \boldsymbol{s}_n + \alpha(\frac{1}{N}\sum_{i=1}^{N}(Error_{i,n} - f(\boldsymbol{d}_{i,n}))\boldsymbol{d}_{i,n} - c\boldsymbol{s}_n \quad (10)$$

Considering the nature of the similarity measure $\boldsymbol{s}$, weight of each feature $s$ in similarity measure should fulfill $s \geq 0$. Therefore, after each iteration of similarity measure optimization, if weights of a feature m, i.e., $s_m < 0$, we believe that is the result of overfitting. Therefore, at the end of each iteration:

$$s_m = \begin{cases} s_m, if\ s_m \geq 0 \\ 0, if\ s_m < 0 \end{cases} \quad (11)$$

**Setting of PMTL:** Logistic regression was performed using Python version 3.7.4 and scikit-learn package version 0.19.2 with default hyper-parameters. In the process of sample weighting for the weighted logistic regression, minimum $\epsilon$ was set to 0.01. According to results on the validation set, tuned hyperparameters by the gradient approach for similarity measure learning were learning rate 0.01, batch size 1000, regularization strength 0.05, and times of iteration 50. According to results showed in eTable 4 & 5, initial weights of features in similarity measure are based on their coefficients in the global logistic regression model, i.e., absolute value of each feature's coefficient divided by the sum of absolute value of all coefficients. Considering the size of the total samples and the major types of AKI mechanisms, we evaluated the size of similar samples matched by k-NN as 20%, 10%, and 5% of training sample, and PMTL trained with 10% of training sample perform best in validation set.

© 2022 Liu K et al. *JAMA Network Open.*

**eAppendix 3.** Mechanism of Transfer Learning

As mentioned in Materials and Methods section, proposed transfer learning (as the figure below) can provide a warm start for modeling, increase learning speed, and tune regularization loss for each feature based on its importance in source domain. Here, we explain these mechanisms in detail.

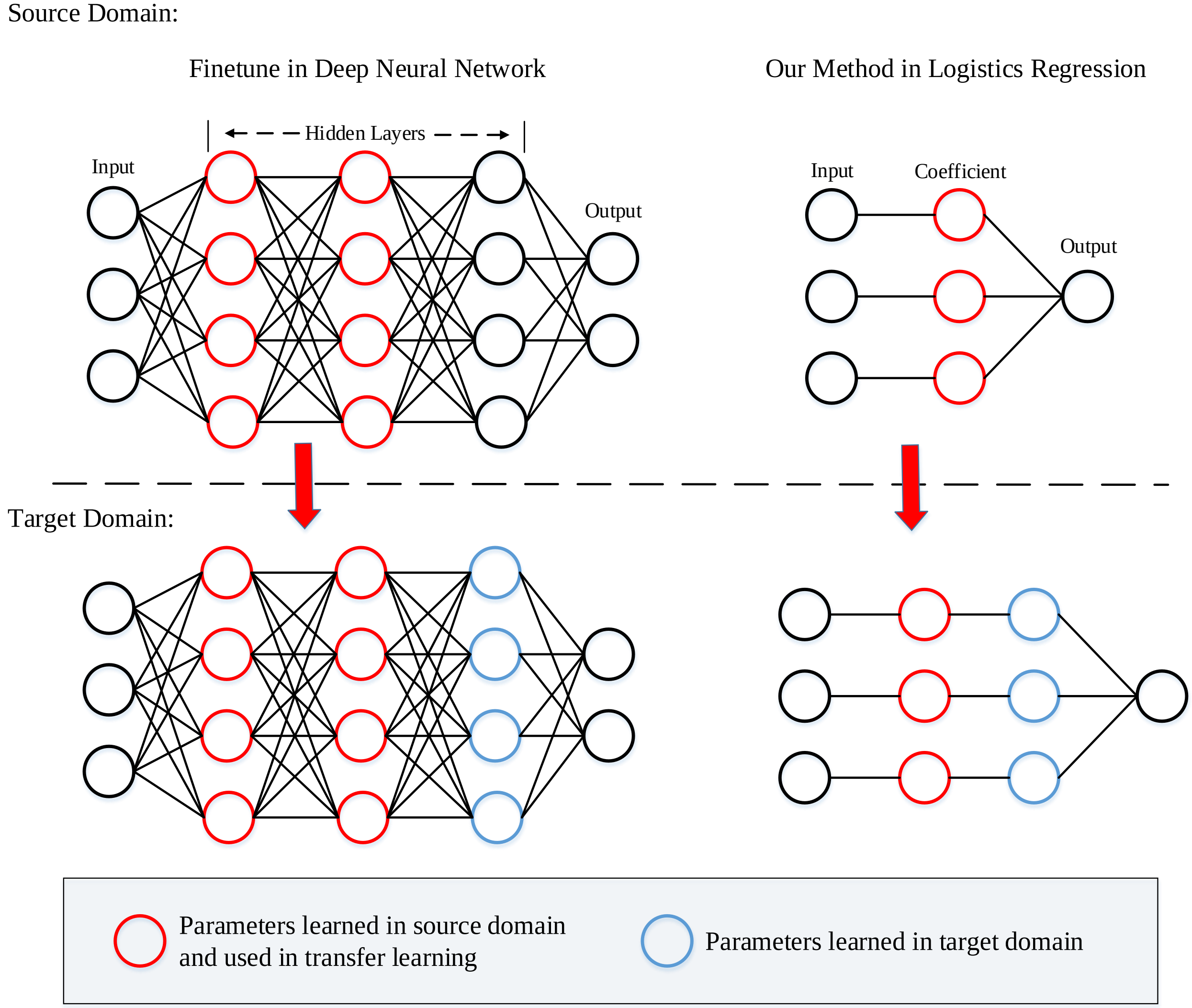


**Transfer learning provides a warm start for modeling:** It is straight forward that proposed transfer learning approach can provide a warm start for modeling as data in target domain is multiplied by coefficients in source domain (eFigure 2). If coefficients were not updated based on target domain, then sum of features value after transfer learning would be equal to prediction score generated by model in source domain.

**Transfer learning tunes regularization strengths of features:** Mechanism for tuning regularization loss can be understood through the following case. Suppose a logistic regression model is:

$$y = ax \tag{12}$$

Here $a$ is coefficient of predictor $x$ when predicting $y$. Then we multiply $x$ by $b$, i.e. $x' = bx$. To keep the two sides of the equation equal, equation should be modified to:

$$y = (\frac{1}{b}a)x' \tag{13}$$

That means new coefficient $a' = \frac{1}{b}a$. In the case of multiple variable regression, it is:

$$y = \left(\frac{1}{b_1}a_1\right)x_1' + \left(\frac{1}{b_2}a_2\right)x_2' + \cdots \tag{14}$$

The optimization objective of logistic regression is to minimize:

$$loss = logloss + c * regularization\ loss \quad (15)$$

Here $logloss$ measured the prediction performance of model in training set, and $regularization\ loss$ measured the complexity of coefficients. L1 regularization is $\sum |a_i|$, and L2 regularization (used in this study) is:

$$regularization\ loss = \sum a_i^2 \quad (16)$$

And $c$ tunes the weight of $regularization\ loss$ in optimization. Higher complexity of coefficients means more predictors are considered by a model. Ideally, the model can adapt to more complex situation, training performance will increase; but in many cases, it will cause overfitting, many ineffective factors are considered, and model performance decrease significantly in test set. Regularization loss is a common approach to avoid overfitting by punishing complexity of model.

Returning to our transfer learning, suppose vector $\boldsymbol{a} = (a_1, a_2, \ldots a_i)$ is the optimized coefficients for model in target domain and $\boldsymbol{b} = (b_1, b_2, \ldots b_i)$ is coefficients of model in source domain. Then, to keep optimized coefficients in target domain, regularization loss will tune to $\sum |\frac{1}{b_i} a_i|$ for L1 regularization, and L2 regularization used in this study is:

$$regularization\ loss = \sum (\frac{1}{b_i} a_i)^2 \quad (17)$$

We can observe that if a predictor has a higher absolute value of coefficient in source domain model, its regularization loss will be smaller when modeling in target domain. That means model will pay less attention to coefficient complexity of this predictor.

**Transfer learning tune learning speeds of features:** To explain how transfer tune learning speed for each feature, we take gradient optimization, a common and classic coefficient learning approach for logistic regression, as an example. In each iteration, change of coefficient of factor $i$ based on training on $n$ sample is:

$$\Delta_i = \omega * \sum_{j=1}^{n} (y_{true}^j - y_{predict}^j) x_i^j - c * a_j \quad (18)$$

Here, $\omega * \sum_{j=1}^{n} (y_{true}^j - y_{predict}^j) x_i^j$ is designed to optimized coefficients based on prediction performance according to gradient of logloss, $y_{true}^j$ is true classification (i.e. with or without AKI) of sample $j$, and $y_{predict}^j$ is prediction probability generated by logistic regression, $\omega$ is learning rate for all factors. $c * a_j$ is gradient of regularization loss as we have mentioned above. And after transfer learning, i.e. $x'^j_i = b_i x_i^j$, the $\Delta_i$ will change to:

$$\Delta'_i = \omega * \sum_{j=1}^{n} (y_{true}^j - y_{predict}^j) b_i x_i^j - c * a_j \quad (19)$$

We can observe that if a predictor has a higher absolute value of coefficient in source domain model, its learning speed will be higher in optimization, more gradient will be assigned to this factor, and its final coefficient is probably higher.

**Effect of transfer learning to overfitting:** To show the effect of transfer learning to overfitting, we tuned parameter of regularization strength (i.e., $c$) to estimated performance of subgroup models for top-20 high-risk subgroups under different model complexity (as the figure below, coefficients in models with transfer learning are calculated by multiplying coefficients in global model and coefficients in models for data after transfer learning). We observed two phenomena. First, comparing curves of models without transfer, the peak of curves of models with transfer learning moved towards the right upper portion. In other words, the optimized complexity of models and the best performance of models under optimized complexity were both higher in the case when transfer learning is considered. That means after overfitting is mitigated by transfer learning, models can improve their performance by increasing their complexity. Second, when complexity of model excess optimized complexity (i.e. overfitting), models with transfer learning always perform better in the same complexity. The outperformance of model with transfer learning increase with higher model complexity.

Above results show that by mitigating overfitting with transfer learning, model can perform better and become more robust to parameter of model complexity. Considering parameter tuning is always time consuming, this advantage is very important for personalized modeling.

© 2022 Liu K et al. *JAMA Network Open.*

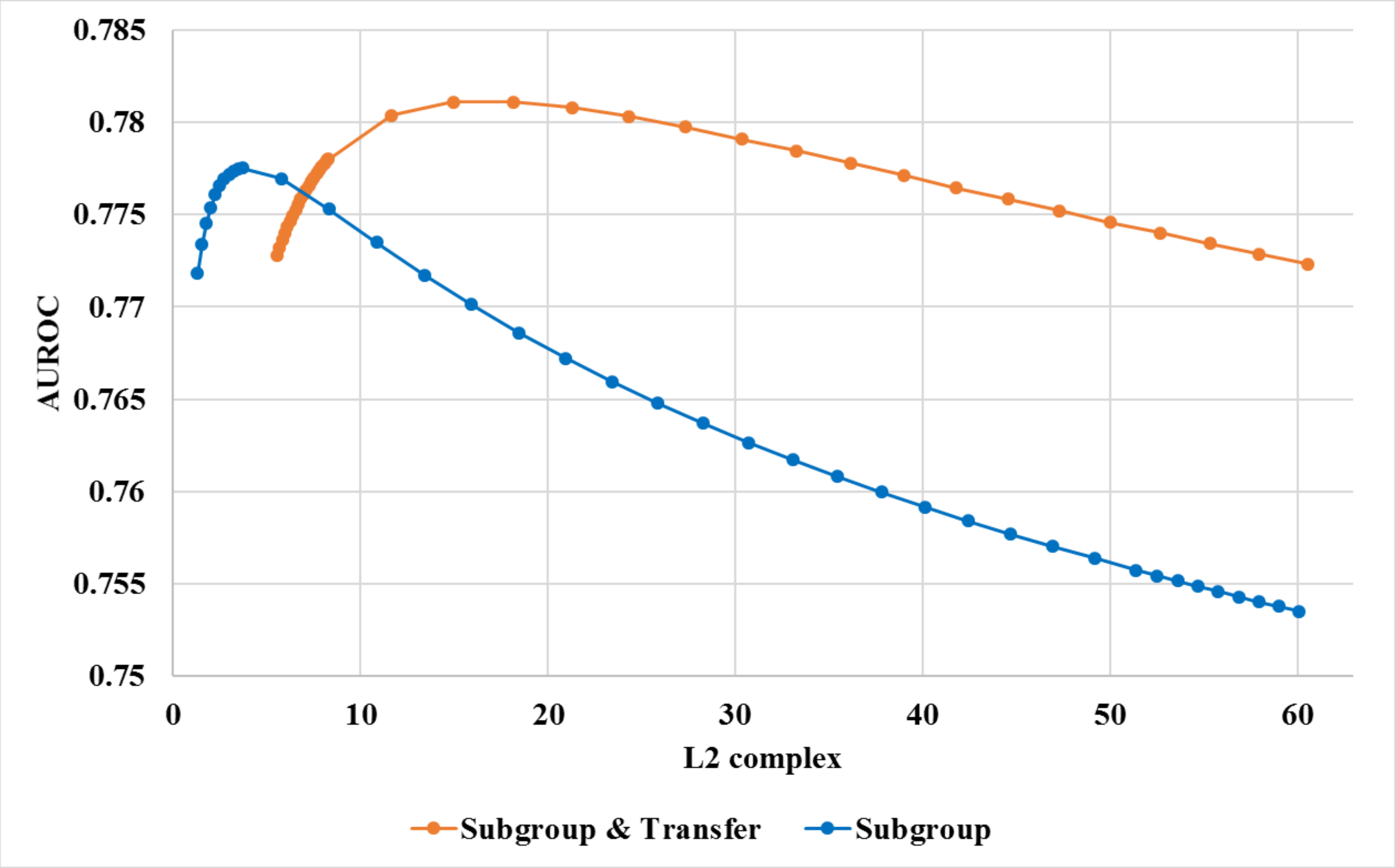


**Approach for tuning source domain used for transfer learning in highly heterogeneity subgroups (used in this subsection only).** According to Figure 2 and eFigure 6, effect of transfer learning with global model was small or even negative in some subgroups where global model significantly underperformed personalized or subgroup without transfer learning. That probably because patients in these subgroups were so different from general patients that knowledge learned in global could not guide their modeling. So, we tried to tune source domain for these subgroups. Our general idea (as the figure below) followed the classical transfer learning approach: samples of general patients are weighted based on their estimation error generated by subgroup model before transfer leaning. A sample with less estimation error was assigned to higher weight because it is probably more similar to target domain. And model for source domain was built with the weighted samples. However, considering subgroup models with poor discrimination cannot effectively determine which sample is more similar, the strength of weighting is moderated by AUROC of the subgroup model.

Performance of this approach is showed in the radar chart below. After tuning source domain, the overall AUROC of subgroup model in top 20 high-risk subgroup increase from 0.778 to 0.789, p=0.0001; and the overall AUROC of PMTL also increase from 0.792 to 0.801, p<0.0001, p=0.0008 for comparison PMTL and subgroup model with transfer learning in this case. The improvement of both mainly come from the subgroups where transfer learning performed poor when using global model as source domain.

However, this approach is not used in other experiments in main test or supplement because we want to develop personalized model for general patients no matter if we have accurately known which subgroups they should be classified into.

© 2022 Liu K et al. *JAMA Network Open.*

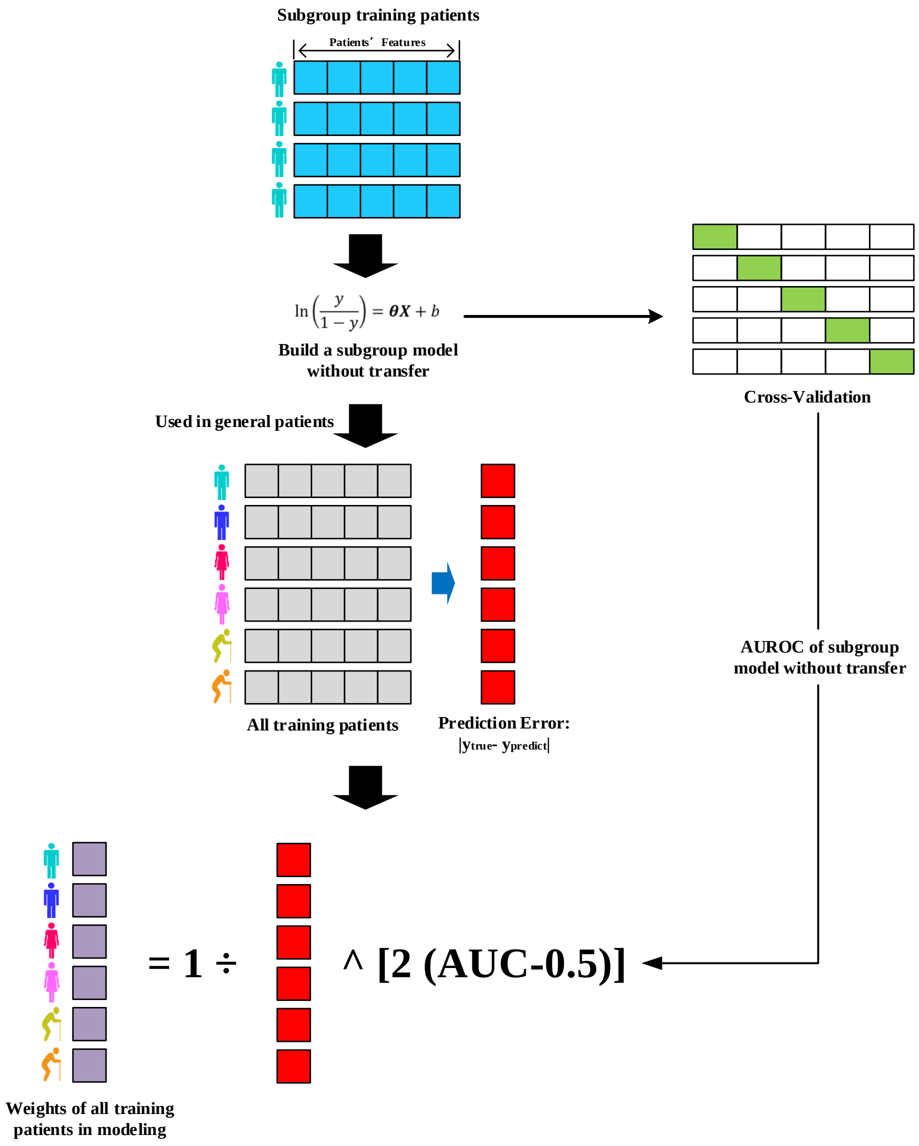


© 2022 Liu K et al. *JAMA Network Open.*

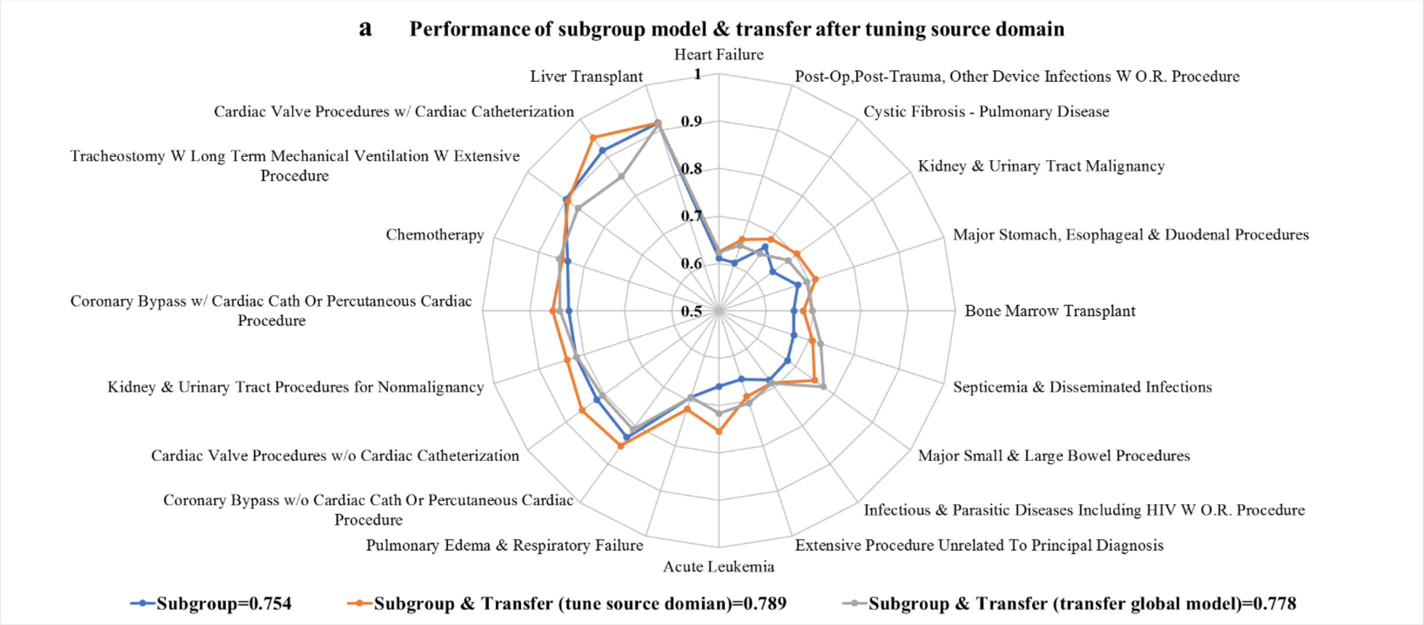


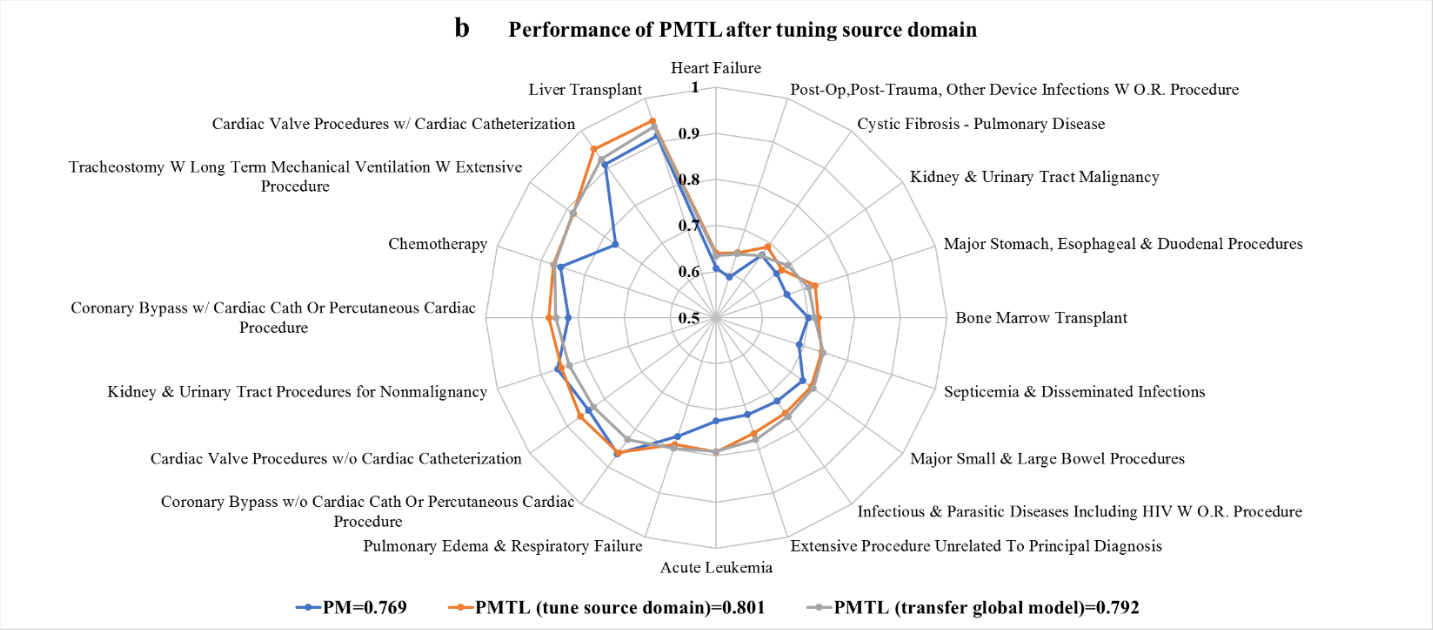


© 2022 Liu K et al. *JAMA Network Open*.

**eAppendix 4.** Detail of Benchmarking Models

- Global Model (GM): Logistic regression model built with all training sample, or with a percentage of the whole training sample based on random sampling (result in Figure 1a).
- Global Model with Transfer Learning (GMTL): Coefficients of global logistic regression model built with 100% sample were used to initialize the whole training set or a percentage of the whole training sample according to Supplement Text 2. And global logistic regression model was built with the modified training data.
- Subgroup Model (SM): Logistic regression model built with training sample classified to a subgroup.
- Subgroup Model with Transfer Learning (SMTL): Coefficients of global logistic regression model built with 100% sample were used to initialize the training sample of the subgroup, and the subgroup logistic regression model was built with the modified training data
- Personalized Model (PM): For each test sample, a percentage of all training sample (general patients) with highest similarity to the target test sample is matched, and a personalized logistic regression was built specifically for the target test sample based on the matched similar sample.
- Personalized Model with Transfer Learning (PMTL): For each test sample, a percentage of all training sample (general patients) with highest similarity to the target test sample is matched, coefficients of global logistic regression model built with 100% sample were used to initialize the matched similar sample, and a personalized logistic regression was built specifically for the target test sample based on the matched similar sample.

© 2022 Liu K et al. *JAMA Network Open.*

## **eAppendix 5.** Statistical Analysis

Model performance was measured by area-under-the-receiver-operating-characteristic-curve (AUROC), area-under-the-precision-recall-curve (AUPRC), and calibration (brier score). Significance of AUROC comparison on our data was calculated using Delong test[6]. Delong test is the default AUROC comparison method in popular medical data analysis software MedCalc and also commonly used in other medical prediction modeling studies[7,8].

However, Delong test cannot be used in comparison between PMTL and previous models as we do not have access to the raw data from the published studies. However, according to basic concept of Delong test, we can estimate the p value based on Z-test (two-side) in the case AUROC variation of the two model is known. Thus, we estimated AUROC variation of PMTL based on Delong test, while AUROCs and their variation of previous models were based on reported AUROC and its 95%CI in literatures, covariations of model performances were not considered in this case, i.e.:

$$z = \frac{AUROC_A - AUROC_B}{\sqrt{AUROC\_var_A + AUROC\_var_B}} \quad (20)$$

The AUPRC comparisons were based on Z-test (two-sided). variation of AUPRC and covariation of AUPRC between models were calculated by resampling (with replacement) test data and recalculating the model performance 200 time. The final Z-score between two models was calculated as:

$$z = \frac{AUPRC_A - AUPRC_B}{\sqrt{AUPRC\_var_A + AUPRC\_var_B - 2AUPRC\_covar_{AB}}} \quad (21)$$

To justify the choices of the Z-test, we used normal test (null hypothesis: a sample comes from a normal distribution; based on python package: scipy.stats.normaltest) to test variation of AUPRC with resampling (with replacement) test data. We found the null hypothesis cannot be reject in most cases (see Table below, each experiment was repeated 10 times).

| Population | Model | Times of H0 is rejected (repeated 10 times) | Avg p |
|---|---|---|---|
| General patients | Global model | 1 (10%) | 0.54 |
| | PMTL | 1 (10%) | 0.46 |
| | PM | 0 | 0.47 |
| | PM-kNN | 0 | 0.42 |
| | PM-kNN & TL | 2 (20%) | 0.33 |
| | PM-kNN & WS | 0 | 0.43 |
| | PM-kNN & TL & WS | 3 (30%) | 0.37 |
| Top 20 high-risk subgroups | Global model | 1 (10%) | 0.47 |
| | PMTL | 1 (10%) | 0.48 |
| | PM | 0 | 0.46 |
| | Subgroup model | 0 | 0.62 |
| | Subgroup model & Transfer | 0 | 0.57 |
| | PM-kNN | 1 (10%) | 0.41 |
| | PM-kNN & TL | 0 | 0.42 |
| | PM-kNN & WS | 2 (20%) | 0.47 |
| | PM-kNN & TL & WS | 0 | 0.43 |

Variation of brier score with resampling (with replacement) test data cannot past normal test. Thus, we directly estimate the significance of difference between two model based on bootstrapping test sample (repeated 20,000 times). Specifically, p value of brier score difference between model A and B is calculated as:

$$p = \begin{cases} 2 * possibility_{A>B} & if\ possibility_{A>B} < 0.5 \\ 2 * (1 - possibility_{A>B}) & if\ possibility_{A>B} \geq 0.5 \end{cases} \quad (22)$$

© 2022 Liu K et al. *JAMA Network Open.*

Significance of result in meta-regression were based on p-value returned by PyMARE package. Significance of predictors effect changes in two subpopulations were calculated by Z-test [9,10] similar to equation (20), effects of predictors were calculated based on coefficients of logistic regression model or log of OR calculated with raw data directly, and variation of predictors effect was calculated based on resampling (with replacement) training data and rebuilding models 200 times.

© 2022 Liu K et al. *JAMA Network Open.*

**eAppendix 6.** Predictor Importance Estimation

We used three indicators to measure effect change of predictors in heterogeneous patients. The first indicator was coefficients of predictors in the logistic regression models, which directly reflects effect of predictors estimated by the prediction model. The second was AUROC gain of predictors, calculated by AUROC change when a predictor is removed comparing against AUROC of the original model. The third was inter-class score difference [11]. The direct output of logistic regression model for each patient is a prediction score, equal to log of OR (odds ratio) of the predicted risk. Thus, average predicted score in positive samples should be higher than negative samples. And inter-class score difference measures change of average score difference between positive and negative sample when a predictor is removed, which is somewhat similar to the Integrated Discrimination Improvement (IDI) indicator.

Predictor coefficient estimates effect of a predictor on an individual level but does not reflect its importance at a population level because sample size of the predictor is ignored. Since the coefficient estimation may be inaccurate when sample size is small and patients are heterogeneous, it is suitable for estimating effect change of important predictors in PMTL but not suitable for the global model.

Both AUROC gain and inter-class score difference reflect importance of predictors on a population level. AUROC gain is more intuitive but it is non-additive (sum of AUROC gain of all predictors is not equal to AUROC of the final model). Inter-class score difference is additive and can be used to measure cumulative effect of predictors.

© 2022 Liu K et al. *JAMA Network Open.*

## **eAppendix 7.** Interaction Analysis for Important Predictors in PMTL

To understand interactions of risk factors in the personalized models, we performed meta-regression using PyMARE: Python Meta-Analysis & Regression Engine (https://pymare.readthedocs.io/en/latest/). Each personalized model built for a target patient was treated as an independent study. The study-level effect size of each target variable was calculated based on its coefficients from the personalized models of patients that had the variable recorded. There are two rationales behind this. First, coefficient of a factor and its changes are meaningful only when the factor information is recorded for the target patient. Second, due to serious data imbalance in medical data, coefficient of a factor in a personalized model may "unexpectedly" be 0 just because a factor is missing in similar samples. The remaining variables of target patients were treated as study-level covariates because similar patients are matched based on those factors for the target patients. We did not use average value of the variables in similar patients as covariates because many factors may occur in similar samples but not in the target sample, thus many false positives may result from analysis based on averaging across samples. Further averaging across samples would increase multicollinearity among factors.

We observed that coefficients of diseases in meta-regression are often insignificant when drug information is considered because of the small sample size of admission diagnoses and collinearity between diseases and drugs. Thus, we implemented two strategies for meta-regression analyses. The first strategy was to examine potential interactions between target predictors and diseases. Thus, we excluded the 1271 medication variables, and performed meta-regression on the remaining 621 variables of demographics, vital signs, lab test, admission diagnosis, and medical history. The second strategy was to examine the potential interactions of target predictors with drugs or conditions related to drugs, and all features were considered.

With literature review, not all significant interactions are known and have been studied in existing research. However, different personalized models are not completely independent because they may share subset of similar patients. So, we used subgroup analysis to verify the interactions found by meta-regression. Specifically, we divided patients into different subgroups by controlling moderator found by meta-regression and compared the effect of target predictor between patients exposed to the moderator and the remaining according to its coefficient in logistic regression model (subgroup model in eTable 17 & 18) or odds ratio (OR) calculated from the raw data directly (subgroup analysis in eTable 17 & 18).

A major challenge in subgroup analysis for interaction between disease and target predictors (eTable 17) is the limited number of samples. So, we aggregated similar significant admission diagnoses into large subgroups. To find more significant admission diagnoses and improve the sample size, the threshold of significance was set to $p<0.01$ for single variable meta-regression analysis and $p<0.05$ for multiple variable analysis. Although many potential interactions are found, we primarily verified the large subgroups containing many significant results or show high effect (measure by estimates) in meta-regression. In cases where we are not sure to which large subgroup an admission diagnosis belongs, we performed analyses for multiple potential subgroups. In several cases where many similar admission diagnoses were significant in meta-regression, but result is not significant in the subgroup model (probably due to limited sample size), we had to include insignificant admission diagnoses that are similar to the significant ones to increase sample size.

To verified interactions between drugs and target predictors, controlling effects of diseases is necessary (eTable 18). If a drug was frequently used in patients with specific admission diagnoses, we divided patients into different subgroups by controlling the admission diagnoses. In meta-regression with medication information, the threshold of significance was set to $p<0.01$ for both single and multiple variable analysis. Although many potential interactions are found, as many potential subgroups need to be controlled, we mainly presented top-5 results that show highest effect in meta-regression (measure by estimates) or interesting interactions.

**EFFECT OF AGE IN HETEROGENEOUS PATIENTS**

**Diseases related to coefficient change of age:** Top-5 admission diagnoses correlated with effect improvement of age are cardiac surgeries (eTable 17). The result is also significantly verified by subgroup model and subgroup analysis. Meta-regression also showed other admission diagnoses for cardiovascular conditions to be related to effect improvement of age, but its effect was not supported by subgroup model nor subgroup analysis. This may be due to similar samples used for training personalized model for these patients containing many patients who had cardiac surgeries.

Previous research have shown infection induced AKI is more common in older adults [12,13]. However, we found coefficient of age decreased in infection patients. In the meta-regression without medication, 11 admission diagnoses of infection were significant and 4 of 11 are in Top-10. Results were also verified by subgroup model and subgroup analysis even we only consider patients of “Septicemia & Disseminated Infections”.

“Bone Marrow Transplant” is the number one admission diagnoses related to decreasing coefficient of age. Other two types of admissions for major hematological disease were also significant. They were also significant in subgroup model and subgroup

© 2022 Liu K et al. *JAMA Network Open.*

analysis. Generally speaking, outcome of bone marrow transplant is poorer in older patients, and these results may be due to the higher rate of autologous transplantation in older patients [14]. However, our data didn't record the specific type of bone marrow transplant.

**Medication related to coefficient change of age:** Aldesleukin, mainly used in chemotherapy, is the medication found to be related to effect improvement of age. When we investigated the relationship within patients on chemotherapy, the interaction was still significant in subgroup model.

Two amino acids antifibrinolytics also significantly related to effect improvement of age in meta-regression. But they are highly related to cardiac surgery. And when we verified the result in major cardiac surgery, interesting differences between them were found. As shown in eTable 18, aminocaproic acid is related effect improvement of age (verified by both subgroup model and subgroup analysis, and p=0.06 in subgroup model). However, tranexamic acid was related to decreasing effect of age (significant in subgroup model). Age distributions were nearly the same between patients who used the two drugs.

Moreover, antibiotics were significantly related to decreasing effect of age. Although not all results were significant in subgroup model, directions of the effect change were consistent in different subgroups for different antibiotics. Furthermore, used of glucose also significantly related to decreasing effect of age. It may indicate patients have no serious diabetes, a common risk factor for the older.

## EFFECT OF SERUM CALCIUM IN HETEROGENEOUS PATIENTS

**Diseases related to coefficient change of serum calcium:** It is known that patients with abnormal serum calcium may present with various clinical signs and symptoms include cardiovascular manifestations. In meta-regression, the Top-6 admission diagnoses related to effect improvement of serum calcium are cardiac surgery. It is also significant in both subgroup model and subgroup analysis. Effect of serum calcium also increased in admissions with mechanical ventilation according to the results of all experiments. And a recent study showed patients with lower serum ionized calcium is associated with higher risk of acute respiratory failure [15]. Burn is also related to increased effect of serum calcium. Hypocalcemia is commonly complicated with burn, its severity is related to the severity of burn [16,17]. And serious burn is also related to higher risk of infection, dehydration and hypoxia. Additionally, 7 major surgeries were also significant in meta-regression, and the result was only significant in subgroup analysis.

"Cardiac Catheterization for Ischemic Heart Disease" was found to be significantly related to decreasing effect of serum calcium in all experiments. As a comparison, "PCI w/o AMI" showed significant relation to effect improvement of serum calcium in meta-regression. Existing research shows the outcome of acute myocardial infarction is better in patients with higher serum calcium [18,19]. It is observed that the incidence rate of abnormal serum calcium is higher in admissions for liver diseases, orthopedic surgeries, and alimentary tract diseases. That may be because these conditions are related to absorption, decomposition, metabolism, and loss of calcium. However, the ORs of abnormal serum calcium decreased significantly in these subgroups. That means abnormal serum calcium may not increase the AKI risk. In addition, infection is a cause of hypocalcemia, but our experiment showed abnormal serum calcium will not increase AKI risk in infection patients. We found above results were supported by existing research [20,21].

**Medication related to coefficient change of serum calcium:** Some cardiovascular medications showed significant relation to effect improvement of serum calcium. When we assessed the result in major cardiac surgery, interesting difference between aminocaproic acid and tranexamic acid were found again. Aminocaproic acid was significantly related to effect decrease in serum calcium, while tranexamic acid was related to effect improvement of serum calcium. However, frequency of abnormal serum calcium is much higher in cardiac surgery patients exposed to aminocaproic acid. Other cardiovascular medications including prochlorperazine, protamine sulfate and atropine were also significant.

Aldesleukin is the most significant medication related to decreasing OR of serum calcium. Among patients who were exposed to aldesleukin and had normal serum calcium, AKI incidence rate is 93% (107/115); while the AKI incidence rate is only 32.2% (47/146) in patients who were exposed to aldesleukin and had abnormal serum calcium. Hypoalbuminemia is one of common side effects of aldesleukin. However, only ionized calcium, not calcium link albumin (protein-bound calcium), is physiologically active. Thus, we hypothesized that this phenomenon may be caused by two factors: 1) if calcium supplement is used to address abnormal serum calcium while taking aldesleukin, it can lead to excessive ionized calcium; 2) if hypoalbuminemia occurred while serum calcium is normal, it may mean ionized calcium is elevated. However, both hypotheses were not supported by subgroup analysis. First, in patients with normal serum calcium and used calcium supplement, AKI incidence rate is 88% (43/ 49); among patients with normal serum calcium and did not used calcium supplement, AKI incidence rate is 97% (64/ 66); in patients with abnormal serum calcium and used calcium supplement, AKI incidence rate is 25% (15/ 61); in patients with abnormal serum calcium and did not used calcium supplement, AKI incidence rate is 38% (32/ 85). Second, in patients with normal serum calcium and albumin, AKI incidence rate is 98% (63/ 64); in patients with normal serum calcium and abnormal albumin, AKI incidence rate is 86% (44/ 51); only 6 patients with abnormal serum calcium and normal albumin, 4 of them have AKI; in patients with abnormal serum calcium and albumin, AKI incidence rate is 31% (43/ 140). Therefore, the interaction between serum calcium and aldesleukin still need further study.

© 2022 Liu K et al. *JAMA Network Open.*

Oxycodone and Fondaparinux were also found to be related to decreasing OR of serum calcium, and their effect were verified in subgroup of patient who underwent joint replacement.

## AMINOCAPROIC ACID VS TRANEXAMIC ACID

In above analyses on age and serum calcium, we observed different effects between two types of amino acids antifibrinolytics: aminocaproic acid and tranexamic acid. Here, we aim to study the influence variation of aminocaproic acid and tranexamic acid on AKI incidence. Previous research compared the two drugs [22] and most studies compared the two drugs in cardiac surgery, and no significant difference in AKI incidence rate was found in most cases. However, in our data, among patients admitted for "Cardiac Valve Procedures w/ Cardiac Catheterization", "Cardiac Valve Procedures w/o Cardiac Catheterization", "Coronary Bypass w/ Cardiac Cath Or Percutaneous Cardiac Procedure", "Coronary Bypass w/o Cardiac Cath Or Percutaneous Cardiac Procedure", "Other Cardiothoracic Procedures", AKI incidence rate in patients used aminocaproic acid was 40/161 (24.8%), 84/ 92 (21.4%), 96/428 (22.4%), 96/ 407 (23.6%), 13/87 (14.9%); while in patients used tranexamic acid, the incidence rate was 7/22 (31.8%), 37/200 (18.5%), 18/124 (14.5%), 35/153 (22.9%), 2/21 (9.5%). AKI risk was higher in patients who used aminocaproic acid in 4/5 subgroups. In "Cardiac Valve Procedures", AKI incidence rates in those used aminocaproic acid and tranexamic acid were 22.4% and 19.8% ($p<0.025$); In "Coronary Bypass", AKI incidence rates were 23% and 19.1% ($p<0.0005$). After adjusting sample weight of patients who used aminocaproic acid based on admission distribution of patients who used tranexamic acid, AKI incidence rates in patients who used aminocaproic acid in the above two cardiac surgeries were 21.8% and 23.1%. This result indicates higher all-stage AKI risk in patients who used aminocaproic acid. After excluding patients admitted for major cardiac surgery and orthopedic surgery (1100 joint replacement patients exposed to tranexamic acid but only 1 exposed to aminocaproic acid), AKI risk was still higher in remaining patients who used aminocaproic acid (18.2% or 81/446 vs 9% or 29/321). Furthermore, we adjusted sample weight of patients using aminocaproic acid based on admission distribution of patients using tranexamic acid (if no patient use aminocaproic acid in a subgroup, we will exclude this subgroup in calculation of AKI risk), we found patients using aminocaproic acid still had higher AKI risk than those using tranexamic acid. If we did not exclude patients with major cardiac surgery, the difference was smaller but more significant (21.5%, se=0.96% vs 18.3%, se=1.63%, $p\approx0.05$); while excluding patients with major cardiac surgery, the difference was larger but less significant (18%, se=2% vs 13.5%, se=3.2%, $p\approx0.12$). The significance is probably influenced by small sample size. Drug combination also cannot explain the difference because only 4 patients used both drugs. The different result between this study and existing research may be due to following reasons: (1) Primary outcome of this study is all-stage AKI but it is stage-2 or higher or RRT (renal replacement therapy) in most existing research; (2) Many existing research compared the two drugs based on RCT (randomized controlled trial) while this is a retrospective study, so further analysis is warranted. Additionally, the different interaction effect of these two drugs with age and serum calcium was not studied in previous research and requires further research.

## EFFECT OF BLOOD GLUCOSE IN HETEROGENEOUS PATIENTS

**Diseases related to coefficient change of blood glucose:** Many surgeries were observed to be related to the effect improvement of blood glucose in personalized models. Liver transplant was the number one reason according to meta-regression. And "Major Pancreas, Liver & Shunt Procedures" was also significant. Their results were almost significant in subgroup model and subgroup analysis; thus, caution should be taken and further investigation is needed. Total 9 admission reasons of gastrointestinal surgery were significant in meta-regression, 4 of them were in top-10, their results were also significant in both subgroup model and subgroup analysis. Joint replacement was also significantly related to effect improvement of blood glucose in all experiments. Coefficients of blood glucose decreased significantly in non-surgery cardiovascular diagnoses, according to both meta-regression and subgroup model.

**Impact of different insulin on coefficient of blood glucose:** Different types of insulin show different interaction effects with blood glucose. In meta-regression, insulin,aspart, human/rdna is the top-1 medication related to effect improvement of blood glucose (estimate=0.03), effect of insulin regular,human buffered was much weaker (estimate=0.014), while effect of insulin, isophane was negative (estimate=-0.011). Results were supported by subgroup model, abnormal blood glucose is more dangerous in cardiac surgery patients exposed to insulin,aspart, human/rdna, and coefficient of abnormal blood glucose decrease significantly in patients used insulin, isophane in many situations. Results indicates blood glucose control strategy in different situations may need further concern.

**Other medication related to coefficient change of blood glucose:** Use of glucose, fentanyl, lactate, and benzoic acid showed related to effect improvement of blood glucose. Among them, glucose can directly influence blood glucose; the use of fentanyl may indicate postoperative analgesia or severe diseases. Benzoic acid belongs to salicylates which is risky for patients with renal insufficiency. Further, previous researches have shown that benzoic acid and its derivatives may influence glucose metabolism [23-25]. And recent studies show combined lactate and glucose levels related to renal dysfunction and mortality [26,27].

Paracetamol (includes acetaminophen) shows interesting interaction with blood glucose. In general, paracetamol is significant related to decrease coefficient of blood glucose. And existing research shows acetaminophen may influence glucose sensing [28,29]. However, paracetamol significantly related to effect improvement of blood glucose in patients of joint replacement. We failed to find out research studied their interaction.

© 2022 Liu K et al. *JAMA Network Open.*

## EFFECT OF BMI IN HETEROGENEOUS PATIENTS

**Diseases related to coefficient change of BMI:** Respiratory conditions were the most important factors related to increased effect of BMI according to meta-regression: totally 11 admission diagnoses were significant and 5 of them were in top-10. Existing research has summarized the complex interaction between obesity and respiratory diseases [30]. However, our result was not verified by subgroup model and subgroup, which may require further study to verify the interaction. Leukemia showed significant relation to higher effect of BMI, the result was more significant when patients is overweight. Existing meta-analysis studies have also shown obesity/overweight to be significantly related to incidence of leukemia and outcome of leukemia patients [31-34].

In meta-regression, top-4 admission diagnoses related to decrease in effect of BMI were "Uterine & Adnexa Procedures", and two other types of "Uterine & Adnexa Procedures" were also in top-10. Existing research reported relationship between obesity and complications after gynecological laparoscopic surgery as not significant [35,36]. And our subgroup model showed the decrease may be due to overweight patient. But its significance is close to threshold. We suggest further studies are needed.

**Medication related to coefficient change of BMI:** All of our experiments showed that tazobactam can increase the effect of obesity in infection. That may because obesity infection patients required higher dose of tazobactam and faced a higher risk of nephrotoxicity [37-40]. Insulin regular,human buffered was also related to effect improvement of BMI in cardiac surgery. Combination of obesity and hyperglycemia is a well-known risk factor for cardiac surgery. Rifaximin showed significantly related to decrease in coefficient of obesity. Several researches have studied effect of Rifaximin in liver disease, weight and gut microbiome, but none of these researches can directly explain this interaction.

## EFFECT OF PULSE IN HETEROGENEOUS PATIENTS

**Diseases related to coefficient change of pulse:** In meta-regression, liver disease were top-2 admission diagnoses related to effect improvement of pulse and another 2 liver diseases also in the top-10. The result can be supported by both subgroup model and subgroup analysis when pulse is >100. Generally, increasing pulse is a common symptom in severe liver disease, and cardiovascular dysfunction often occurs as the disease progresses [41]. Cerebrovascular condition is another important factor correlated with increasing effect of pulse according to meta-regression (3 of the top-10). However, results from 3 significant admission diagnoses were not supported by the subgroup model and subgroup analysis. Given that personalized models were built using similar samples from other patients with cerebrovascular condition, we further combined samples from other cerebrovascular related admissions, and found OR of pulse >100 bpm increased significantly. We found two existing studies on the relationship between heart rate and outcome of stroke, but their conclusions were inconsistent [42,43]. While other studies showed that heart rate variability is significantly related to mortality of patient with head injury [44,45]. In cardiac surgery, orthopedic surgery and infection, the relation between pulse and AKI is weak.

## EFFECT OF VANCOMYCIN IN HETEROGENEOUS PATIENTS

**Diseases related to coefficient change of vancomycin:** The coefficients of vancomycin significantly increased in gastrointestinal surgery, orthopedic surgery (exclude joint replacement) and infection according to all experiments. Skin graft was also a significant moderator in meta-regression, its significance (p=0.056) in subgroup model was close to the threshold. It mainly reflects the danger of infection in these subgroups. And in gastrointestinal surgery, significant systemic absorption may occur when intestinal mucosal integrity is compromised, and risk of nephrotoxicity may increase. In admissions for cardiac procedure, cardiac device and joint replacement, coefficients of vancomycin significantly decreased. In these subgroups, we found vancomycin was used in 44% of patients, which suggests vancomycin was possibly used to prevent infection.

**Medication related to coefficient change of vancomycin:** The use of tazobactam showed the strongest correlation with increasing coefficient of vancomycin. A recent meta-analysis study also showed the interaction between the two drugs [46]. Glucose was also significantly related to effect improvement of vancomycin in all experiment, and result of budipine hcl was supported by subgroup model. Result also showed simvastatin decreased the OR of tazobactam. Existing research has shown simvastatin to have potential antimicrobial effects [47,48].

© 2022 Liu K et al. *JAMA Network Open.*

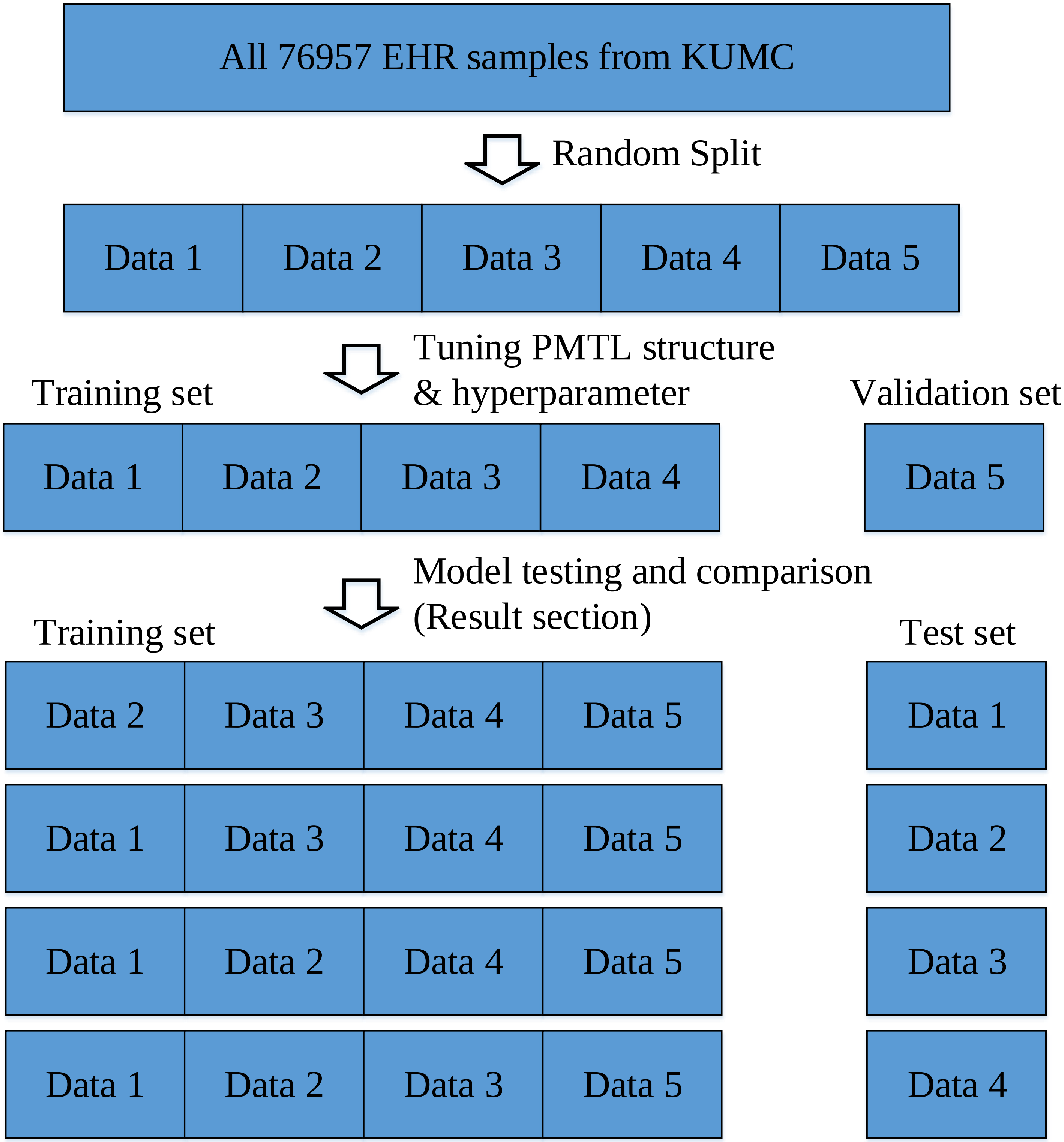


**eFigure 1.** Validation Scheme of This Study

© 2022 Liu K et al. *JAMA Network Open.*

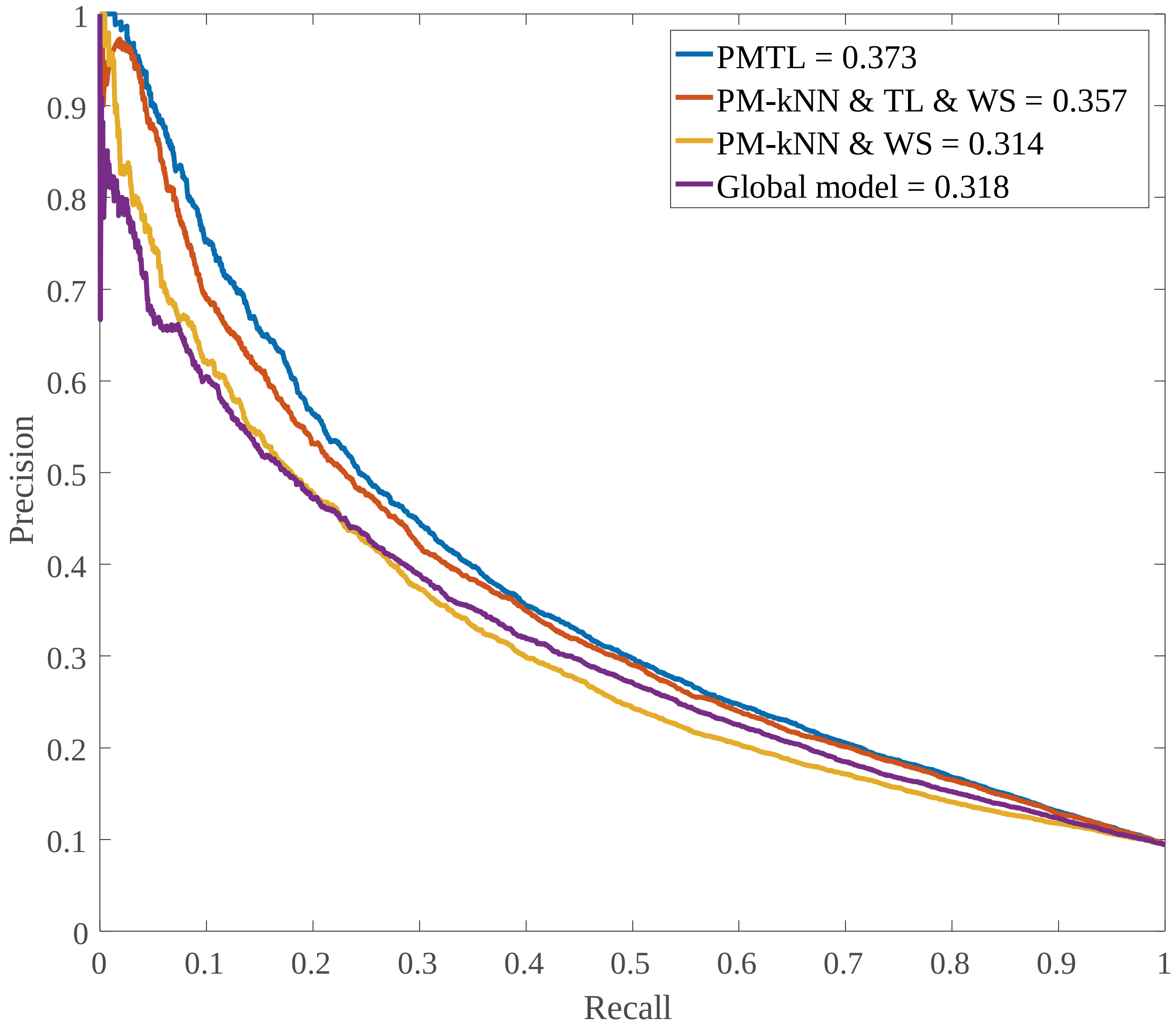


**eFigure 2.** AUPRC Comparison of Personalized Models With and Without Similarity Measure Learning in General Patients Based on 4 of 5 Data Folds Used for Testing

© 2022 Liu K et al. *JAMA Network Open.*

**a** AUROC via optimized parameters

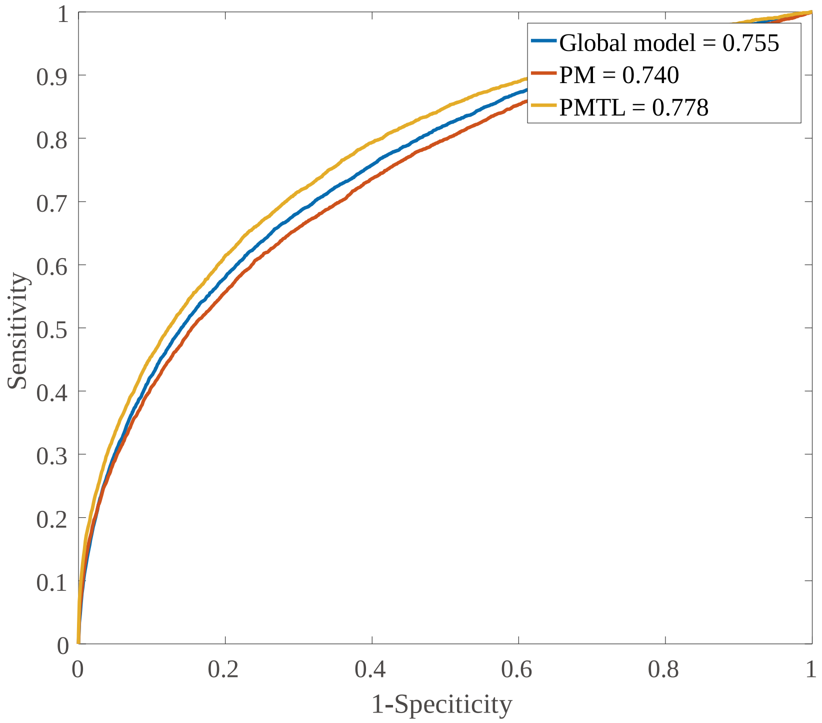


**b** AUPRC via optimized parameters

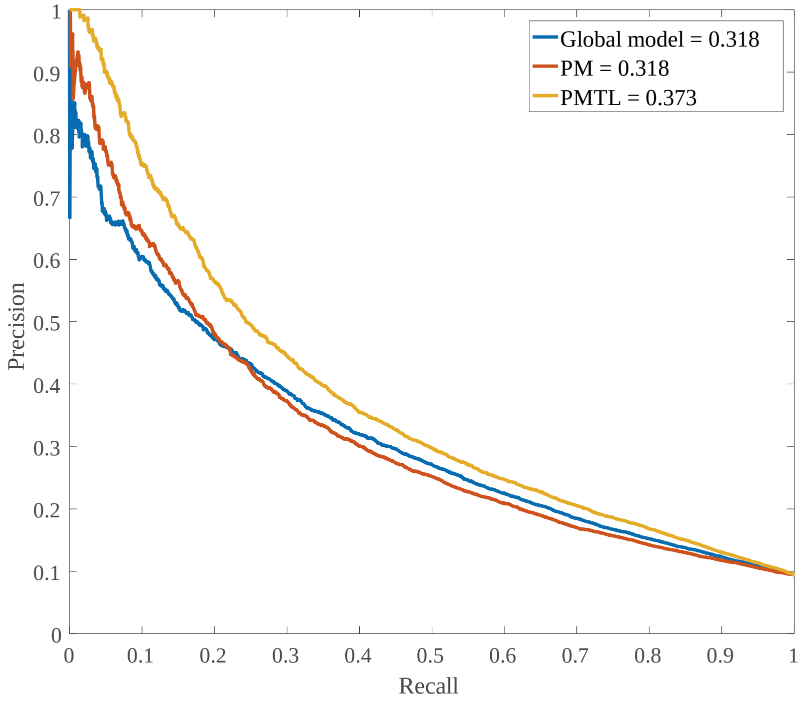


**eFigure 3.** Model Discrimination Comparison in General Inpatients

© 2022 Liu K et al. *JAMA Network Open.*

(**a**) AUROC using optimized parameters. (**b**) AUPRC using optimized parameters. Personalized models used 10% training sample as threshold for number of similar patients; global models used 100% training samples.

© 2022 Liu K et al. *JAMA Network Open.*

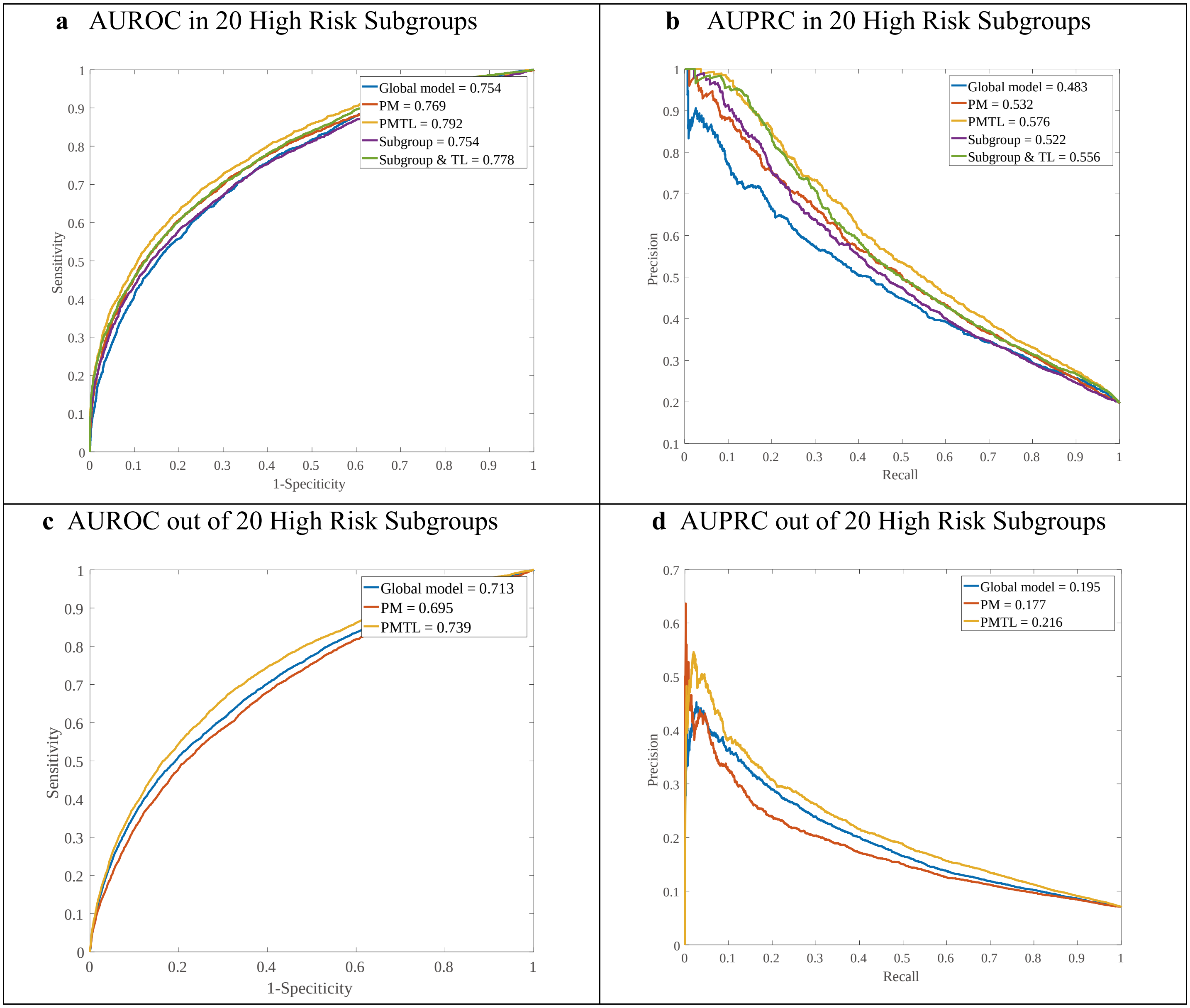


**eFigure 4.** Overall Performance of Personalized and Subgroups Modeling In and Out of Top 20 High-Risk Subgroup

(**a**) AUROC of models in 20 high risk subgroups; (**b**) AUPRC of models in 20 high risk subgroups; (**c**) AUROC of models out of 20 high risk subgroups; (**d**) AUPRC of models out of 20 high risk subgroups.

© 2022 Liu K et al. *JAMA Network Open.*

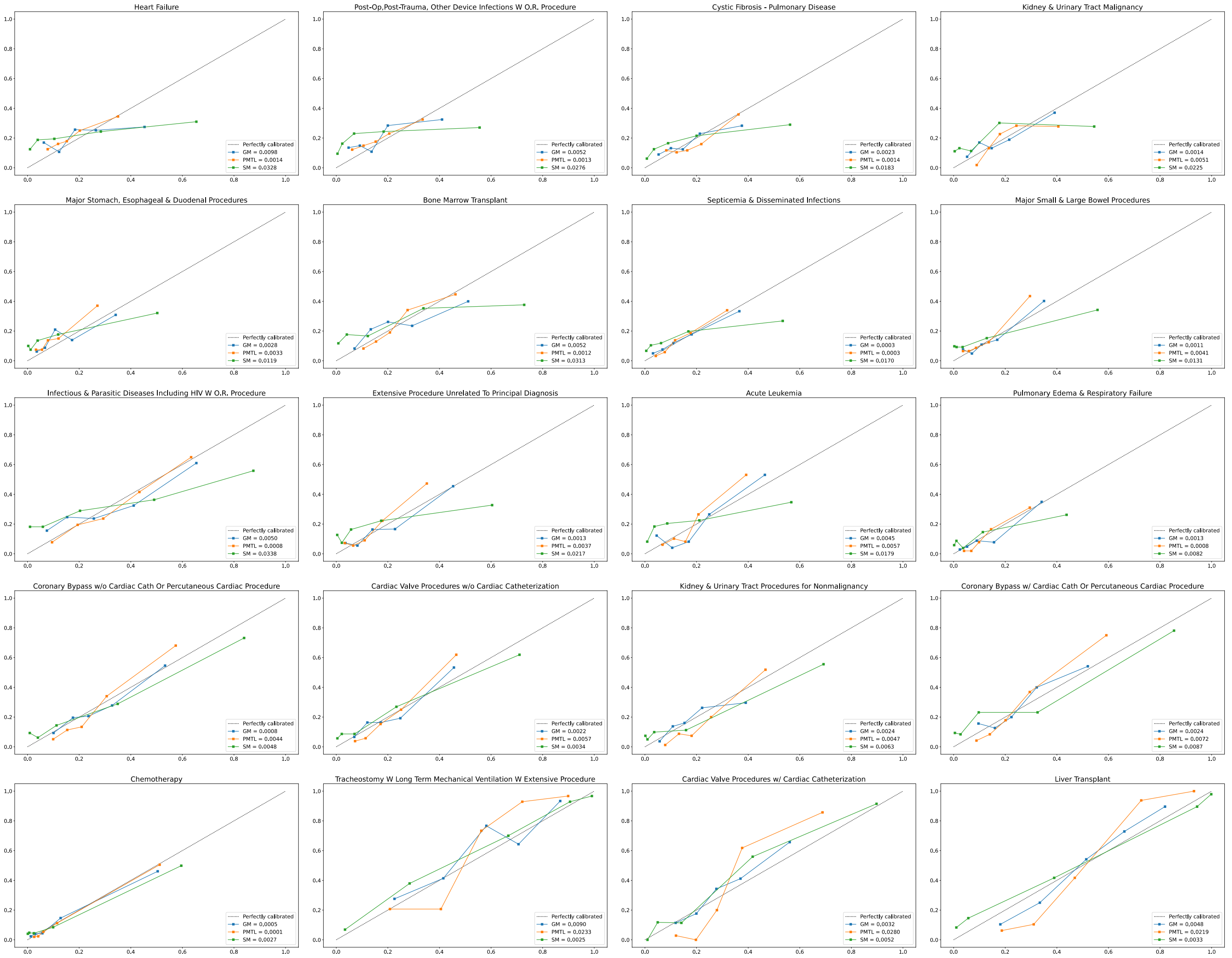


**eFigure 5.** Calibration of Global, Subgroup and Personalized Model in Each of Top 20 High-Risk Subgroup

Brier scores are shown in legend.

© 2022 Liu K et al. *JAMA Network Open.*

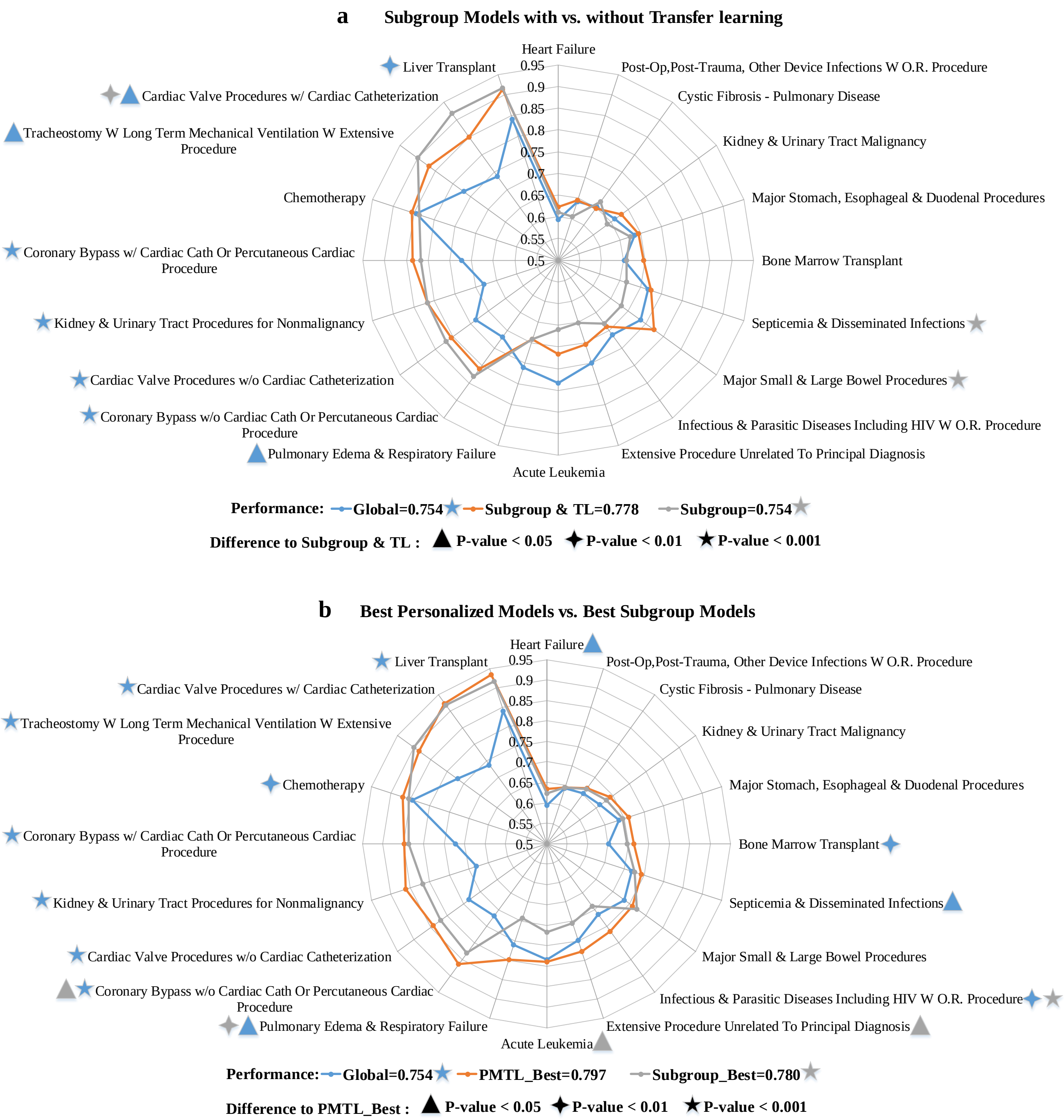


**eFigure 6.** Radar Chart of AUROC Comparison for Subgroup Models With Transfer Learning

(**a**) Comparison of subgroup modeling with and without transfer learning; (**b**) Comparison of best personalized model and best subgroup modeling, i.e. for each subgroup, best personalized and subgroup models between models with and without transfer learning are selected for evaluation.

© 2022 Liu K et al. *JAMA Network Open.*

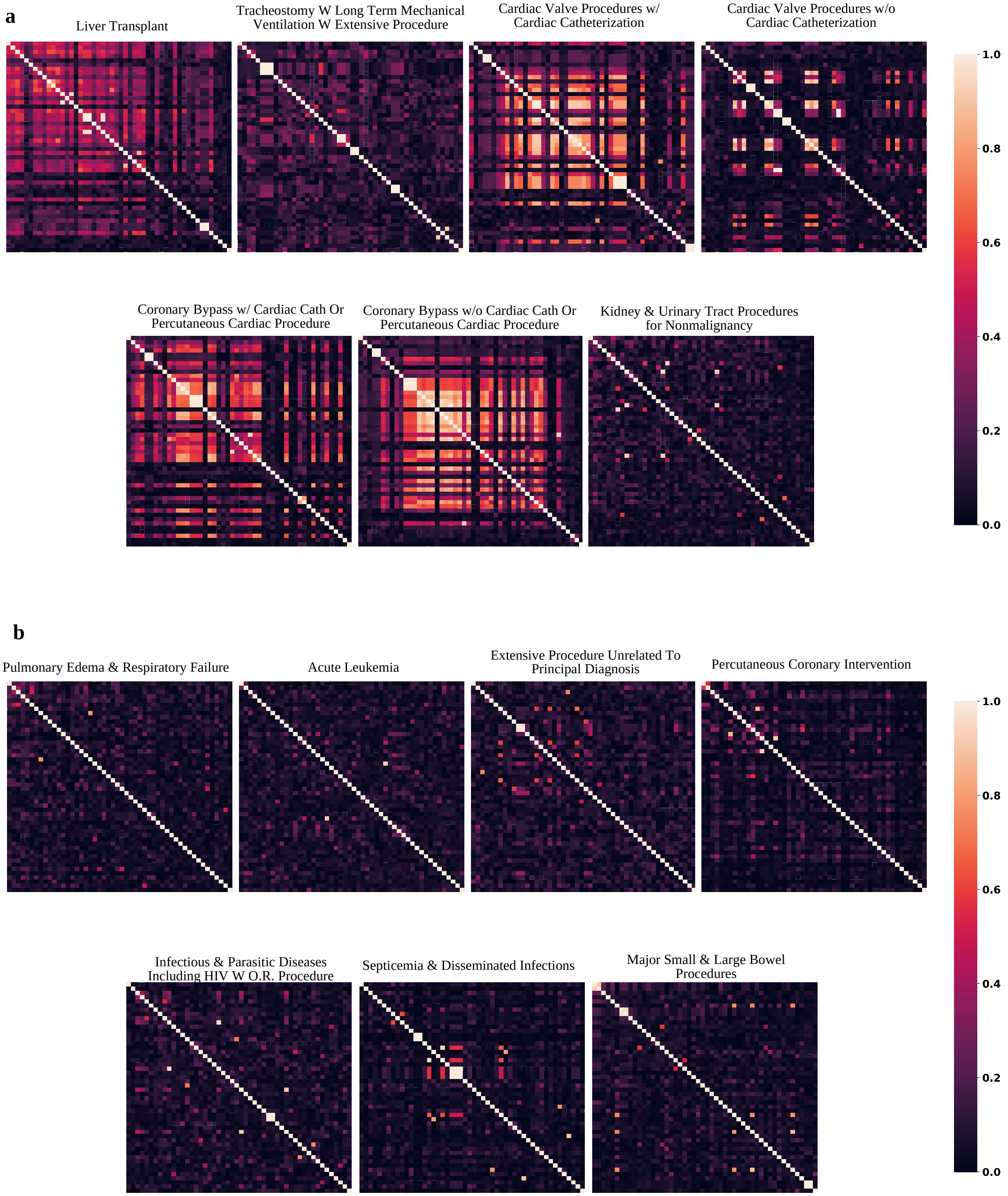


**eFigure 7.** Absolute Pearson Correlation Coefficient Among Top-50 Important Predictors in Different Subgroups

**(a)** cases in subgroups where both PMTL and subgroup models perform well, and their performance difference is insignificant. **(b)** cases in subgroups where PMTL performed much better than subgroup models.

© 2022 Liu K et al. *JAMA Network Open.*

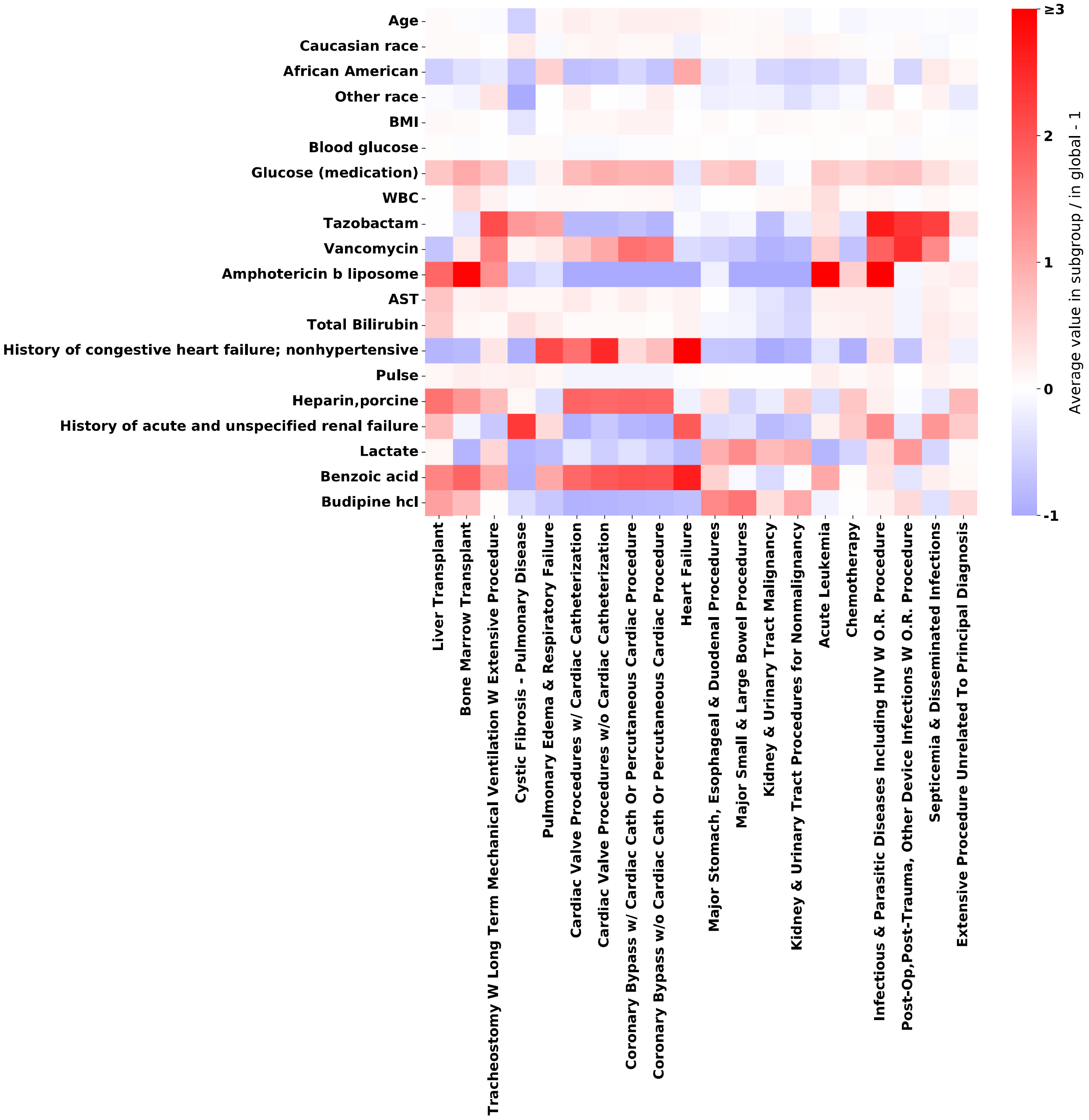


**eFigure 8.** Average Value Changes of Top-20 Predictors Determined by Global Model in Subgroups

© 2022 Liu K et al. *JAMA Network Open.*

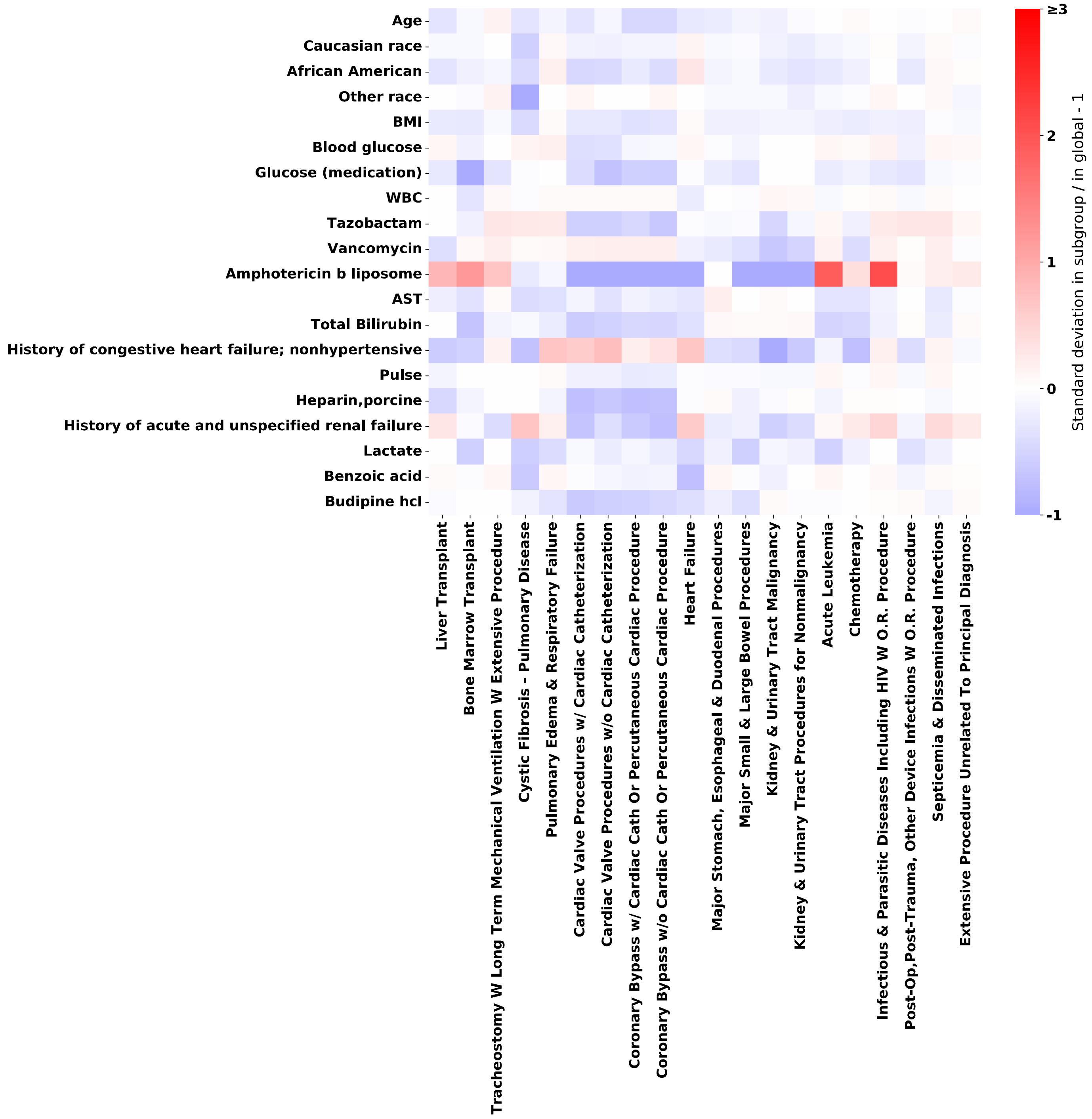


**eFigure 9.** Standard Deviation Changes of Top-20 Predictors Determined by Global Model in Subgroups

© 2022 Liu K et al. *JAMA Network Open.*

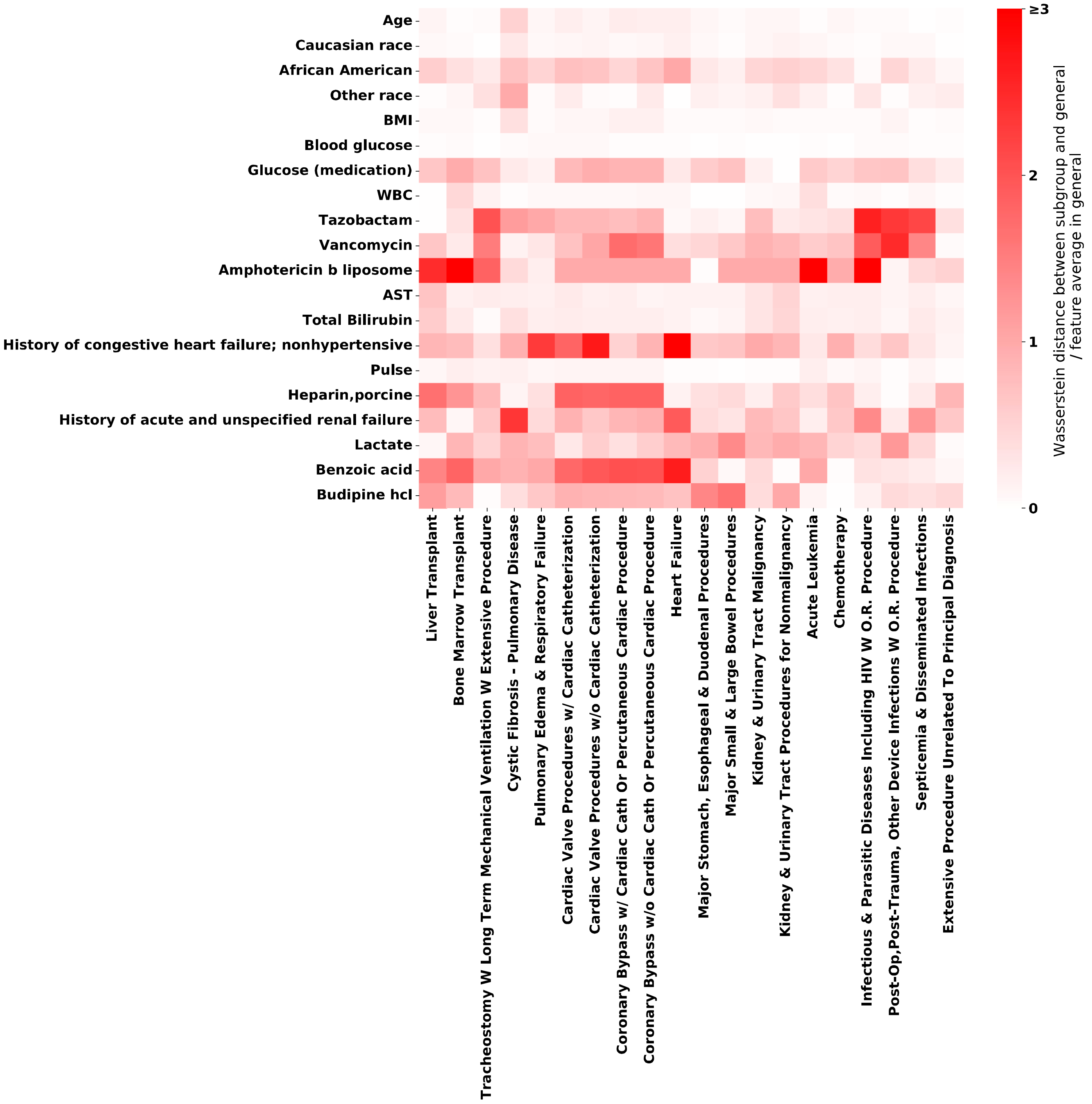


**eFigure 10.** Wasserstein Distance of Top-20 Predictor Distribution Between Patients in General and Subgroups

© 2022 Liu K et al. *JAMA Network Open.*

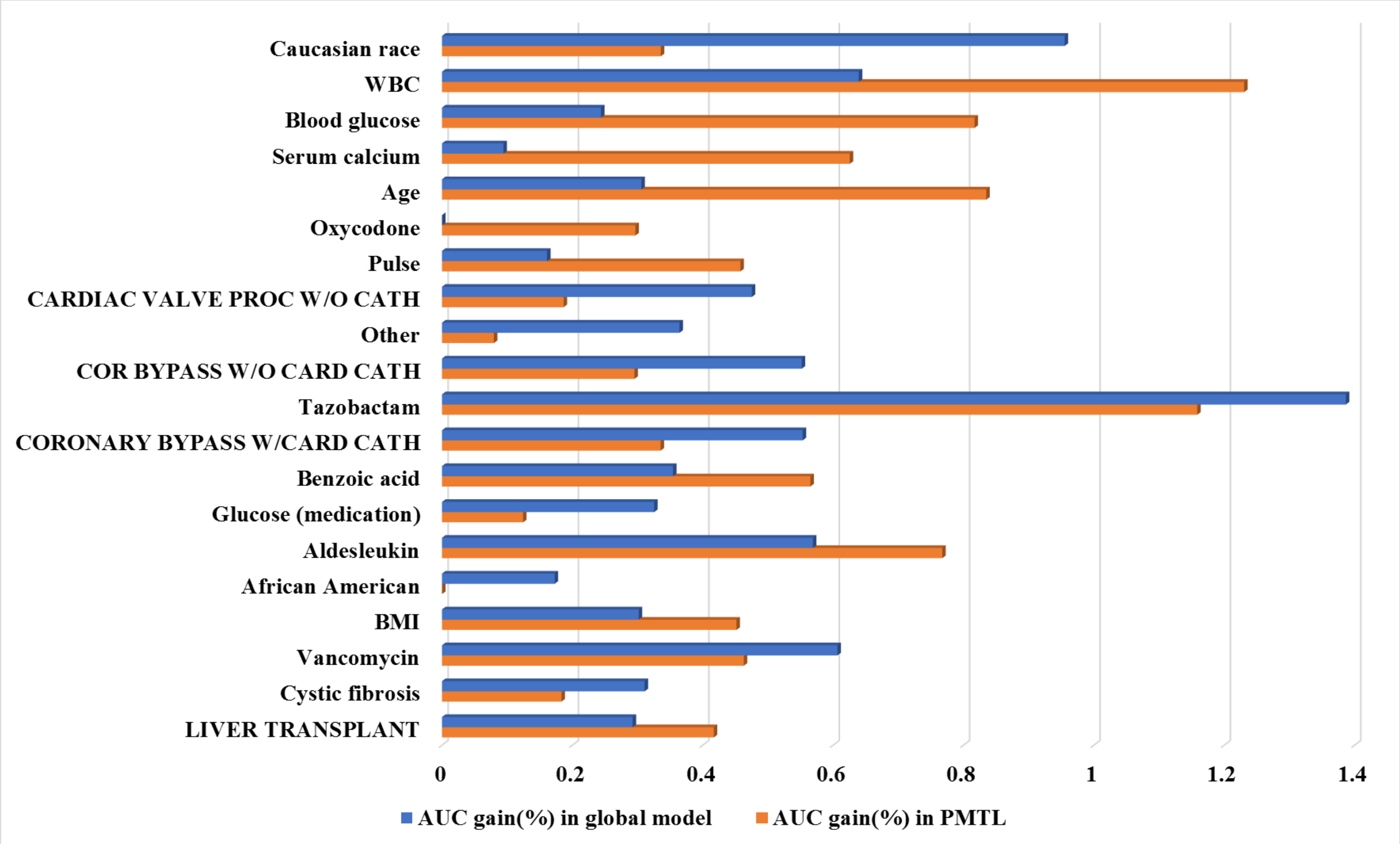


**eFigure 11.** Top 20 Predictors Where Their Effects Differed Between Global Model and PMTL

© 2022 Liu K et al. *JAMA Network Open.*

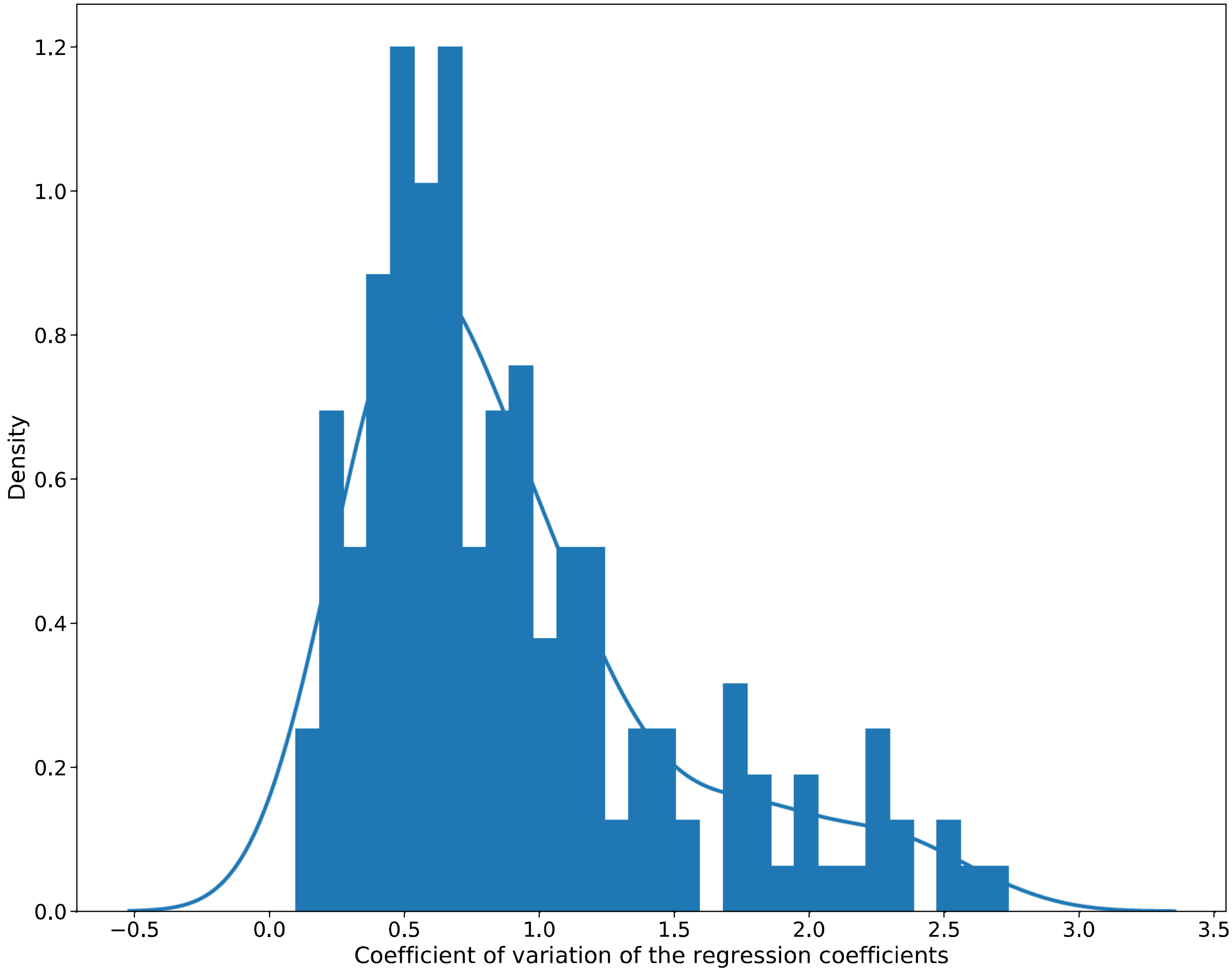


**eFigure 12.** Coefficient of Variation of the Regression Coefficients of Top-200 Features in PMTL

To avoid extreme value, we omit results of coefficient of variation higher than the result of 90% of features.

© 2022 Liu K et al. *JAMA Network Open.*

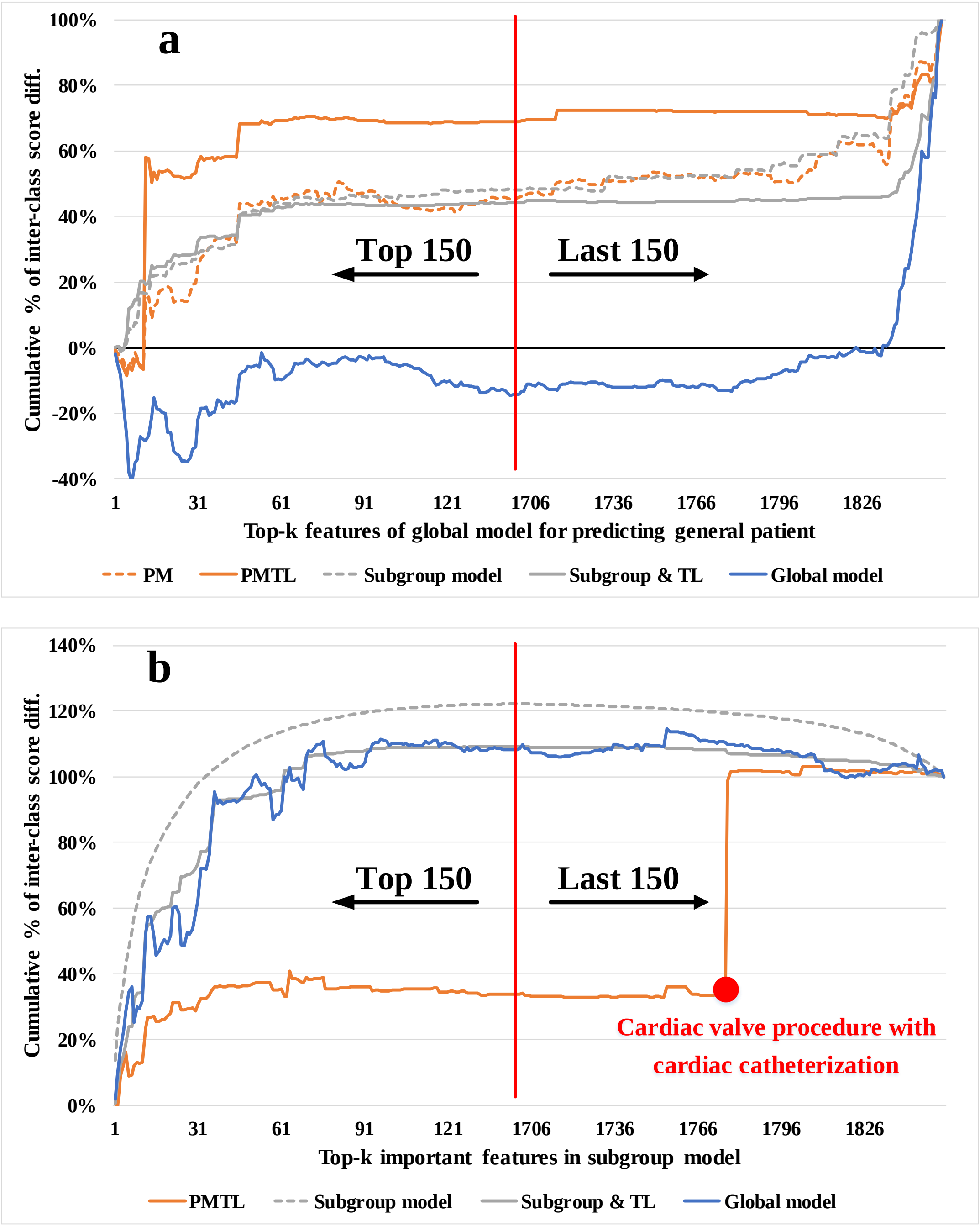


**eFigure 13.** Difference of Feature Effects Between PMTL and Other Models: A Case Study of Subgroup of Cardiac Valve Procedure With Cardiac Catheterization

Meaning of inter-class score difference is introduced in eAppendix 6. “Cumulative % of inter-class score diff.” (y-axis) is calculated by current inter-class score difference of a model/ final inter-class score different of the same model. To better visualize the impact of features at either extreme of ranking, figures are plotted against split horizontal axes. (**a**) Features are ranked by their effects in the global model when estimating general patients. We observe the most important predictors for global model are harmful to its estimation in this subgroup (negative values in y-axis), but these errors are well corrected by personalized model. (**b**) Features are ranked by their effects in the subgroup model. We can

© 2022 Liu K et al. *JAMA Network Open.*

observe effect change of "cardiac valve procedure with cardiac catheterization" (feature used to determine this subgroup) is important for estimation in PMTL, but both global and subgroup model cannot learn it.

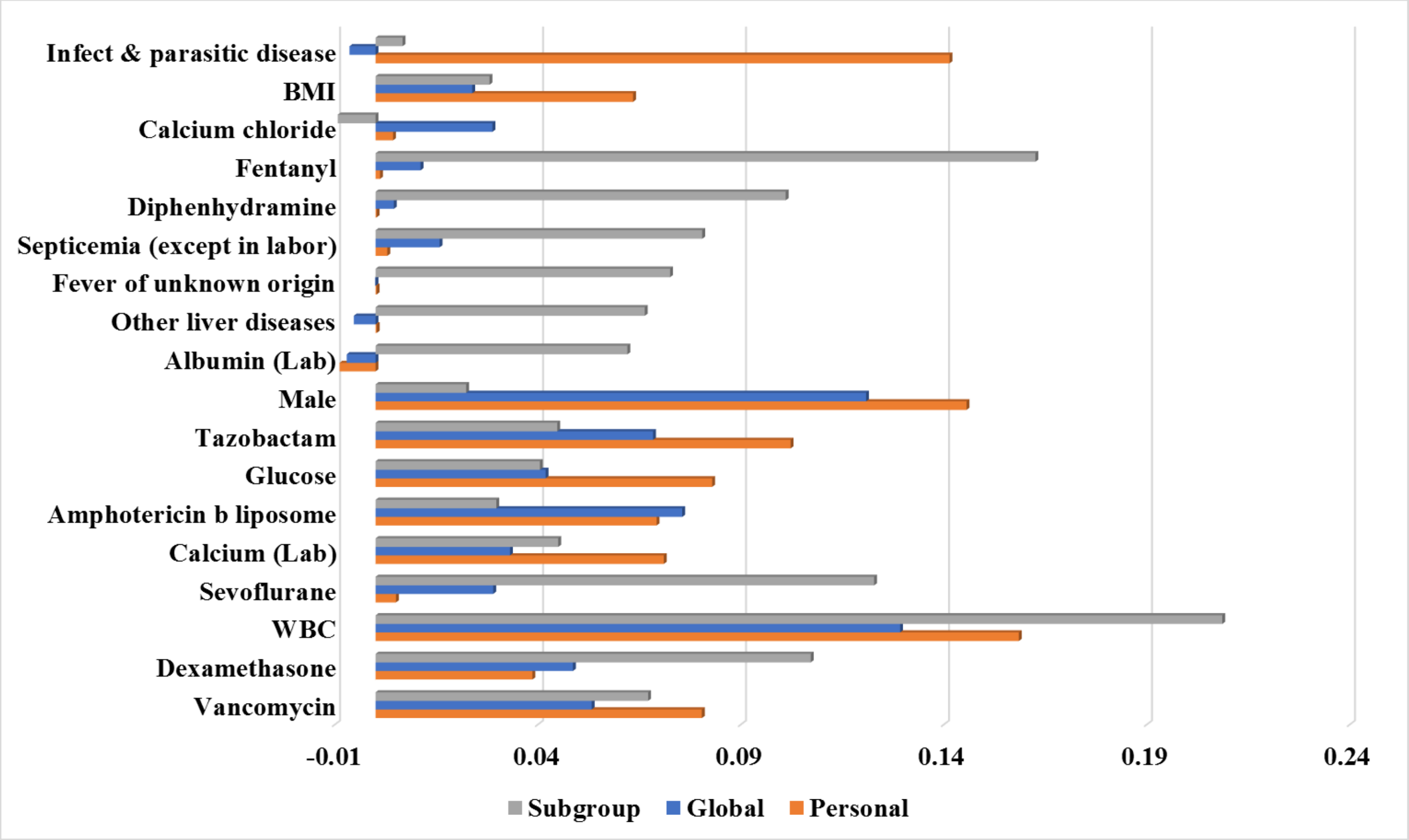


**eFigure 14.** Difference of Feature Effects Between PMTL and Other Models: A Case Study of Subgroup of Infect & Parasitic Disease

This figure shows inter-class risk score different provide by Top 10 features (union) of subgroup, global and personalized model in subgroup of infect & parasitic disease. PMTL significantly outperformance global, subgroup model in this subgroup (0.765 vs 0.713 and 0.681, p<0.01 for both comparisons). If feature of infect & parasitic disease is deleted from the PMTL, AUROC of PMTL will decrease to 0.758, but still significantly outperformance global and subgroup model (p<0.02). That means in addition to "infect & parasitic disease" itself, heterogeneities introduced by other features are also captured by PMTL.

© 2022 Liu K et al. *JAMA Network Open.*

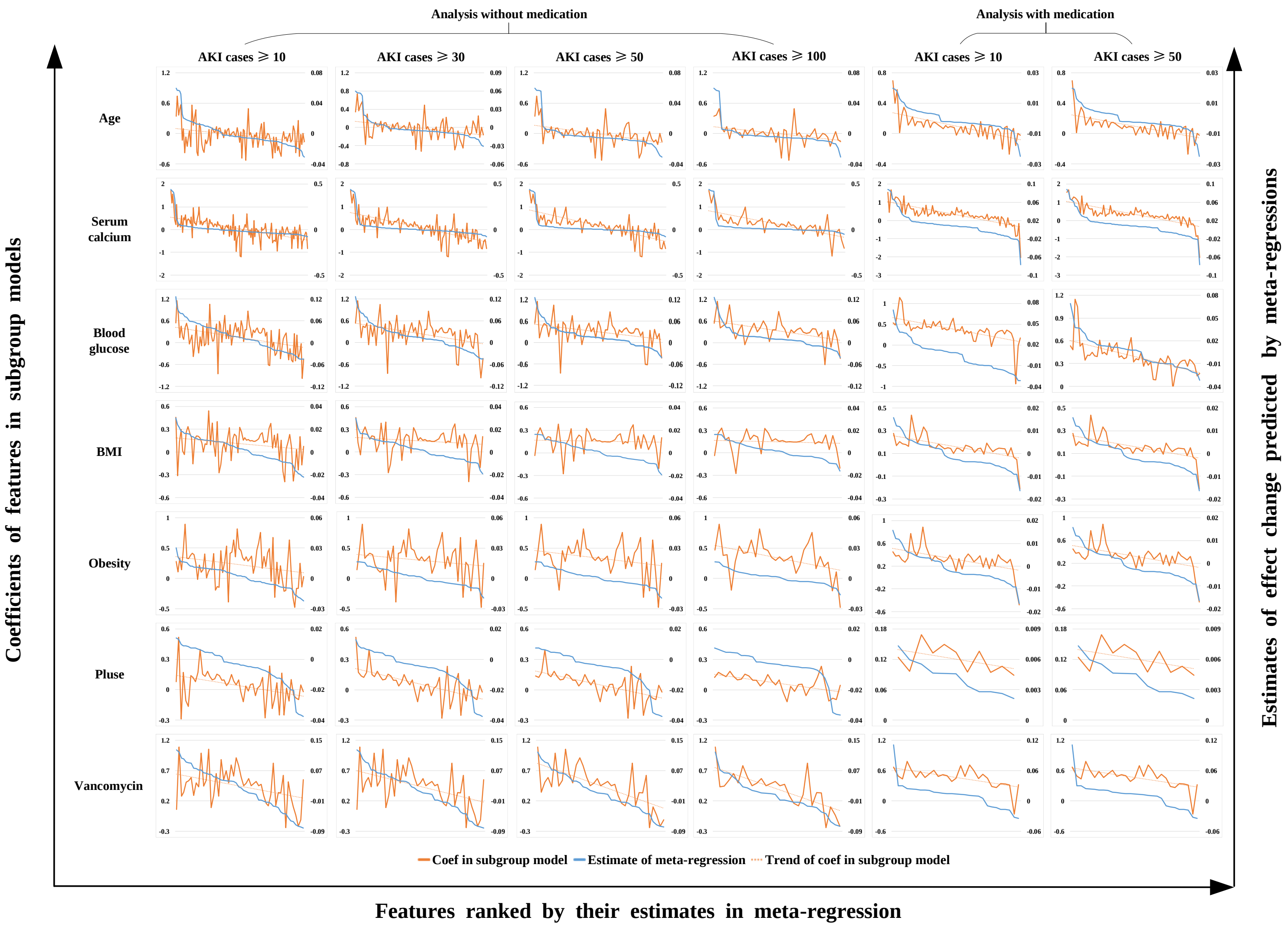


**eFigure 15.** Effect Change of Factors Estimated by Subgroup Models and Meta-Regression on PMTL

© 2022 Liu K et al. *JAMA Network Open.*

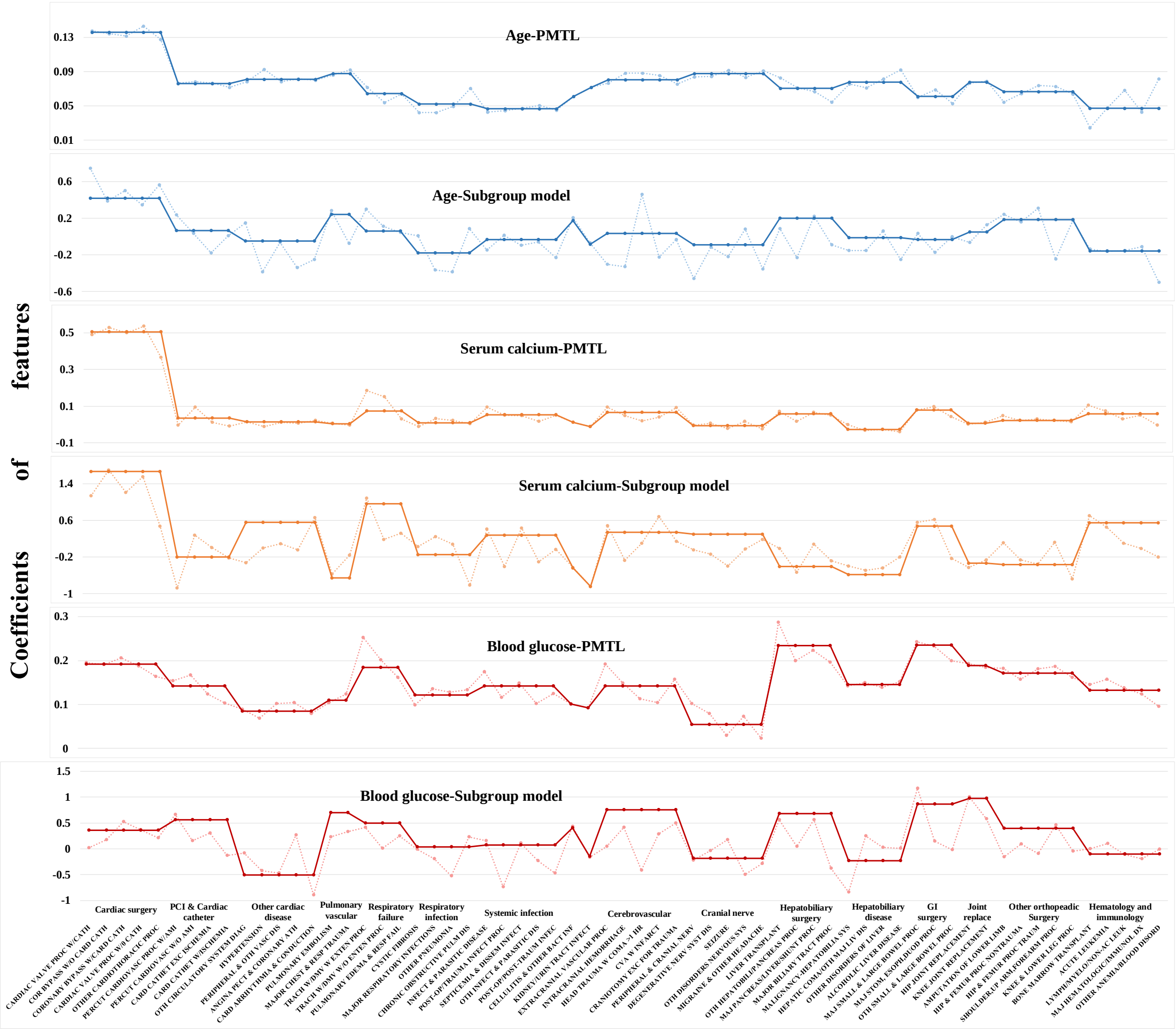


**eFigure 16.** Comparison of Coefficients Estimated by PMTL and Subgroup Models, Cases of Age, Serum Calcium and Blood Glucose

Solid and dotted lines stand for coefficients in large and small subgroups respectively. Trends of feature effect change in large subgroups were similar between PMTL and subgroup models. However, PMTL provided more stable effect estimation in granular subgroups.

© 2022 Liu K et al. *JAMA Network Open.*

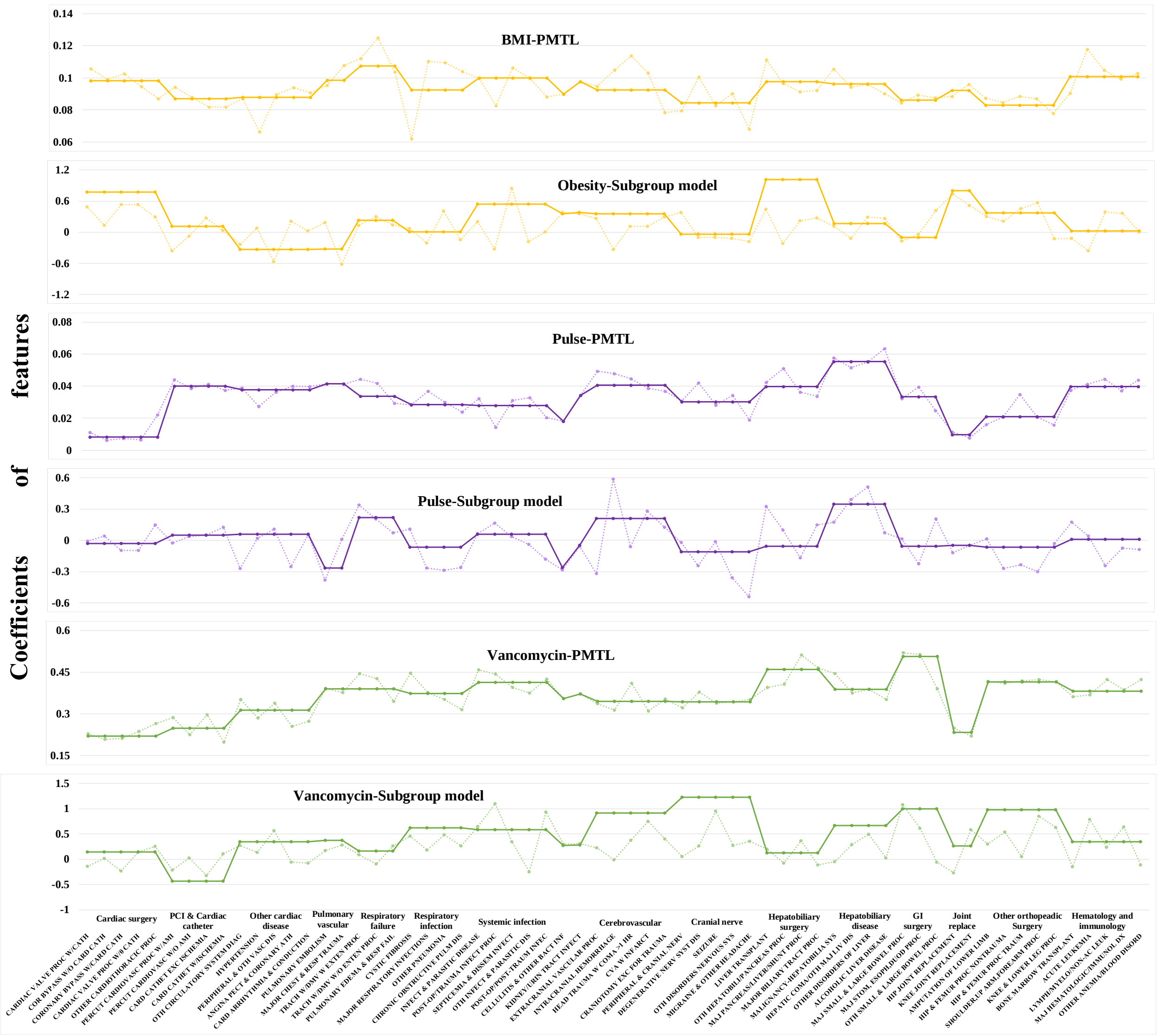


**eFigure 17.** Comparison of Coefficients Estimated by PMTL and Subgroup Models, Cases of BMI, Pulse and Vancomycin

Solid and dotted lines stand for coefficients in large and small subgroups respectively. Trends of feature effect change in large subgroups were similar between PMTL and subgroup models. However, PMTL provided more stable effect estimation in granular subgroups.

© 2022 Liu K et al. *JAMA Network Open.*

**eTable 1.** Variables Used in This Study

| Feature category | # of Variables | Detail |
|---|---|---|
| Demographics | 7 | Age, race, gender |
| Vital signs | 5 | BMI, Diastolic BP, Systolic BP, Pulse, Temperature |
| Lab test | 14 | Albumin, ALT, AST, Ammonia, Blood Bilirubin, BUN, Ca, CK-MB, CK, Glucose, Lipase, Platelets, Troponin, WBC |
| Admission diagnosis | 315 | APR-DRG from the University Health System Consortium (UHC) / Vizient |
| Inpatient Medication | 1271 | All medications are mapped to RxNorm at the ingredient level |
| Outpatient Medication | | |
| Medical History | 280 | ICD9 codes grouped into major diagnoses with Clinical Classifications Software (CCS) |
| AKI_stage | 1 | Label = 0 or 1 (AKI did not occur or occurred during encounter) |

© 2022 Liu K et al. *JAMA Network Open.*

**eTable 2.** Characteristics of Patients

| Feature | Class | |
|---|---|---|
| | Non-AKI (n = 69698) | AKI (n = 7259) |
| **Age** | | |
| 18 – 25 | 4596 (6.59%) | 357 (4.92%) |
| 26 – 35 | 7339 (10.53%) | 581 (8.00%) |
| 36 – 45 | 8601 (12.34%) | 812 (11.19%) |
| 46 – 55 | 14374 (20.62%) | 1410 (19.42%) |
| 56 – 64 | 16192 (23.23%) | 1906 (26.26%) |
| >65 | 18596 (26.68%) | 2193 (30.21%) |
| | | |
| **Male** | 37850 (54.31%) | 4309 (59.36%) |
| | | |
| **Race** | | |
| Caucasian | 53177 (76.30%) | 5408 (74.50%) |
| African American | 9336 (13.39%) | 1065 (14.67%) |
| Asian | 600 (0.86%) | 54 (0.74%) |
| Other Races (American Indian or Alaska Native, Native Hawaiian or Other Pacific Islander, Two Races, and not reported) | 6585 (9.45%) | 732 (10.09%) |
| | | |
| **Vitals** | | |
| BMI > 30 | 25347 (36.37%) | 3095 (42.64%) |
| Diastolic > 90 | 6053 (8.68%) | 570 (7.85%) |
| Systolic > 140 | 14706 (21.10%) | 1677 (23.10%) |
| | | |
| **Abnormal labs** | | |
| Serum calcium | 23140 (33.20%) | 3059 (42.14%) |
| Blood glucose | 10476 (15.03%) | 1594 (21.96%) |
| Platelets | 17352 (24.90%) | 2323 (32.00%) |
| WBC | 20189 (28.97%) | 3121 (42.99%) |
| | | |
| **Admission reasons** | | |
| Liver Transplant | 147 (0.21%) | 157 (2.16%) |
| Long Term Mechanical Ventilation | 130 (0.19%) | 142 (1.96%) |
| Cardiac Valve Procedures | 630 (0.90%) | 206 (2.84%) |
| Coronary Bypass | 867 (1.24%) | 325 (4.48%) |
| Chemotherapy | 1544 (2.22%) | 267 (3.68%) |
| Septicemia & Disseminated Infections | 2420 (3.47%) | 425 (5.85%) |
| | | |
| **Medications** | | |
| Vancomycin | 14266 (20.47%) | 2403 (33.10%) |
| Tazobactam | 11385 (16.33%) | 2276 (31.35%) |
| Aldesleukin | 107 (0.15%) | 154 (2.12%) |

© 2022 Liu K et al. *JAMA Network Open.*

**eTable 3.** AUROC of Different Models in Validation Set

Risk ranking model calculates patient similarity based on difference in estimated AKI risk, i.e., estimated risk in the global logistic regression model. For PM-kNN, similar sample are matched based on k-Nearest Neighbor (similarity measure is based on features' coefficient in global logistic regression); for PM-Kmeans, similar sample are matched based on K-means. WS, weighted sample; SL, similarity measure learning same as PMTL; TL, transfer learning. PMTL, proposed personalized model with transfer learning (same as PM-kNN & TL & SL & WS).

| **Models** | **Sample Size or num. of groups** | | | | | |
|---|---|---|---|---|---|---|
| | **0.05 or 20** | **0.1 or 10** | **0.2 or 5** | **0.05 or 20** | **0.1 or 10** | **0.2 or 5** |
| | **AUROC** | | | **AUPRC** | | |
| Global model | 0.655 | 0.684 | 0.713 | 0.173 | 0.206 | 0.24 |
| Risk-ranking | 0.724 | 0.708 | 0.698 | 0.242 | 0.262 | 0.291 |
| PM-Kmeans | 0.718 | 0.721 | 0.732 | 0.253 | 0.267 | 0.277 |
| PM-Kmeans & TL | 0.76 | 0.759 | 0.756 | 0.325 | 0.334 | 0.331 |
| PM-kNN | 0.714 | 0.732 | 0.752 | 0.278 | 0.294 | 0.302 |
| PM-kNN & WS | 0.717 | 0.738 | 0.759 | 0.289 | 0.307 | 0.319 |
| PM-kNN & TL | 0.765 | 0.769 | 0.772 | 0.348 | 0.347 | 0.341 |
| PM-kNN & TL & WS | 0.767 | 0.771 | 0.775 | 0.352 | 0.352 | 0.35 |
| PM-kNN & WS & SL | 0.718 | 0.744 | 0.758 | 0.299 | 0.319 | 0.337 |
| **PMTL** | 0.776 | **0.779** | 0.779 | 0.371 | **0.374** | 0.371 |

© 2022 Liu K et al. *JAMA Network Open.*

**eTable 4.** AUROC of Personalized Model Among Similar Samples in Validation Set

AUROCs presented in this table are averages of models' performance intra groups. Averages are weighted by sample size. Note that average AUC of the Risk-ranking models for samples within each group is only about 0.5. That means Risk-ranking models cannot distinguish risk difference of patients in the same group, and its performance depends on the global model. As a comparison, global model performance within each random group is consistent with its performances in all patients, because samples in random groups have a similar distribution to the overall samples. Transfer learning can further improve the discrimination on similar samples, illustrating the effectiveness of transfer learning.

| Models | Num. of groups | | |
|---|---|---|---|
| | 5 groups | 10 groups | 20 groups |
| Risk-ranking | 0.511 | 0.503 | 0.502 |
| Global model | 0.708 | 0.68 | 0.648 |
| Global model & TL | 0.743 | 0.735 | 0.724 |
| PM-Kmeans | 0.727 | 0.707 | 0.684 |
| PM-Kmeans & TL | 0.752 | 0.744 | 0.73 |

© 2022 Liu K et al. *JAMA Network Open.*

**eTable 5.** Performance of Similarity Measure Learning in Test Set

We can see models with similarity measure learning always have higher performance. WS, weighted sample; TL, transfer learning. For PM-kNN, similar sample are matched based on k-Nearest Neighbor (similarity measure is based on features' coefficient in global logistic regression).

| Measure | Population | Basic model | With similarity measure learning | Without similarity measure learning | P value |
|---|---|---|---|---|---|
| AUROC | General patients | PM-kNN & WS & TL | 0.778 | 0.773 | 0.0001 |
| | | PM-kNN & WS | 0.740 | 0.738 | 0.57 |
| | Top-20 subgroups | PM-kNN & WS & TL | 0.792 | 0.783 | 0.0001 |
| | | PM-kNN & WS | 0.769 | 0.761 | 0.057 |
| AUPRC | General patients | PM-kNN & WS & TL | 0.373 | 0.358 | <0.0001 |
| | | PM-kNN & WS | 0.318 | 0.314 | 0.31 |
| | Top-20 subgroups | PM-kNN & WS & TL | 0.576 | 0.553 | <0.0001 |
| | | PM-kNN & WS | 0.532 | 0.521 | 0.081 |

© 2022 Liu K et al. *JAMA Network Open.*

**eTable 6.** Performance of Sample Weighting in Test Set

We can see models with sample weighting always have higher performance. TL, transfer learning. For PM-kNN, similar sample are matched based on k-Nearest Neighbor (similarity measure is based on features' coefficient in global logistic regression).

| **Measure** | **Population** | **Based model** | **With sample weighting** | **Without sample weighting** | **P value** |
|---|---|---|---|---|---|
| AUROC | General patients | PM-kNN & TL | 0.773 | 0.773 | 0.22 |
| | | PM-kNN | 0.738 | 0.731 | <0.0001 |
| | Top-20 subgroups | PM-kNN & TL | 0.783 | 0.782 | 0.36 |
| | | PM-kNN | 0.761 | 0.750 | <0.0001 |
| AUPRC | General patients | PM-kNN & TL | 0.358 | 0.356 | 0.12 |
| | | PM-kNN | 0.314 | 0.302 | <0.0001 |
| | Top-20 subgroups | PM-kNN & TL | 0.553 | 0.549 | 0.046 |
| | | PM-kNN | 0.521 | 0.501 | <0.0001 |

© 2022 Liu K et al. *JAMA Network Open.*

**eTable 7.** Performance (AUROC) of Feature Selection in Dealing With Overfitting in Personalized Modeling

Feature selection is performed after similar sample matching. For feature selection with chi-square test and logistic regression, top 200 ranked features are selected according to our previous AKI study[49]; for PCA, we referred to the general practice and set the variance needed to be explained as >95%. When combining transfer learning with PCA, we performed transfer learning first and then PCA, while combining transfer learning with chi-square test and logistic regression, we performed feature selection before transfer learning. TL, transfer learning; chi2, chi-square test; PCA, principal component analysis; LR, logistic regression

| **Class** | **Models** | **Sample size or num. of groups** | | |
|---|---|---|---|---|
| | | **20% or 5 groups** | **10% or 10 groups** | **5% or 20 groups** |
| **Feature Selection Only** | PM-Kmeans | 0.732 | 0.721 | 0.718 |
| | PM-Kmeans & chi2 | 0.735 | 0.732 | 0.724 |
| | PM-Kmeans & PCA | 0.722 | 0.718 | 0.71 |
| | PM-Kmeans & LR | 0.702 | 0.71 | 0.712 |
| | PM-kNN | 0.752 | 0.732 | 0.714 |
| | PM-kNN & chi2 | 0.752 | 0.733 | 0.716 |
| | PM-kNN & PCA | 0.742 | 0.728 | 0.712 |
| | PM-kNN & LR | 0.74 | 0.73 | 0.721 |
| **Feature Selection + Transfer Learning** | PM-Kmeans & TL | 0.756 | 0.759 | 0.76 |
| | PM-Kmeans & chi2 & TL | 0.742 | 0.747 | 0.747 |
| | PM-Kmeans & PCA & TL | 0.755 | 0.758 | 0.758 |
| | PM-Kmeans & LR & TL | 0.716 | 0.73 | 0.741 |
| | PM-kNN & TL | **0.772** | **0.769** | **0.765** |
| | PM-kNN & chi2 & TL | 0.763 | 0.759 | 0.76 |
| | PM-kNN & PCA & TL | 0.771 | 0.768 | 0.765 |
| | PM-kNN & LR & TL | 0.755 | 0.759 | 0.763 |

**eTable 8.** List of APR-DRG Selected as High-Risk Subgroups

| **Abbreviations** | **Presented in RESULT (AKI cases ≥ 50)** | **APR-DRG** |
|---|---|---|
| LIVER TRANSPLANT | √ | Liver Transplant |
| BONE MARROW TRANSPLANT | √ | Bone Marrow Transplant |
| TRACH W/DMV W EXTEN PROC | √ | Tracheostomy W Long Term Mechanical Ventilation W Extensive Procedure |
| TRACH W/DMV W/O ENTEN PROC | | Tracheostomy W Long Term Mechanical Ventilation W/O Extensive Procedure |
| RESPIRATORY SYSTEM DIAG | | Respiratory System Diagnosis w/ Ventilator Support 96+ Hours |
| CYSTIC FIBROSIS | √ | Cystic Fibrosis - Pulmonary Disease |
| PULMONARY EDEMA & RESP FAIL | √ | Pulmonary Edema & Respiratory Failure |
| MAJOR RESPIRATORY INFECTIONS | | Major Respiratory Infections & Inflammations |
| CARDIAC VALVE PROC W/CATH | √ | Cardiac Valve Procedures w/ Cardiac Catheterization |
| CARDIAC VALVE PROC W/O CATH | √ | Cardiac Valve Procedures w/o Cardiac Catheterization |
| CORONARY BYPASS W/CARD CATH | √ | Coronary Bypass w/ Cardiac Cath Or Percutaneous Cardiac Procedure |
| COR BYPASS W/O CARD CATH | √ | Coronary Bypass w/o Cardiac Cath Or Percutaneous Cardiac Procedure |
| OTHER CARDIOTHORACIC PROC | | Other Cardiothoracic Procedures |
| MAJ THORACIC & ABDOMEN PROC | | Major Thoracic & Abdominal Vascular Procedures |
| HEART FAILURE | √ | Heart Failure |
| OTH CIRCULATORY SYSTEM DIAG | | Other Circulatory System Diagnoses |
| MAJ STOM, ESOPH, DUOD PROC | √ | Major Stomach, Esophageal & Duodenal Procedures |
| MAJ SMALL & LARGE BOWEL PROC | √ | Major Small & Large Bowel Procedures |
| AMPUTATION OF LOWER LIMB | | Amputation of Lower Limb Except Toes |
| OTHER SKIN & SUBCUT PROC | | Other Skin, Subcutaneous Tissue & Related Procedures |
| MAJOR BLADDER PROC | | Major Bladder Procedures |
| KIDNEY/URINARY TRACT MALIG | √ | Kidney & Urinary Tract Malignancy |
| KIDNEY/URIN TRACT-NONMALIG | √ | Kidney & Urinary Tract Procedures for Nonmalignancy |
| ACUTE LEUKEMIA | √ | Acute Leukemia |
| CHEMOTHERAPY | √ | Chemotherapy |
| INFECT & PARASITIC DISEASE | √ | Infectious & Parasitic Diseases Including HIV W O.R. Procedure |
| POST-OP/TRAUMA INFECT PROC | √ | Post-Op,Post-Trauma, Other Device Infections W O.R. Procedure |
| SEPTICEMIA & DISSEM INFECT | √ | Septicemia & Disseminated Infections |
| FULL THICK BURNS W GRAFT | | Full Thickness Burns W Skin Graft |
| HIV W MULTIPLE MAJOR HIV | | HIV W Multiple Major HIV Related Conditions |
| EXTENSIVE PROC UNREL PDX | √ | Extensive Procedure Unrelated To Principal Diagnosis |

© 2022 Liu K et al. *JAMA Network Open.*

**eTable 9.** AUROC of Models in 31 High Risk DRG Subgroups With At Least 20 AKI Patients

*, **, ***means models' performances are different to PMTL's in significance of P<0.1, P<0.01, P<0.001 respectively. WS, weighted sample; TL, transfer learning. PMTL, personalized model with transfer learning (proposed model). For PM-kNN, similar sample are matched based on k-Nearest Neighbor (similarity measure is based on features' coefficient in global logistic regression). The subgroups presented in Result section are those with at least 50 AKI patients. Full names of the APR-DRG for all subgroups are in eTable 8.

| Drg | % of AKI | Num of AKI | PMTL | Global model | PM | PM-kNN & TL & WS | PM-kNN & WS | PM-kNN & TL | PM-kNN | Subgroup model & TL | Subgroup model |
|---|---|---|---|---|---|---|---|---|---|---|---|
| LIVER TRANSPLANT | 51.6% | 121 | 0.935 | 0.841*** | 0.915* | 0.917* | 0.879** | 0.905*** | 0.862*** | 0.914 | 0.917 |
| BONE MARROW TRANSPLANT | 23.7% | 101 | 0.713 | 0.652** | 0.700 | 0.705 | 0.666* | 0.686* | 0.652* | 0.697 | 0.658 |
| TRACH W/DMV W EXTEN PROC | 58.3% | 89 | 0.885 | 0.769*** | 0.770*** | 0.879 | 0.809* | 0.869 | 0.804* | 0.868 | 0.900 |
| TRACH W/DMV W/O ENTEN PROC | 37.5% | 23 | 0.649 | 0.690 | 0.688 | 0.663 | 0.668 | 0.672 | 0.678 | 0.788* | 0.837* |
| RESPIRATORY SYSTEM DIAG | 46.6% | 26 | 0.573 | 0.518 | 0.544 | 0.519 | 0.578 | 0.505 | 0.558 | 0.657 | 0.632 |
| CYSTIC FIBROSIS | 17.3% | 124 | 0.666 | 0.652 | 0.668 | 0.670 | 0.677 | 0.665 | 0.664 | 0.648 | 0.666 |
| PULMONARY EDEMA & RESP FAIL | 12.6% | 61 | 0.798 | 0.760* | 0.771 | 0.785 | 0.723* | 0.776 | 0.705** | 0.692*** | 0.692** |
| MAJOR RESPIRATORY INFECTIONS | 13.1% | 31 | 0.712 | 0.608** | 0.601* | 0.696 | 0.728 | 0.683 | 0.725 | 0.563* | 0.518** |
| CARDIAC VALVE PROC W/CATH | 33.2% | 59 | 0.924 | 0.738*** | 0.910 | 0.854*** | 0.867* | 0.852*** | 0.833** | 0.850** | 0.918 |
| CARDIAC VALVE PROC W/O CATH | 21.9% | 117 | 0.829 | 0.734*** | 0.842 | 0.813 | 0.816 | 0.807* | 0.790* | 0.805* | 0.819 |
| CORONARY BYPASS W/CARD CATH | 27.2% | 136 | 0.848 | 0.722*** | 0.821* | 0.821** | 0.799** | 0.819** | 0.782*** | 0.836 | 0.817* |
| COR BYPASS W/O CARD CATH | 27.4% | 128 | 0.827 | 0.719*** | 0.865** | 0.796*** | 0.800 | 0.797*** | 0.783* | 0.810 | 0.831 |
| OTHER CARDIOTHORACIC PROC | 17.2% | 26 | 0.767 | 0.761 | 0.759 | 0.735 | 0.694 | 0.736 | 0.679 | 0.703 | 0.764 |
| MAJ THORACIC & ABDOMEN PROC | 18.2% | 32 | 0.787 | 0.832 | 0.823 | 0.793 | 0.788 | 0.803 | 0.787 | 0.799 | 0.753 |
| HEART FAILURE | 21.5% | 119 | 0.634 | 0.593* | 0.607 | 0.619 | 0.585* | 0.612 | 0.569* | 0.623 | 0.610 |
| OTH CIRCULATORY SYSTEM DIAG | 14.2% | 35 | 0.658 | 0.568* | 0.564* | 0.618 | 0.55* | 0.611* | 0.530** | 0.581* | 0.527 |
| MAJ STOM, ESOPH,DUOD PROC | 14.7% | 65 | 0.711 | 0.686 | 0.661* | 0.688* | 0.641* | 0.679* | 0.639* | 0.695 | 0.675 |
| MAJ SMALL & LARGE BOWEL PROC | 15.0% | 143 | 0.76 | 0.735 | 0.732* | 0.769 | 0.728* | 0.767 | 0.722* | 0.773 | 0.679*** |
| AMPUTATION OF LOWER LIMB | 15.1% | 29 | 0.676 | 0.721 | 0.667 | 0.691 | 0.695 | 0.694 | 0.695 | 0.676 | 0.584 |
| OTHER SKIN & SUBCUT PROC | 14.8% | 26 | 0.717 | 0.780* | 0.664 | 0.733 | 0.729 | 0.743 | 0.734 | 0.677 | 0.774 |
| MAJOR BLADDER PROC | 14.8% | 34 | 0.775 | 0.731 | 0.722 | 0.709* | 0.726 | 0.713* | 0.709 | 0.678* | 0.67* |
| KIDNEY/URINARY TRACT MALIG | 18.5% | 50 | 0.692 | 0.661 | 0.662 | 0.675 | 0.670 | 0.680 | 0.656 | 0.680 | 0.640 |
| KIDNEY/URIN TRACT-NONMALIG | 18.9% | 72 | 0.835 | 0.680* | 0.862* | 0.832 | 0.854 | 0.827 | 0.835 | 0.816 | 0.817 |
| ACUTE LEUKEMIA | 20.7% | 51 | 0.790 | 0.784 | 0.724* | 0.770 | 0.714* | 0.776 | 0.711* | 0.717* | 0.660** |

© 2022 Liu K et al. *JAMA Network Open.*

| **Drg** | **% of AKI** | **Num of AKI** | **PMTL** | **Global model** | **PM** | **PM-kNN & TL & WS** | **PM-kNN & WS** | **PM-kNN & TL** | **PM-kNN** | **Subgroup model & TL** | **Subgroup model** |
|---|---|---|---|---|---|---|---|---|---|---|---|
| CHEMOTHERAPY | 14.7% | 211 | 0.869 | 0.844** | 0.855 | 0.858* | 0.839** | 0.858* | 0.830*** | 0.855 | 0.836* |
| INFECT & PARASITIC DISEASE | 31.3% | 121 | 0.765 | 0.713** | 0.724* | 0.742* | 0.701** | 0.741* | 0.692** | 0.689*** | 0.681** |
| POST-OP/TRAUMA INFECT PROC | 18.4% | 74 | 0.645 | 0.642 | 0.592 | 0.686* | 0.632 | 0.678 | 0.618 | 0.645 | 0.606 |
| SEPTICEMIA & DISSEM INFECT | 14.9% | 349 | 0.744 | 0.718* | 0.689*** | 0.745 | 0.699*** | 0.744 | 0.689*** | 0.726* | 0.666*** |
| FULL THICK BURNS W GRAFT | 14.0% | 43 | 0.788 | 0.781 | 0.712* | 0.764 | 0.705* | 0.763 | 0.710* | 0.748 | 0.705* |
| HIV W MULTIPLE MAJOR HIV | 27.7% | 20 | 0.586 | 0.520 | 0.535 | 0.575 | 0.571 | 0.565 | 0.532 | 0.538 | 0.681 |
| EXTENSIVE PROC UNREL PDX | 19.2% | 50 | 0.778 | 0.749 | 0.721* | 0.754 | 0.725 | 0.756 | 0.716* | 0.705* | 0.652** |
| Above 31 subgroups | 19.4% | - | 0.783 | 0.750*** | 0.759*** | 0.775*** | 0.753*** | 0.775*** | 0.742*** | 0.769*** | 0.744*** |
| Remaining | 6.8% | - | 0.732 | 0.704*** | 0.687*** | 0.726*** | 0.687*** | 0.725** | 0.681*** | - | - |
| Num. of Drgs where PMTL outperform | - | - | | 27 (87.10%) | 25 (80.65%) | 23 (74.19%) | 23 (74.19%) | 24 (77.42%) | 25 (80.65%) | 25 (80.65%) | 24 (77.42%) |

© 2022 Liu K et al. *JAMA Network Open.*

**eTable 10.** AUPRC of Models in 31 High Risk DRG Subgroups With At Least 20 AKI Patients

*, **, ***means models' performances are different to PMTL's in significance of P<0.1, P<0.01, P<0.001 respectively. WS, weighted sample; TL, transfer learning. PMTL, personalized model with transfer learning (proposed model). For PM-kNN, similar sample are matched based on k-Nearest Neighbor (similarity measure is based on features' coefficient in global logistic regression). The subgroups presented in Result section are those with at least 50 AKI patients. Full names of the APR-DRG for all subgroups are in eTable 8.

| Drg | PMTL | Global model | PM | PM-kNN & TL & WS | PM-kNN & WS | PM-kNN & TL | PM-kNN | Subgroup model & TL | Subgroup model |
|---|---|---|---|---|---|---|---|---|---|
| LIVER TRANSPLANT | 0.949 | 0.83*** | 0.926* | 0.933* | 0.884** | 0.918** | 0.874*** | 0.94 | 0.931 |
| BONE MARROW TRANSPLANT | 0.419 | 0.392 | 0.417 | 0.401 | 0.386 | 0.385* | 0.369 | 0.386 | 0.37 |
| TRACH W/DMV W EXTEN PROC | 0.926 | 0.836** | 0.865* | 0.925 | 0.883* | 0.921 | 0.881* | 0.919 | 0.933 |
| TRACH W/DMV W/O ENTEN PROC | 0.544 | 0.547 | 0.604 | 0.476 | 0.548 | 0.469 | 0.553 | 0.633 | 0.777* |
| RESPIRATORY SYSTEM DIAG | 0.594 | 0.544 | 0.578 | 0.516 | 0.506 | 0.51 | 0.517 | 0.623 | 0.652 |
| CYSTIC FIBROSIS | 0.358 | 0.312* | 0.346 | 0.374 | 0.371 | 0.368 | 0.345 | 0.329 | 0.277* |
| PULMONARY EDEMA & RESP FAIL | 0.387 | 0.358 | 0.401 | 0.365 | 0.345 | 0.354 | 0.306* | 0.281* | 0.296 |
| MAJOR RESPIRATORY INFECTIONS | 0.253 | 0.198* | 0.186* | 0.268 | 0.32 | 0.292 | 0.313 | 0.162* | 0.128** |
| CARDIAC VALVE PROC W/CATH | 0.866 | 0.611*** | 0.85 | 0.737*** | 0.802 | 0.735*** | 0.771* | 0.77** | 0.876 |
| CARDIAC VALVE PROC W/0 CATH | 0.661 | 0.493*** | 0.665 | 0.583*** | 0.595* | 0.571*** | 0.555*** | 0.572** | 0.629 |
| CORONARY BYPASS W/CARD CATH | 0.763 | 0.525*** | 0.728* | 0.72*** | 0.696** | 0.717*** | 0.667*** | 0.746 | 0.751 |
| COR BYPASS W/O CARD CATH | 0.716 | 0.506*** | 0.749 | 0.667*** | 0.662* | 0.65*** | 0.619*** | 0.688 | 0.745 |
| OTHER CARDIOTHORACIC PROC | 0.492 | 0.4 | 0.466 | 0.369* | 0.32* | 0.392 | 0.304* | 0.402 | 0.468 |
| MAJ THORACIC & ABDOMEN PROC | 0.460 | 0.513 | 0.541 | 0.43 | 0.511 | 0.454 | 0.527 | 0.512 | 0.532 |
| HEART FAILURE | 0.328 | 0.298 | 0.291 | 0.33 | 0.296 | 0.325 | 0.292 | 0.318 | 0.291 |
| OTH CIRCULATORY SYSTEM DIAG | 0.197 | 0.166 | 0.148* | 0.202 | 0.151 | 0.197 | 0.144* | 0.195 | 0.13* |
| MAJ STOM, ESOPH,DUOD PROC | 0.376 | 0.386 | 0.331 | 0.335* | 0.291* | 0.322* | 0.288* | 0.329 | 0.322 |
| MAJ SMALL & LARGE BOWEL PROC | 0.466 | 0.414* | 0.428 | 0.458 | 0.406* | 0.451 | 0.384* | 0.499 | 0.348*** |
| AMPUTATION OF LOWER LIMB | 0.360 | 0.373 | 0.377 | 0.402 | 0.344 | 0.402 | 0.35 | 0.281 | 0.264 |
| OTHER SKIN & SUBCUT PROC | 0.308 | 0.343 | 0.215 | 0.326 | 0.311 | 0.332 | 0.317 | 0.328 | 0.417 |
| MAJOR BLADDER PROC | 0.444 | 0.353* | 0.436 | 0.363* | 0.376 | 0.372* | 0.363 | 0.254*** | 0.297* |
| KIDNEY/URINARY TRACT MALIG | 0.341 | 0.335 | 0.311 | 0.317 | 0.302 | 0.317 | 0.289 | 0.333 | 0.312 |
| KIDNEY/URIN TRACT-NONMALIG | 0.583 | 0.275*** | 0.628 | 0.557 | 0.661 | 0.553 | 0.584 | 0.665* | 0.666 |
| ACUTE LEUKEMIA | 0.582 | 0.588 | 0.42** | 0.592 | 0.437** | 0.584 | 0.426** | 0.492* | 0.314*** |
| CHEMOTHERAPY | 0.664 | 0.566*** | 0.653 | 0.663 | 0.642 | 0.643* | 0.603** | 0.673 | 0.629 |

© 2022 Liu K et al. *JAMA Network Open.*

| Drg | PMTL | Global model | PM | PM-kNN & TL & WS | PM-kNN & WS | PM-kNN & TL | PM-kNN | Subgroup model & TL | Subgroup model |
|---|---|---|---|---|---|---|---|---|---|
| INFECT & PARASITIC DISEASE | 0.608 | 0.573 | 0.575 | 0.598 | 0.551* | 0.6 | 0.54* | 0.508*** | 0.514* |
| POST-OP/TRAUMA INFECT PROC | 0.350 | 0.321 | 0.317 | 0.391 | 0.332 | 0.377 | 0.314 | 0.324 | 0.256* |
| SEPTICEMIA & DISSEM INFECT | 0.364 | 0.331* | 0.299*** | 0.355 | 0.324* | 0.358 | 0.318* | 0.326** | 0.251*** |
| FULL THICK BURNS W GRAFT | 0.435 | 0.416 | 0.384 | 0.36* | 0.367 | 0.359* | 0.371 | 0.352 | 0.325 |
| HIV W MULTIPLE MAJOR HIV | 0.330 | 0.259 | 0.299 | 0.338 | 0.345 | 0.323 | 0.307 | 0.337 | 0.387 |
| EXTENSIVE PROC UNREL PDX | 0.447 | 0.396 | 0.39 | 0.411 | 0.37 | 0.424 | 0.352* | 0.364 | 0.313* |
| Times of PMTL outperforming | - | 25 (80.65%) | 24 (77.42%) | 22 (70.97%) | 24 (77.42%) | 24 (77.42%) | 26 (83.87%) | 23 (74.19%) | 22 (70.97%) |

© 2022 Liu K et al. *JAMA Network Open.*

**eTable 11.** Superiority of PMTL in Recalling AKI Patients in All Top 20 High-Risk Subgroups

There are total 2241 (19.8%) AKI patients in these subgroups. In this experiment, according to estimation of different model, we predict top-k patients with highest estimated risk as AKI patients (and the remaining were predicted as non-AKI), then calculated the recall with different model and different thresholds.

| **Thresholds** | **GM** | **SM** | **PMTL** | **PMTL/ GM** | **PMTL/ SM** |
|---|---|---|---|---|---|
| Top-500 from all subgroups | 15.98% | 18.03% | 19.32% | 120.95% | 107.18% |
| Top-1120 from all subgroups | 29.00% | 31.33% | 34.18% | 117.85% | 109.12% |
| Top-2241 from all subgroups | 46.59% | 48.33% | 52.16% | 111.97% | 107.94% |
| Top-10% from each subgroup | 26.24% | 26.55% | 30.12% | 114.80% | 113.45% |
| Top-20% from each subgroup | 42.57% | 43.60% | 47.93% | 112.58% | 109.93% |
| Top-50% AKI incidence rate from each subgroup | 27.35% | 28.96% | 32.17% | 117.62% | 111.09% |
| Top-AKI incidence rate from each subgroup | 46.36% | 48.15% | 52.34% | 112.90% | 108.71% |

© 2022 Liu K et al. *JAMA Network Open.*

**eTable 12.** Superiority of PMTL in Recalling AKI Patients in Each of Top 20 High-Risk Subgroups

In this experiment, according to estimation of different model, for each subgroups, we predict top-k (k is equal to the number of AKI patients in the subgroup) patients with highest estimated risk as AKI patients (and the remaining were predicted as non-AKI), then calculated the recall with different model and different thresholds.

| **Subgroups** | **GM** | **SM** | **PMTL** | **PMTL / GM** | **PMTL / SM** |
|---|---|---|---|---|---|
| Liver Transplant | 79.34% | 84.30% | 86.78% | 109.38% | 102.94% |
| Bone Marrow Transplant | 38.61% | 36.63% | 41.58% | 107.69% | 113.51% |
| Tracheostomy W Long Term Mechanical Ventilation W Extensive Procedure | 77.53% | 86.52% | 86.52% | 111.59% | 100.00% |
| Cystic Fibrosis - Pulmonary Disease | 31.45% | 31.45% | 36.29% | 115.38% | 115.38% |
| Pulmonary Edema & Respiratory Failure | 36.07% | 32.79% | 40.98% | 113.64% | 125.00% |
| Cardiac Valve Procedures w/ Cardiac Catheterization | 57.63% | 77.97% | 77.97% | 135.29% | 100.00% |
| Cardiac Valve Procedures w/o Cardiac Catheterization | 51.28% | 58.97% | 59.83% | 116.67% | 101.45% |
| Coronary Bypass w/ Cardiac Cath Or Percutaneous Cardiac Procedure | 51.47% | 66.91% | 65.44% | 127.14% | 97.80% |
| Coronary Bypass w/o Cardiac Cath Or Percutaneous Cardiac Procedure | 51.56% | 62.50% | 60.16% | 116.67% | 96.25% |
| Heart Failure | 26.89% | 31.09% | 35.29% | 131.25% | 113.51% |
| Major Stomach, Esophageal & Duodenal Procedures | 36.92% | 33.85% | 40.00% | 108.33% | 118.18% |
| Major Small & Large Bowel Procedures | 44.06% | 39.16% | 49.65% | 112.70% | 126.79% |
| Kidney & Urinary Tract Malignancy | 38.00% | 28.00% | 28.00% | 73.68% | 100.00% |
| Kidney & Urinary Tract Procedures for Nonmalignancy | 29.17% | 61.11% | 56.94% | 195.24% | 93.18% |
| Acute Leukemia | 54.90% | 37.25% | 52.94% | 96.43% | 142.11% |
| Chemotherapy | 55.92% | 62.09% | 58.77% | 105.08% | 94.66% |
| Infectious & Parasitic Diseases Including HIV W O.R. Procedure | 53.72% | 47.93% | 60.33% | 112.31% | 125.86% |
| Post-Op,Post-Trauma, Other Device Infections W O.R. Procedure | 32.43% | 27.03% | 32.43% | 100.00% | 120.00% |
| Septicemia & Disseminated Infections | 35.82% | 28.37% | 37.25% | 104.00% | 131.31% |
| Extensive Procedure Unrelated To Principal Diagnosis | 50.00% | 36.00% | 50.00% | 100.00% | 138.89% |

© 2022 Liu K et al. *JAMA Network Open.*

**eTable 13.** Details About 55 AKI Prediction Researches for Specific Subgroup Patients Can Be Identified by Our Data

To compare PMTL with models reported in existing literatures, we identified 136 AKI prediction studies published before 2021 in Web of Science and PubMed using keywords related to "AKI", "prediction" and "machine learning", 104/136 used all-stage AKI as the target. We could not confirm subgroups in 49/104 studies (because absence of corresponding features to identify these subgroups in our data). The remaining 55 papers are summarized in this Table. Research presented in Table 1 of the main text are highlighted in bold (at least one study per subgroup was selected, but studies with similar race distribution and AKI definition to ours, large sample size and independent validation are preferred). PCI, AMI, CABG, TKA, and GI stand for percutaneous coronary intervention, acute myocardial infarction, coronary artery bypass grafting, total knee arthroplasty, and gastrointestinal respectively. Bs, CV, DV, EV, EVR, IV stands for bootstrap validation, cross-validation, derivation validation, external validation, external validation research (verify performance of models presented in other researches), and internal validation respectively. For researches studies multiple modeling approaches, we just report AUROC of logistic regression and the best model. Default modeling method is Logistic Regression. LR, BN, GBM, RF stand for Logistic Regression, Bayesian networks, Gradient Boosting Machine, and Random Forest respectively.

| Subgroup | Cohorts | Region | Data sources | AKI definition | Sample size | AUROC | Ref. |
|---|---|---|---|---|---|---|---|
| Liver Transplant | Orthotopic LT without venovenous bypass | China | 1 center | SCr>0.5 mg/dL and ↑ ≥50% or RRT | 146 | IV: 0.908, 0.765 | 50 |
| | **Living-donor liver transplantation** | **Korea** | **1 center** | **AKIN** | **1211** | **IV: GBM: 0.90**<br>**IV: LR: 0.61** | 51 |
| | **Living donor liver transplantation** | **Korea** | **1 center** | **RIFLE** | **538** | **CV: 0.86-0.85** | 52 |
| PCI | **PCI** | **Canada** | **Provincial registry** | **KDIGO** | **7888** | **EVR: 0.65-0.76** | 53 |
| | PCI | China | 1 center | SCr↑ ≥25% or ≥0.5 mg/dL | 2500 | IV: 0.82 | 54 |
| | PCI | Japan | 16-center registry | AKIN | 5936 | IV: 0.799, 0.789 | 55 |
| | PCI | Japan | 16-center registry | AKIN | 11041 | EVR: 0.76 | 56 |
| | **The NCDR Cath-PCI registry** | **U.S.** | **National registry** | **AKIN** | **$9.5*10^5$** | **IV: 0.71** | 57 |
| | **PCI** | **U.S.** | **National registry** | **AKIN** | **$1.9*10^6$** | **IV: LR: 0.717-0.733**<br>**IV: GBM: 0.715-0.752**<br>**EV: 0.785** | 58 |
| | **PCI** | **U.S.** | **National registry** | **SCr increase > 0.3 mg/dL** | **$3*10^6$** | **IV: GBM: 0.777**<br>**EV: GBM: 0.794** | 59 |
| | PCI and eGFR < 60 mL/min/1.73 m2 | Vietnam | 1 center | AKIN | 135 | EVR: 0.64, 0.68 | 60 |
| PCI or Cardiac Catheterization | **Cardiac catheterization or PCI** | **U.S.** | **1 center** | **AKIN** | **1507** | **EVR: 0.73-0.74** | 61 |

© 2022 Liu K et al. *JAMA Network Open.*

| Subgroup | Cohorts | Region | Data sources | AKI definition | Sample size | AUROC | Ref. |
|---|---|---|---|---|---|---|---|
| PCI & AMI | Primary PCI for STEMI | Brazil | 1 center | AKIN | 347 | DV: 0.733<br>EVR: 0.649 | 62 |
| | STEMI undergoing primary PCI | China | 1 center | SCr↑ ≥50% or ≥0.3 mg/dL | 251 | EVR: 0.723, 0.688 | 63 |
| | PCI with drug-eluting stents | South Korea | 8-center registry | AKIN | 2189 | DV: 0.768 | 64 |
| | **Diagnostic angiography and PCI for acute STEMI.** | **U.S.** | **1 center** | **RIFLE** | **1144** | **IV: 0.76** | 65 |
| | **STEMI patients in the NCDR Cath-PCI registry** | **U.S.** | **National registry** | **AKIN** | **$1.5x10^5$** | **IV: 0.74** | 60 |
| PCI & non-AMI | **Non-STEMI/unstable angina patients in the NCDR Cath-PCI registry** | **U.S.** | **National registry** | **AKIN** | **$2.7x10^5$** | **IV:0.7** | 60 |
| AMI | **AMI** | **China** | **1 center** | **KDIGO** | **1495** | **IV: RF: 0.72-0.817<br>IV: GBM: 0.74<br>IV: LR: 0.681** | 66 |
| | **AMI** | **China** | **1 center** | **AKIN** | **6014** | **IV: 0.81-0.79<br>EVR: 0.73** | 67 |
| CABG | **LVEF< 50% and underwent isolated CABG** | **China** | **7 centers** | **KDIGO** | **1748** | **EV: 0.738** | 68 |
| | Isolated CABG were enrolled | Taiwan | 1 center | KDIGO | 353 | EVR: 0.78-0.697 | **69** |
| | **Coronary artery surgery** | **Turkey** | **1 center** | **RIFLE** | **193** | **EVR: 0.84, 0.5** | 70 |
| CABG or VS | **Elective CABG, valve surgery, or both** | **Brazil** | **1 center** | **Baseline Cr<1.5 mg/dL:Cr >2 mg/dL<br>Baseline 1.5≤Cr≤3 mg/dL: Cr↑ ≥50%** | **818** | **IV: 0.847** | 71 |
| | Valve or coronary artery bypass grafting | Canada | 1 center | KDIGO | 350 | DV: 0.78, 0.69 | 72 |
| | CABG alone, mitral or aortic valve surgery alone, or combination of CABG and aortic or mitral valve surgery with CPB | China | 1 center | KDIGO | 1587 | EVR: 0.61 | 73 |
| | **CABG, off-pump coronary artery bypass, valve surgery, or valve combined with CABG** | **China** | **1 center** | **KDIGO** | **8385** | **IV: 0.82-0.74** | 74 |
| | CABG or valve surgery | Singapore | 2 centers | AKIN | 2885 | DV: 0.70<br>IV: 0.75 | 75 |
| | CABG or valve replacement surgery | Taiwan | 1 center | KDIGO | 671 | IV: LR:0.806<br>IV: Ensemble: 0.843 | 76 |
| CABG or VS and Older | **Aged ≥ 60, underwent valve surgery or CABG with CPB** | **China** | **1 center** | **KDIGO** | **848** | **IV: 0.801<br>EVR: 0.627, 0.670** | 77 |

© 2022 Liu K et al. *JAMA Network Open.*

| Subgroup | Cohorts | Region | Data sources | AKI definition | Sample size | AUROC | Ref. |
|---|---|---|---|---|---|---|---|
| Cardiac Surgery | **Cardiac surgery** | **Australian and New Zealand** | **33 centers** | **KDIGO** | **22731** | **IV: 0.67-0.72** | 78 |
| | On-pump cardiac surgery | Belgium | 1 center | KDIGO | 283 | EVR: 0.80, 0.75 | 79 |
| | Elective cardiac surgery, exclusion planned off-pump procedure | Canada | 1 center | KDIGO | 289 | DV: 0.78, 0.74<br>EVR: 0.72-0.6 | 80 |
| | Cardiac surgery | Canada | 1 center | SCr↑ ≥50% | 2316 | EVR: 0.61 | 81 |
| | Cardiac surgery | China | 1 center | KDIGO | 1900 | IV: 0.789 | 82 |
| | Cardiac surgeries | China | 1 center | KDIGO | 5533 | CV: BN:0.728<br>IV: BN: 0.736 | 83 |
| | Cardiac surgery | Croatia | 1 center | KDIGO | 1056 | DV: 0.78<br>EVR: 0.73-0.634 | 84 |
| | Cardiac surgery with CPB, admitted to ICU | Holland | 1 center | RIFLE | 1388 | EVR: 0.75-0.65 | 85 |
| | Cardiac surgery with CPB | Italy | 1 center | KDIGO | 7675 | EVR: 0.59-0.54 | 86 |
| | Cardiac surgery with CPB | Norway | 1 center | AKIN | 1015 | Bs: 0.81, 0.83 | 87 |
| | **Open heart surgery** | **Norway** | **1 center** | **AKIN** | **5029** | **Bs: 0.819** | 88 |
| | Cardiac surgery with CPB | Spain | 2 centers | RIFLE | 1542 | IV: 0.81 | 89 |
| | **Cardiac surgery, including thoracic aorta** | **U.K.** | **3 centers** | **KDIGO** | **30854** | **EV: 0.74<br>EVR: 0.74-0.68** | 90 |
| TKA | **Unilateral or bilateral TKA** | **Korea** | **3 centers** | **KDIGO** | **5757** | **CV: GBM: 0.78<br>EV: GBM: 0.89** | 91 |
| Orthopaedic surgery | **Replacement or Fusion of joint, reduction of fracture** | **U.K.** | **3 centers** | **KDIGO** | **10615** | **Bs: 0.73<br>EV: 0.70** | 92 |
| GI Surgery | **Elective or emergency GI resection, liver resection or stoma reversal** | **UK and Ireland** | **173 centers** | **KDIGO** | **4544** | **Bs: 0.65** | 93 |
| GI Cancers | **GI cancers** | **China** | **1 center** | **KDIGO** | **6495** | **IV: BN: 0.79<br>IV: LR: 0.7** | 94 |
| Hematologic Malignancies | **Lymphoma, leukemia or multiple myeloma** | **China** | **1 center** | **KDIGO** | **2395** | **CV: BN: 0.812<br>DV: LR: 0.763** | 95 |
| Cisplatin | **Received cisplatin** | **U.S.** | **2 registries** | **AKIN** | **4481** | **EV: 0.70** | 96 |
| Vancomycin | **Received vancomycin ≥48h** | **China** | **1 center** | **KDIGO** | **524** | **EV: 0.788** | 97 |
| Vancomycin & Older | **aged ≥ 60, received vancomycin≥48h** | **China** | **1 center** | **KDIGO** | **255** | **IV: 0.736** | 98 |

© 2022 Liu K et al. *JAMA Network Open.*

| Subgroup | Cohorts | Region | Data sources | AKI definition | Sample size | AUROC | Ref. |
|---|---|---|---|---|---|---|---|
| Sepsis | Sepsis in ICU | China | 1 center | KDIGO | 410 | Bs: 0.969<br>EV: 0.957 | 99 |
| | Sepsis in ICU | China | 1 center | AKIN | 2331 | IV: 0.857 | 100 |
| | **Sepsis in ICU** | **U.S.** | **MCMC III** | **KDIGO** | **2917** | **IV:0.79** | 101 |
| | **Sepsis in ICU** | **U.S.** | **MCMC III** | **KDIGO** | **15726** | **IV: 0.712** | 102 |
| Older | **Age > 60** | **U.S.** | **15 centers** | **AKIN** | **25521** | **CV: Ensemble: 0.664**<br>**CV: LR: 0.660** | 103 |
| | **Age > 60** | **U.S.** | **15 centers** | **AKIN** | **44691** | **CV: 0.57-0.724** | 104 |

© 2022 Liu K et al. *JAMA Network Open.*

**eTable 14.** AUPRC Comparison of Models in 20 Well-Studied Subgroups

PCI, percutaneous coronary intervention; AMI, acute myocardial infarction; CABG, coronary artery bypass grafting; VS, valve surgery; TKA, total knee arthroplasty; Orthop., orthopedic; GI, gastrointestinal. For studies that used multiple modeling approaches, we only report the AUROC of the logistic regression model and the best model. *P<0.05 compared with PMTL. GM = Global Model; SM = Subgroup Model; SMTL = Subgroup Model with Transfer Learning; PMTL = Personalized Model with Transfer Learning.

| **Subgroups in literature** | **PMTL** | **GM** | **SM** | **SMTL** |
|---|---|---|---|---|
| Liver Transplant | 0.949 | 0.83* | 0.931 | 0.94 |
| PCI | 0.235 | 0.22 | 0.236 | 0.278 |
| PCI or Cardiac Catheterization | 0.233 | 0.222 | 0.256 | 0.301* |
| PCI & AMI | 0.263 | 0.203* | 0.307 | 0.232 |
| PCI & non-AMI | 0.232 | 0.26 | 0.189 | 0.24 |
| AMI | 0.263 | 0.199* | 0.23 | 0.244 |
| CABG | 0.738 | 0.513* | 0.748 | 0.742 |
| CABG or VS | 0.738 | 0.52* | 0.774* | 0.738 |
| CABG or VS and age:55-65 | 0.741 | 0.577* | 0.706 | 0.641* |
| CABG or VS and age>65 | 0.737 | 0.515* | 0.777 | 0.708 |
| Cardiac Surgery | 0.725 | 0.511* | 0.726 | 0.729 |
| TKA | 0.096 | 0.09 | 0.08 | 0.085 |
| Orthop. surgery | 0.194 | 0.181 | 0.176 | 0.206 |
| GI Surgery | 0.397 | 0.352* | 0.299* | 0.381 |
| GI Cancers | 0.338 | 0.252 | 0.15 | 0.21 |
| Hematologic Malignancies | 0.385 | 0.357 | 0.302* | 0.37 |
| Cisplatin | 0.189 | 0.177 | 0.149 | 0.208 |
| Vancomycin | 0.380 | 0.326* | 0.308* | 0.346* |
| Vancomycin & age:55-65 | 0.384 | 0.336* | 0.254* | 0.325* |
| Vancomycin & age>65 | 0.389 | 0.321* | 0.265* | 0.347* |
| Sepsis | 0.364 | 0.331* | 0.251* | 0.326* |
| Age:55-65 | 0.395 | 0.347* | 0.298* | 0.337* |
| Age>65 | 0.371 | 0.305* | 0.284* | 0.323* |
| Times of PMTL outperforming | - | 22 (95.65%) | 16 (69.57%) | 15 (65.22%) |
| Times of PMTL significantly better vs worse | - | 15 : 0 | 8 : 1 | 7 : 1 |

© 2022 Liu K et al. *JAMA Network Open.*

**eTable 15.** List of APR-DRG Presented in Fig 4 of Main Text

| Abbreviations | APR-DRG |
|---|---|
| **Cardiac surgery** | |
| CARDIAC VALVE PROC W/CATH | Cardiac Valve Procedures w/ Cardiac Catheterization |
| COR BYPASS W/O CARD CATH | Coronary Bypass w/o Cardiac Cath Or Percutaneous Cardiac Procedure |
| CORONARY BYPASS W/CARD CATH | Coronary Bypass w/ Cardiac Cath Or Percutaneous Cardiac Procedure |
| CARDIAC VALVE PROC W/O CATH | Cardiac Valve Procedures w/o Cardiac Catheterization |
| OTHER CARDIOTHORACIC PROC | Other Cardiothoracic Procedures |
| **PCI & Cardiac catheter** | |
| PERCUT CARDIOVASC PROC W/AMI | Percutaneous Cardiovascular Procedures w/ AMI |
| PERCUT CARDIOVASC W/O AMI | Percutaneous Cardiovascular Procedures w/o AMI |
| CARD CATHET EXC ISCHEMIA | Cardiac Catheterization w/ Circ Disord Exc Ischemic Heart Disease |
| CARD CATHET W/ISCHEMIA | Cardiac Catheterization for Ischemic Heart Disease |
| **Other cardiac disease** | |
| OTH CIRCULATORY SYSTEM DIAG | Other Circulatory System Diagnoses |
| HYPERTENSION | Hypertension |
| PERIPHERAL & OTH VASC DIS | Peripheral & Other Vascular Disorders |
| ANGINA PECT & CORONARY ATH | Angina Pectoris & Coronary Atherosclerosis |
| CARD ARRHYTHMIA & CONDUCTION | Cardiac Arrhythmia & Conduction Disorders |
| **Pulmonary vascular** | |
| PULMONARY EMBOLISM | Pulmonary Embolism |
| MAJOR CHEST & RESP TRAUMA | Major Chest & Respiratory Trauma |
| **Respiratory failure** | |
| TRACH W/DMV W EXTEN PROC | Tracheostomy W Long Term Mechanical Ventilation W Extensive Procedure |
| TRACH W/DMV W/O ENTEN PROC | Tracheostomy W Long Term Mechanical Ventilation W/O Extensive Procedure |
| PULMONARY EDEMA & RESP FAIL | Pulmonary Edema & Respiratory Failure |
| **Respiratory infection** | |
| CYSTIC FIBROSIS | Cystic Fibrosis - Pulmonary Disease |
| MAJOR RESPIRATORY INFECTIONS | Major Respiratory Infections & Inflammations |
| OTHER PNEUMONIA | Other Pneumonia |
| CHRONIC OBSTRUCTIVE PULM DIS | Chronic Obstructive Pulmonary Disease |
| **Systemic infection** | |
| INFECT & PARASITIC DISEASE | Infectious & Parasitic Diseases Including HIV W O.R. Procedure |
| POST-OP/TRAUMA INFECT PROC | Post-Op,Post-Trauma, Other Device Infections W O.R. Procedure |
| SEPTICEMIA & DISSEM INFECT | Septicemia & Disseminated Infections |
| OTH INFECT & PARASITIC DIS | Other Infectious & Parasitic Diseases |
| POST-OP/POST-TRAUM INFEC | Post-Operative, Post-Traumatic, Other Device Infections |
| | |
| CELLULITIS & OTHER BACT INF | Cellulitis & Other Bacterial Skin Infections |
| | |
| KIDNEY/URIN TRACT INFECT | Kidney & Urinary Tract Infections |
| **Cerebrovascular** | |
| EXTRACRANIAL VASCULAR PROC | Extracranial Vascular Procedures |
| INTRACRANIAL HEMORRHAGE | Intracranial Hemorrhage |

© 2022 Liu K et al. *JAMA Network Open.*

| Abbreviations | APR-DRG |
|---|---|
| HEAD TRAUMA W COMA >1 HR | Head Trauma w/ Coma > 1 Hr or Hemorrhage |
| CVA W INFARCT | CVA & Precerebral Occulusion w/ Infarct |
| CRANIOTOMY EXC FOR TRAUMA | Craniotomy Except for Trauma |
| **Cranial nerve** | |
| PERIPHERAL & CRANIAL NERV | Peripheral, Cranial & Autonomic Nerve Disorders |
| DEGENERATIVE NERV SYST DIS | Degenerative Nervous System Disorders Exc Mult Sclerosis |
| SEIZURE | Seizure |
| OTH DISORDERS NERVOUS SYS | Other Disorders of Nervous System |
| MIGRAINE & OTHER HEADACHE | Migraine & Other Headaches |
| **Hepatobiliary surgery** | |
| LIVER TRANSPLANT | Liver Transplant |
| OTH HEPATOBILI/PANCREAS PROC | Other Hepatobiliary, Pancreas & Abdominal Procedures |
| MAJ PANCREAS/LIVER/SHUNT PROC | Major Pancreas, Liver & Shunt Procedures |
| MAJOR BILIARY TRACT PROC | Major Biliary Tract Procedures |
| **Hepatobiliary disease** | |
| MALIGNANCY-HEPATOBILIA SYS | Malignancy of Hepatobiliary System & Pancreas |
| HEPATIC COMA/OTH MAJ LIV DIS | Hepatic Coma & Other Major Acute Liver Disorders |
| OTHER DISORDERS OF LIVER | Other Disorders Of The Liver |
| ALCOHOLIC LIVER DISEASE | Alcoholic Liver Disease |
| **Gastrointestinal surgery** | |
| MAJ SMALL & LARGE BOWEL PROC | Major Small & Large Bowel Procedures |
| MAJ STOM, ESOPH,DUOD PROC | Major Stomach, Esophageal & Duodenal Procedures |
| OTH SMALL & LARGE BOWEL PROC | Minor Small & Large Bowel Procedures |
| **Joint replacement** | |
| HIP JOINT REPLACEMENT | Hip Joint Replacement |
| KNEE JOINT REPLACEMENT | Knee Joint Replacement |
| **Other orthopeadic Surgery** | |
| AMPUTATION OF LOWER LIMB | Amputation of Lower Limb Except Toes |
| HIP & FEMUR PROC NONTRAUMA | Hip & Femur Procedures for Non-Trauma Except Joint Replacement |
| HIP & FEMUR PROC TRAUM | Hip & Femur Procedures for Trauma Except Joint Replacement |
| SHOULDER,UP ARM,FOREARM PROC | Shoulder, Upper Arm & Forearm Procedures |
| KNEE & LOWER LEG PROC | Knee & Lower Leg Procedures Except Foot |
| **Hematology and immunology** | |
| BONE MARROW TRANSPLANT | Bone Marrow Transplant |
| ACUTE LEUKEMIA | Acute Leukemia |
| LYMPH/MYELO/NON-AC LEUK | Lymphoma & Non-acute Leukemia |
| MAJ HEMATOLOGIC/IMMUNOL DX | Major Hematologic/Immunologic Diag Exc Sickle Cell Crisis & Coagul |
| OTHER ANEMIA/BLOOD DISORD | Other Anemia & Disorders Of Blood & Blood Forming Organs |

© 2022 Liu K et al. *JAMA Network Open.*

**eTable 16.** Significant Interactions Between 6 Important Predictors and Disease in Meta-Regression and Their Verification

OR change is calculated by the OR of target predictor when moderators is happened dividing by the OR of target predictor in remaining patients and subtract 100%. In subgroup analysis, when target factor is age, exposed group contains patients with age>65, unexposed group contains patients with age<45; when target factor is BMI or pulse, unexposed group contains patients with BMI:18.5-25 and pulse: 50-80 respectively; when target factor is lab test, exposed group contains patients with abnormal result, unexposed group contains patients with normal result. Moderators significantly (p⩽0.05) verified by subgroup model, subgroup analysis and both are marked with #, †, and * respectively. Significant interactions or interactions we suggest need to be concerned are shown in bold.

| Target factor | Effect change in meta | Class of moderator | Detail of moderator | Subgroup model | | Subgroup analysis | | | |
|---|---|---|---|---|---|---|---|---|---|
| | | | | OR (se) | OR change (p-value) | Risk in exposed group | Risk in unexposed group | OR (se) | OR change (p-value) |
| **Age** | ↑ | ***Cardiac surgery** | **Cardiac Valve, Coronary Bypass, Cardiothoracic Procedures** | **1.430 (0.140)** | **31.6% (0.0052)** | **31.0% (298/960)** | **13.5% (33/244)** | **2.878 (0.575)** | **130.1% (0.0000)** |
| Age | ↑ | Other cardiovascular | Hypertension, Chest Pain, Angina Pectoris & Coronary Atherosclerosis, Cardiac Arrhythmia & Conduction Disorders, PCI w/o AMI, | 1.093 (0.061) | -0.7% (0.8860) | 10.7% (350/3273) | 8.8% (63/717) | 1.243 (0.178) | -10.3% (0.4618) |
| Age | ↑ | Pregnancy | Cesarean and Vaginal Delivery, Other Antepartum Diagnoses | 0.899 (0.216) | -20.8% (0.2925) | 4.6% (4/87) | 5.8% (35/599) | 0.777 (0.420) | -35.0% (0.4279) |
| Age | ↑ | Delivery | Cesarean and Vaginal Delivery | 0.968 (0.269) | -15.3% (0.4722) | 5.9% (4/68) | 7.2% (32/443) | 0.803 (0.439) | -33.3% (0.4607) |
| Age | ↑ | Cerebrovascular | Head Trauma with/ Commaor Hemorrhage; Intracranial Hemorrhage; Concussion, Closed Skull FX Nos; CVA & Precerebral Occulusion w/ Infarct | 1.125 (0.105) | 2.2% (0.8523) | 7.5% (49/652) | 4.0% (15/379) | 1.972 (0.596) | 42.3% (0.2461) |
| Age | ↑ | Intracranial Hemorrhage | All Intracranial Hemorrhage admission (contain insignificant admission) | 1.128 (0.122) | 2.1% (0.8893) | 8.9% (61/686) | 4.5% (15/337) | 2.095 (0.621) | 51.6% (0.1630) |
| **Age** | ↓ | ***Infection** | **Cellulitis & Other Bacterial Skin Infections, Cystic Fibrosis, Infectious & Parasitic Diseases, HIV, Respiratory Infections or Pneumonia, Post-Operative, Post-Traumatic, Other Device Infections** | **0.978 (0.038)** | **-13.3% (0.0005)** | **12.5% (227/1823)** | **15.7% (475/3026)** | **0.764 (0.066)** | **-53.2% (0.0000)** |
| **Age** | ↓ | ***Infection (Exc HIV and Cystic Fibrosis)** | **Cellulitis & Other Bacterial Skin Infections, Infectious & Parasitic Diseases, Respiratory Infections or Pneumonia, Post-Operative, Post-Traumatic, Other Device Infections** | **0.985 (0.038)** | **-12.1% (0.0021)** | **12.5% (227/1818)** | **14.6% (314/2148)** | **0.833 (0.078)** | **-44.7% (0.0000)** |
| **Age** | ↓ | ***Septicemia & Disseminated Infections** | **Septicemia & Disseminated Infections** | **0.905 (0.065)** | **-18.6% (0.0044)** | **13.4% (103/771)** | **16.6% (150/906)** | **0.777 (0.108)** | **-46.1% (0.0000)** |

© 2022 Liu K et al. *JAMA Network Open.*

| Target factor | Effect change in meta | Class of moderator | Detail of moderator | Subgroup model | | Subgroup analysis | | | |
|---|---|---|---|---|---|---|---|---|---|
| | | | | OR (se) | OR change (p-value) | Risk in exposed group | Risk in unexposed group | OR (se) | OR change (p-value) |
| **Age** | ↓ | ***Major hematological disease** | **Bone Marrow Transplant, Acute Leukemia, Major Hematologic/Immunologic Diag Exc Sickle Cell Crisis & Coagul** | **0.833 (0.076)** | **-25.2% (0.0013)** | **12.3% (42/341)** | **17.0% (72/424)** | **0.687 (0.144)** | **-51.4% (0.0007)** |
| **Age** | ↓ | ***Bone Marrow Transplant** | **Bone Marrow Transplant** | **0.866 (0.110)** | **-21.9% (0.0467)** | **12.2% (12/98)** | **29.8% (42/141)** | **0.329 (0.118)** | **-76.7% (0.0001)** |
| Age | ↓ | Orthopedic surgery | Foot & Toe Procedures; Knee & Lower Leg Procedures; Amputation of Lower Limb; Tendon, Muscle & Other Soft Tissue Procedures; Other Musculoskeletel System & Connective Tissue Procedures | 1.037 (0.125) | -6.1% (0.5623) | 10.3% (43/417) | 5.6% (39/696) | 1.937 (0.446) | 41.3% (0.1378) |
| Age | ↓ | Orthopedic surgery | Foot & Toe Procedures; Tendon, Muscle & Other Soft Tissue Procedures; Amputation of Lower Limb | 1.016 (0.192) | -8.0% (0.5846) | 11.8% (16/136) | 12.5% (26/208) | 0.933 (0.316) | -32.9% (0.2411) |
| Age | ↓ | Gastrointestinal surgery | Small & Large Bowel Procedures, Major Biliary Tract Procedures | 0.989 (0.112) | -10.5% (0.3006) | 17.9% (79/442) | 10.7% (36/338) | 1.826 (0.394) | 33.4% (0.1870) |
| Age | ↓ | Gastrointestinal surgery | Small & Large Bowel Procedures, Major Biliary Tract Procedures; Stomach, Esophageal & Duodenal Procedures (contain insignificant admission) | 0.985 (0.099) | -11.3% (0.2202) | 16.6% (102/616) | 11.4% (50/438) | 1.540 (0.285) | 12.4% (0.5363) |
| **Serum calcium** | ↑ | ***Cardiac surgery** | **Cardiac Valve, Coronary Bypass, Cardiothoracic Procedures** | **4.404 (0.761)** | **290.1% (0.0000)** | **36.1% (365/1010)** | **16.7% (234/1401)** | **2.822 (0.274)** | **105.9% (0.0000)** |
| **Serum calcium** | ↑ | ***Mechanical Ventilation** | **Mechanical Ventilation** | **3.163 (0.679)** | **160.7% (0.0000)** | **69.9% (86/123)** | **37.6% (56/149)** | **3.860 (1.001)** | **167.5% (0.0002)** |
| **Serum calcium** | ↑ | ***Burn** | **Extensive 3rd Degree Burns, Full Thickness Burns** | **2.976 (0.969)** | **145.8% (0.0089)** | **25.0% (41/164)** | **9.5% (23/241)** | **3.159 (0.897)** | **117.2% (0.0065)** |
| **Serum calcium** | ↑ | **†Major surgery** | **Major Cranial/Facial Bone Procedures; Major Stomach, Esophageal & Duodenal Procedures; Major Respiratory & Chest Procedures; Major Small & Large Bowel Procedures** | **1.676 (0.353)** | **38.1% (0.1517)** | **18.2% (169/928)** | **9.7% (122/1262)** | **2.081 (0.266)** | **44.7% (0.0046)** |
| Serum calcium | ↑ | Percutaneous Cardiovascular Procedures w/o AMI | Percutaneous Cardiovascular Procedures w/o AMI | 1.329 (0.413) | 9.0% (0.8997) | 8.8% (24/272) | 7.4% (70/946) | 1.211 (0.299) | -17.4% (0.4413) |
| **Serum calcium** | ↓ | ***Cardiac Catheterization for Ischemic Heart Disease** | **Percutaneous Cardiovascular Procedures w/ AMI, Cardiac Catheterization for Ischemic Heart Disease** | **0.414 (0.115)** | **-66.4% (0.0001)** | **4.3% (9/210)** | **5.9% (68/1144)** | **0.709 (0.257)** | **-51.6% (0.0462)** |
| Serum calcium | ↓ | Other cardiovascular admission | Cardiac Congenital & Valvular Disorders; Chest Pain; Hypertension; Acute Myocardial Infarction; Cardiac Catheterization w/ Circ Disord Exc Ischemic Heart Disease; Angina Pectoris & Coronary Atherosclerosis; Heart Failure; Perm | 1.285 (0.303) | 5.3% (0.9105) | 12.2% (74/606) | 9.3% (331/3569) | 1.361 (0.186) | -8.0% (0.5516) |

© 2022 Liu K et al. *JAMA Network Open.*

| Target factor | Effect change in meta | Class of moderator | Detail of moderator | Subgroup model | | Subgroup analysis | | | |
|---|---|---|---|---|---|---|---|---|---|
| | | | | OR (se) | OR change (p-value) | Risk in exposed group | Risk in unexposed group | OR (se) | OR change (p-value) |
| | | | Cardiac Pacemaker Implant w/o AMI, Heart Failure or Shock; Other Circulatory System Diagnoses; Cardiac Arrhythmia & Conduction Disorders | | | | | | |
| Serum calcium | ↓ | Other cardiovascular (EXC Cardiac Arrhythmia & Conduction Disorders) | Cardiac Congenital & Valvular Disorders; Chest Pain; Hypertension; Acute Myocardial Infarction; Cardiac Catheterization w/ Circ Disord Exc Ischemic Heart Disease; Angina Pectoris & Coronary Atherosclerosis; Heart Failure; Perm Cardiac Pacemaker Implant w/o AMI, Heart Failure or Shock; Other Circulatory System Diagnoses | 1.031 (0.245) | -15.5% (0.4055) | 14.2% (63/443) | 12.7% (295/2328) | 1.143 (0.171) | -23.9% (0.0723) |
| **Serum calcium** | ↓ | ***Liver disease** | **Alcoholic Liver Disease; Hepatic Coma & Other Major Acute Liver Disorders; Other Disorders Of The Liver; Malignancy of Hepatobiliary System & Pancreas; Other Hepatobiliary, Pancreas & Abdominal Procedures** | **0.545 (0.136)** | **-55.9% (0.0006)** | **9.2% (91/986)** | **11.3% (93/820)** | **0.795 (0.124)** | **-46.6% (0.0001)** |
| **Serum calcium** | ↓ | ***Orthopedic** | **Fracture of Pelvis or Dislocation of Hip; Other Musculoskeletal System & Connective Tissue Diagnoses; Hip Joint Replacement; Fracture or Dislocation; Knee & Lower Leg Procedures Except Foot; Hip & Femur Procedures; Shoulder, Upper Arm & Forearm Procedures; Intervertebral Disc Excision & Decompression; Other Musculoskeletel System & Connective Tissue Procedures; Dorsal & Lumbar Fusion Proc Except for Curvature of Back** | **0.846 (0.126)** | **-33.1% (0.0062)** | **5.7% (211/3715)** | **5.7% (242/4233)** | **0.993 (0.096)** | **-36.3% (0.0000)** |
| **Serum calcium** | ↓ | ***Orthopedic (EXC lumbar & back)** | **Fracture of Pelvis or Dislocation of Hip; Other Musculoskeletal System & Connective Tissue Diagnoses; Hip Joint Replacement; Fracture or Dislocation Except Back; Knee Joint Replacement; Knee & Lower Leg Procedures Except Foot; Hip & Femur Procedures; Shoulder, Upper Arm & Forearm Procedures; Other Musculoskeletel System & Connective Tissue Procedures** | **0.720 (0.119)** | **-43.2% (0.0006)** | **5.3% (161/3025)** | **5.8% (202/3512)** | **0.921 (0.100)** | **-40.4% (0.0000)** |
| **Serum calcium** | ↓ | ***Respiratory diagnoses** | **COPD; Asthma; Pneumonia; Embolism; Edema & Respiratory Failure; Trauma; Cystic Fibrosis** | **0.850 (0.138)** | **-31.9% (0.0196)** | **10.7% (111/1039)** | **10.0% (338/3376)** | **1.075 (0.124)** | **-28.1% (0.0053)** |

© 2022 Liu K et al. *JAMA Network Open.*

| Target factor | Effect change in meta | Class of moderator | Detail of moderator | Subgroup model | | Subgroup analysis | | | |
|---|---|---|---|---|---|---|---|---|---|
| | | | | OR (se) | OR change (p-value) | Risk in exposed group | Risk in unexposed group | OR (se) | OR change (p-value) |
| **Serum calcium** | ↓ | ***Alimentary tract diseases** | **Gastrointestinal Vascular Insufficiency; Diverticulitis & Diverticulosis; Other & Unspecified Gastrointestinal Hemorrhage; Abdominal Pain; Peptic Ulcer & Gastritis; Inflammatory Bowel Disease; Major Esophageal Disorders; Other Digestive System Diagnoses; Major Gastrointestinal & Peritoneal Infections; Other Esophageal Disorders; Intestinal Obstruction; Digestive Malignancy; Non-Bacterial Gastroenteritis, Nausea & Vomiting** | **0.585 (0.121)** | **-53.5% (0.0001)** | **4.2% (93/2198)** | **4.8% (149/3127)** | **0.883 (0.119)** | **-41.8% (0.0001)** |
| Serum calcium | ↓ | Abuse | Cocaine, Opioid, Drug & Alcohol | 0.870 (0.238) | -28.8% (0.1834) | 2.1% (3/141) | 2.7% (16/591) | 0.781 (0.497) | -46.5% (0.3260) |
| Serum calcium | ↓ | Cranial nerve | Migraine & Other Headaches; Viral Meningitis; Peripheral, Cranial & Autonomic Nerve Disorders; Multiple Sclerosis & Other Demyelinating Diseases; Seizure | 1.181 (0.372) | -3.3% (0.7967) | 6.6% (24/362) | 4.8% (71/1493) | 1.422 (0.347) | -2.3% (0.9257) |
| Serum calcium | ↓ | Mental disease | Acute Anxiety & Delirium States; Depression; Schizophrenia; Bipolar Disorders; Organic Mental Health Disturbances | 0.947 (0.243) | -22.4% (0.2789) | 4.8% (3/63) | 2.8% (18/651) | 1.758 (1.122) | 20.9% (0.7660) |
| Serum calcium | ↓ | Mental disease | All Mental disease admission (contain insignificant admission) | 0.963 (0.271) | -21.2% (0.3351) | 4.2% (3/71) | 3.0% (21/709) | 1.445 (0.911) | -0.6% (0.9926) |
| **Serum calcium** | ↓ | ***Infection** | **Viral Meningitis; Kidney & Urinary Tract Infections; Respiratory Infections; Cystic Fibrosis; COPD; Major Gastrointestinal & Peritoneal Infections; Non-bacterial Infections of Nervous System; Osteomyelitis, Septic Arthritis & Other Musculoskeletal Infections; Cellulitis & Other Bacterial Skin Infections; Other Infectious & Parasitic Diseases** | **0.640 (0.103)** | **-49.9% (0.0000)** | **8.4% (117/1385)** | **10.1% (405/4028)** | **0.825 (0.091)** | **-45.8% (0.0000)** |
| **Blood glucose** | ↑ | **Major Pancreas, Liver & Shunt Procedures** | **Liver Transplant; Major Pancreas, Liver & Shunt Procedures** | **2.357 (0.641)** | **68.0% (0.0774)** | **40.1% (61/152)** | **23.0% (158/686)** | **2.240 (0.423)** | **42.4% (0.0646)** |
| **Blood glucose** | ↑ | ***Gastrointestinal surgery** | **Small & Large Bowel Procedures; Hernia Procedures; Major Stomach, Esophageal & Duodenal Procedures; Procedures for Obesity; Peritoneal Adhesiolysis; Cholecystectomy Except Laparoscopic; Major Biliary Tract Procedures; Other Digestive System & Abdominal Procedures** | **2.416 (0.609)** | **73.5% (0.0392)** | **24.3% (90/371)** | **10.7% (279/2608)** | **2.674 (0.365)** | **71.6% (0.0001)** |

© 2022 Liu K et al. *JAMA Network Open.*

| Target factor | Effect change in meta | Class of moderator | Detail of moderator | Subgroup model | | Subgroup analysis | | | |
|---|---|---|---|---|---|---|---|---|---|
| | | | | OR (se) | OR change (p-value) | Risk in exposed group | Risk in unexposed group | OR (se) | OR change (p-value) |
| Blood glucose | ↑ | Uterine & Adnexa Procedures | Uterine & Adnexa Procedures; Pelvic Evisceration, Radical Hysterectectomy & Radical GYN Procs | 1.710 (0.395) | 19.0% (0.5301) | 17.7% (14/79) | 7.9% (42/529) | 2.497 (0.838) | 57.5% (0.1775) |
| Blood glucose | ↑ | Uterine & Adnexa Procedures | All Uterine & Adnexa Procedures (contain insignificant admission) | 2.027 (0.658) | 42.2% (0.3515) | 12.3% (20/162) | 5.4% (69/1279) | 2.470 (0.664) | 56.4% (0.0984) |
| Blood glucose | ↑ | Urological surgery | Major Bladder Procedures; Kidney & Urinary Tract Procedures | 1.680 (0.484) | 17.8% (0.6717) | 25.9% (49/189) | 16.1% (150/932) | 1.825 (0.344) | 15.2% (0.4586) |
| **Blood glucose** | ↑ | ***Joint Replacement** | **Hip Joint Replacement; Knee Joint Replacement** | **2.876 (0.864)** | **102.4% (0.0294)** | **11.5% (30/261)** | **5.0% (157/3167)** | **2.490 (0.524)** | **59.8% (0.0276)** |
| Blood glucose | ↓ | Nervous disorder (non-surgery) | Vertigo & Other Labyrinth Disorders; Syncope & Collapse; Transient Ischemia; Multiple Sclerosis & Other Demyelinating Diseases; Degenerative Nervous System Disorders; Migraine & Other Headaches; Seizure; Other Disorders of Nervous System | 0.937 (0.261) | -34.6% (0.0908) | 6.0% (28/469) | 3.8% (94/2451) | 1.592 (0.353) | -0.1% (0.9981) |
| **Blood glucose** | ↓ | **#Cardiovascular condition (non-surgery)** | **Hypertension; Cardiac Arrhythmia & Conduction Disorders; Chest Pain; Other Circulatory System Diagnoses; Angina Pectoris & Coronary Atherosclerosis; Cardiac Catheterization for Ischemic Heart Disease** | **0.751 (0.191)** | **-47.5% (0.0090)** | **7.4% (31/421)** | **5.2% (141/2693)** | **1.439 (0.296)** | **-9.5% (0.6310)** |
| BMI>30 | ↑ | Respiratory System | Mechanical Ventilation; Pulmonary Edema & Respiratory Failure; Respiratory Infections or Pneumonia; Cystic Fibrosis; Major Chest & Respiratory Trauma; Respiratory Malignancy; COPD | 0.978 (0.221) | -29.5% (0.1018) | 16.1% (194/1208) | 14.0% (222/1589) | 1.178 (0.126) | -14.2% (0.1702) |
| BMI 25-30 | ↑ | Respiratory System | Mechanical Ventilation; Pulmonary Edema & Respiratory Failure; Respiratory Infections or Pneumonia; Cystic Fibrosis; Major Chest & Respiratory Trauma; Respiratory Malignancy; COPD | 1.276 (0.270) | 8.3% (0.7817) | 15.1% (128/850) | 14.0% (222/1589) | 1.092 (0.131) | -6.2% (0.6110) |
| BMI>25 | ↑ | Respiratory System | Mechanical Ventilation; Pulmonary Edema & Respiratory Failure; Respiratory Infections or Pneumonia; Cystic Fibrosis; Major Chest & Respiratory Trauma; Respiratory Malignancy; COPD | 1.131 (0.203) | -10.2% (0.5003) | 15.6% (322/2058) | 14.0% (222/1589) | 1.142 (0.108) | -11.0% (0.2399) |
| BMI>30 | ↑ | Infection | Septicemia & Disseminated Infections; Infectious & Parasitic Diseases Including HIV W O.R. Procedure; Kidney & Urinary Tract Infections; Cystic Fibrosis; COPD; Respiratory Infections or Pneumonia | 1.685 (0.264) | 25.7% (0.1868) | 15.6% (320/2048) | 13.5% (318/2355) | 1.186 (0.102) | -14.7% (0.0854) |

© 2022 Liu K et al. *JAMA Network Open.*

| Target factor | Effect change in meta | Class of moderator | Detail of moderator | Subgroup model | | Subgroup analysis | | | |
|---|---|---|---|---|---|---|---|---|---|
| | | | | OR (se) | OR change (p-value) | Risk in exposed group | Risk in unexposed group | OR (se) | OR change (p-value) |
| BMI 25-30 | ↑ | Infection | Septicemia & Disseminated Infections; Infectious & Parasitic Diseases Including HIV W O.R. Procedure; Kidney & Urinary Tract Infections; Cystic Fibrosis; COPD; Respiratory Infections or Pneumonia | 1.444 (0.231) | 22.8% (0.2263) | 12.9% (201/1561) | 13.5% (318/2355) | 0.947 (0.092) | -20.4% (0.0276) |
| BMI>25 | ↑ | Infection | Septicemia & Disseminated Infections; Infectious & Parasitic Diseases Including HIV W O.R. Procedure; Kidney & Urinary Tract Infections; Cystic Fibrosis; COPD; Respiratory Infections or Pneumonia | 1.444 (0.193) | 16.2% (0.3051) | 14.4% (521/3609) | 13.5% (318/2355) | 1.081 (0.083) | -17.2% (0.0233) |
| BMI>30 | ↑ | Major Hematologic/ Immunologic Diag | Acute Leukemia; Lymphatic & Other Malignancies & Neoplasms Of Uncertain Behavior; Other Anemia & Disorders Of Blood & Blood Forming Organs; Lymphoma; Major Hematologic/Immunologic Diag Exc Sickle Cell Crisis & Coagul | 1.042 (0.336) | -23.6% (0.2881) | 13.5% (65/483) | 9.1% (43/475) | 1.562 (0.325) | 18.6% (0.4184) |
| BMI 25-30 | ↑ | Major Hematologic/ Immunologic Diag | Acute Leukemia; Lymphatic & Other Malignancies & Neoplasms Of Uncertain Behavior; Other Anemia & Disorders Of Blood & Blood Forming Organs; Lymphoma; Major Hematologic/Immunologic Diag Exc Sickle Cell Crisis & Coagul | 1.367 (0.348) | 16.5% (0.6274) | 11.2% (50/445) | 9.1% (43/475) | 1.272 (0.279) | 13.4% (0.5709) |
| BMI>25 | ↑ | Major Hematologic/ Immunologic Diag | Acute Leukemia; Lymphatic & Other Malignancies & Neoplasms Of Uncertain Behavior; Other Anemia & Disorders Of Blood & Blood Forming Organs; Lymphoma; Major Hematologic/Immunologic Diag Exc Sickle Cell Crisis & Coagul | 1.243 (0.321) | -0.8% (0.8788) | 12.4% (115/928) | 9.1% (43/475) | 1.421 (0.268) | 15.2% (0.4590) |
| BMI>30 | ↑ | Leukemia | Admission and history of leukemia | 1.713 (0.486) | 26.1% (0.4849) | 16.9% (112/664) | 10.3% (73/710) | 1.770 (0.286) | 34.9% (0.0691) |
| **BMI 25-30** | ↑ | ***Leukemia** | **Admission and history of leukemia** | **2.172 (0.555)** | **88.7% (0.0189)** | **17.5% (120/685)** | **10.3% (73/710)** | **1.853 (0.295)** | **68.8% (0.0013)** |
| **BMI>25** | ↑ | **†Leukemia** | **Admission and history of leukemia** | **1.482 (0.475)** | **36.1% (0.4157)** | **17.2% (232/1349)** | **6.1% (4/66)** | **3.219 (1.677)** | **222.0% (0.0260)** |
| BMI>30 | ↑ | Liver Transplant | Liver Transplant | 1.643 (0.298) | 20.9% (0.3438) | 63.1% (77/122) | 44.6% (33/74) | 2.126 (0.637) | 61.9% (0.1101) |
| BMI 25-30 | ↑ | Liver Transplant | Liver Transplant | 1.226 (0.230) | 4.7% (0.8778) | 42.4% (42/99) | 44.6% (33/74) | 0.915 (0.284) | -18.5% (0.5123) |
| BMI>25 | ↑ | Liver Transplant | Liver Transplant | 1.608 (0.329) | 28.7% (0.2736) | 53.8% (119/221) | 44.6% (33/74) | 1.449 (0.391) | 17.7% (0.5495) |

© 2022 Liu K et al. *JAMA Network Open.*

| Target factor | Effect change in meta | Class of moderator | Detail of moderator | Subgroup model | | Subgroup analysis | | | |
|---|---|---|---|---|---|---|---|---|---|
| | | | | OR (se) | OR change (p-value) | Risk in exposed group | Risk in unexposed group | OR (se) | OR change (p-value) |
| BMI>30 | ↑ | Cerebrovascular | Head Trauma; CVA & Precerebral Occulusion w/ Infarct; Intracranial Hemorrhage | 1.380 (0.401) | 1.2% (0.9228) | 6.8% (34/497) | 5.7% (25/441) | 1.222 (0.332) | -7.4% (0.7778) |
| BMI 25-30 | ↑ | Cerebrovascular | Head Trauma; CVA & Precerebral Occulusion w/ Infarct; Intracranial Hemorrhage | 1.535 (0.435) | 31.4% (0.4190) | 7.4% (34/460) | 5.7% (25/441) | 1.328 (0.362) | 18.4% (0.5378) |
| BMI>25 | ↑ | Cerebrovascular | Head Trauma; CVA & Precerebral Occulusion w/ Infarct; Intracranial Hemorrhage | 1.614 (0.465) | 29.9% (0.4266) | 7.1% (68/957) | 5.7% (25/441) | 1.273 (0.307) | 3.0% (0.9027) |
| BMI>30 | ↓ | Uterine & Adnexa Procedures | Uterine & Adnexa Procedures; Pelvic Evisceration, Radical Hysterectectomy & Radical GYN Procs; Other Female Reproductive System & Related Procedures | 1.373 (0.479) | 0.7% (0.8863) | 7.9% (53/670) | 5.5% (16/293) | 1.487 (0.438) | 12.4% (0.6931) |
| **BMI 25-30** | ↓ | **Uterine & Adnexa Procedures** | **Uterine & Adnexa Procedures; Pelvic Evisceration, Radical Hysterectectomy & Radical GYN Procs; Other Female Reproductive System & Related Procedures** | **0.773 (0.199)** | **-34.2% (0.0746)** | **5.3% (16/303)** | **5.5% (16/293)** | **0.965 (0.351)** | **-14.3% (0.6728)** |
| BMI>25 | ↓ | Uterine & Adnexa Procedures | Uterine & Adnexa Procedures; Pelvic Evisceration, Radical Hysterectectomy & Radical GYN Procs; Other Female Reproductive System & Related Procedures | 1.309 (0.515) | 5.7% (0.9770) | 7.1% (69/973) | 5.5% (16/293) | 1.321 (0.378) | 6.7% (0.8218) |
| **Pulse>100** | ↑ | ***Liver disease** | **Alcoholic Liver Disease; Malignancy of Hepatobiliary System & Pancreas; Malignancy of Hepatobiliary System & Pancreas; Hepatic Coma & Other Major Acute Liver Disorders** | **2.942 (0.936)** | **113.1% (0.0313)** | **17.6% (29/165)** | **8.0% (72/898)** | **2.446 (0.584)** | **65.7% (0.0368)** |
| Pulse 80-100 | ↑ | Liver disease | Alcoholic Liver Disease; Malignancy of Hepatobiliary System & Pancreas; Malignancy of Hepatobiliary System & Pancreas; Hepatic Coma & Other Major Acute Liver Disorders | 1.290 (0.313) | 16.5% (0.6116) | 9.8% (57/582) | 8.0% (72/898) | 1.246 (0.232) | 5.4% (0.7776) |
| Pulse>80 or <50 | ↑ | Liver disease | Alcoholic Liver Disease; Malignancy of Hepatobiliary System & Pancreas; Malignancy of Hepatobiliary System & Pancreas; Hepatic Coma & Other Major Acute Liver Disorders | 1.906 (0.464) | 62.0% (0.0648) | 11.9% (90/757) | 8.0% (72/898) | 1.548 (0.258) | 23.3% (0.2127) |
| Pulse>100 | ↑ | Cerebrovascular | Extracranial Vascular Procedures; Craniotomy Except for Trauma; Head Trauma | 1.476 (0.422) | 7.3% (0.9119) | 9.2% (12/130) | 5.6% (84/1487) | 1.699 (0.549) | 14.7% (0.6742) |
| Pulse 80-100 | ↑ | Cerebrovascular | Extracranial Vascular Procedures; Craniotomy Except for Trauma; Head Trauma | 0.916 (0.235) | -17.5% (0.3840) | 5.7% (35/617) | 5.6% (84/1487) | 1.004 (0.208) | -14.8% (0.4439) |
| Pulse>80 or <50 | ↑ | Cerebrovascular | Extracranial Vascular Procedures; Craniotomy Except for Trauma; Head Trauma | 0.962 (0.201) | -18.7% (0.2667) | 6.1% (47/773) | 5.6% (84/1487) | 1.081 (0.203) | -13.9% (0.4287) |
| **Pulse>100** | ↑ | ***Cerebrovascular** | **All Cerebrovascular admission** (contain insignificant admission) | **2.594 (0.768)** | **89.9% (0.0494)** | **13.7% (31/226)** | **5.5% (134/2425)** | **2.718 (0.578)** | **87.1% (0.0038)** |

© 2022 Liu K et al. *JAMA Network Open.*

| Target factor | Effect change in meta | Class of moderator | Detail of moderator | Subgroup model | | Subgroup analysis | | | |
|---|---|---|---|---|---|---|---|---|---|
| | | | | OR (se) | OR change (p-value) | Risk in exposed group | Risk in unexposed group | OR (se) | OR change (p-value) |
| Pulse 80-100 | ↑ | Cerebrovascular | All Cerebrovascular admission (contain insignificant admission) | 1.199 (0.242) | 7.7% (0.7773) | 6.6% (69/1053) | 5.5% (134/2425) | 1.199 (0.183) | 2.4% (0.8807) |
| Pulse>80 or <50 | ↑ | Cerebrovascular | All Cerebrovascular admission (contain insignificant admission) | 1.393 (0.257) | 18.7% (0.4019) | 7.9% (105/1334) | 5.5% (134/2425) | 1.461 (0.197) | 17.4% (0.2423) |
| Pulse>100 | ↓ | Cardiac surgery | Cardiac Valve; Coronary Bypass; Other Cardiothoracic Procedures | 1.205 (0.402) | -11.0% (0.6174) | 29.1% (23/79) | 25.7% (378/1469) | 1.185 (0.302) | -26.0% (0.2421) |
| **Pulse 80-100** | **↓** | **†Cardiac surgery** | **Cardiac Valve; Coronary Bypass; Other Cardiothoracic Procedures** | **0.967 (0.186)** | **-13.1% (0.4139)** | **24.2% (157/650)** | **25.7% (378/1469)** | **0.919 (0.101)** | **-25.9% (0.0078)** |
| **Pulse>80 or <50** | **↓** | **†Cardiac surgery** | **Cardiac Valve; Coronary Bypass; Other Cardiothoracic Procedures** | **1.001 (0.182)** | **-14.7% (0.3434)** | **24.8% (183/737)** | **25.7% (378/1469)** | **0.953 (0.099)** | **-28.4% (0.0018)** |
| **Pulse>100** | **↓** | **†Orthopedic Surgery** | **Joint Replacement; Amputation of Lower Limb Except Toes; Knee & Lower Leg Procedures Except Foot; Tendon, Muscle & Other Soft Tissue Procedures** | **1.156 (0.374)** | **-19.0% (0.4464)** | **5.8% (24/411)** | **6.0% (146/2434)** | **0.972 (0.221)** | **-35.8% (0.0543)** |
| **Pulse 80-100** | **↓** | **†Orthopedic Surgery** | **Joint Replacement; Amputation of Lower Limb Except Toes; Knee & Lower Leg Procedures Except Foot; Tendon, Muscle & Other Soft Tissue Procedures** | **0.958 (0.176)** | **-15.0% (0.3257)** | **5.5% (109/1997)** | **6.0% (146/2434)** | **0.905 (0.118)** | **-24.8% (0.0325)** |
| **Pulse>80 or <50** | **↓** | **†Orthopedic Surgery** | **Joint Replacement; Amputation of Lower Limb Except Toes; Knee & Lower Leg Procedures Except Foot; Tendon, Muscle & Other Soft Tissue Procedures** | **0.946 (0.172)** | **-21.0% (0.1714)** | **5.5% (133/2426)** | **6.0% (146/2434)** | **0.909 (0.112)** | **-29.1% (0.0063)** |
| **Pulse>100** | **↓** | **#Infection** | **COPD; Post-Operative, Post-Traumatic, Other Device Infections; Cellulitis & Other Bacterial Skin Infections** | **0.796 (0.225)** | **-44.2% (0.0286)** | **11.0% (37/337)** | **10.4% (158/1514)** | **1.058 (0.205)** | **-30.2% (0.0684)** |
| **Pulse 80-100** | **↓** | ***Infection** | **COPD; Post-Operative, Post-Traumatic, Other Device Infections; Cellulitis & Other Bacterial Skin Infections** | **0.738 (0.151)** | **-35.1% (0.0302)** | **9.7% (118/1219)** | **10.4% (158/1514)** | **0.920 (0.118)** | **-23.1% (0.0457)** |
| **Pulse>80 or <50** | **↓** | ***Infection** | **COPD; Post-Operative, Post-Traumatic, Other Device Infections; Cellulitis & Other Bacterial Skin Infections** | **0.799 (0.168)** | **-33.5% (0.0395)** | **10.1% (158/1567)** | **10.4% (158/1514)** | **0.962 (0.114)** | **-24.6% (0.0203)** |
| **Vancomycin** | **↑** | ***Gastrointestinal surgery** | **Peritoneal Adhesiolysis; Major Small & Large Bowel Procedures; Anal Procedures; Malfunction, Reaction & Complication Of G.I. Device Or Procedure; Major Stomach, Esophageal & Duodenal Procedures** | **4.515 (1.548)** | **203.4% (0.0027)** | **29.1% (57/196)** | **11.7% (215/1843)** | **3.105 (0.538)** | **61.3% (0.0064)** |
| **Vancomycin** | **↑** | **Skin Graft** | **Skin Graft for Skin & Subcutaneous Tissue Diagnoses; Full Thickness Burns W Skin Graft** | **3.008 (0.980)** | **98.6% (0.0556)** | **21.9% (23/105)** | **10.8% (48/446)** | **2.326 (0.654)** | **21.1% (0.4980)** |

© 2022 Liu K et al. *JAMA Network Open.*

| Target factor | Effect change in meta | Class of moderator | Detail of moderator | Subgroup model | | Subgroup analysis | | | |
|---|---|---|---|---|---|---|---|---|---|
| | | | | OR (se) | OR change (p-value) | Risk in exposed group | Risk in unexposed group | OR (se) | OR change (p-value) |
| Vancomycin | ↑ | *Orthopedic Surgery | Musculoskeletal & Other Procedures For Multiple Significant Trauma; Hip & Femur Procedures Except Joint Replacement; Amputation of Lower Limb Except Toes; Shoulder, Upper Arm & Forearm Procedures; Knee & Lower Leg Procedures Except Foot | 2.874 (0.719) | 90.4% (0.0196) | 12.4% (78/630) | 5.4% (116/2148) | 2.475 (0.381) | 29.6% (0.0975) |
| Vancomycin | ↑ | *Infection | Post-Op,Post-Trauma, Other Device Infections; Infectious & Parasitic Diseases | 3.761 (1.033) | 151.3% (0.0005) | 22.4% (244/1087) | 11.7% (74/632) | 2.183 (0.313) | 18.3% (0.2513) |
| Vancomycin | ↓ | †Cardiac Procedure or Device | Cardiac Defibrillator & Heart Assist Anomaly; Cardiac Valve; Coronary Bypass; Other Cardiothoracic Procedures; Perm Cardiac Pacemaker Implant w/o AMI, Heart Failure or Shock | 1.325 (0.225) | -15.5% (0.3036) | 20.8% (260/1250) | 23.5% (357/1520) | 0.856 (0.079) | -56.1% (0.0000) |
| Vancomycin | ↓ | †Joint Replacement | Hip Joint Replacement; Knee Joint Replacement | 1.319 (0.350) | -15.1% (0.4411) | 4.7% (94/1999) | 6.5% (93/1429) | 0.709 (0.107) | -66.6% (0.0000) |

© 2022 Liu K et al. *JAMA Network Open.*

**eTable 17.** Significant Interactions Between 6 Important Predictors and Disease in Meta-Regression (Top-5 Mainly) and Their Verification

Direction of effect change in meta is based on result of meta-regression in general patients. OR change is calculated by the OR of target predictor when moderators is happened in controlled population dividing by the OR of target predictor in remaining patients of controlled population and subtract 100%. In subgroup analysis, when target factor is age, exposed group contains patients with age>65, unexposed group contains patients with age<45; when target factor is BMI or pulse, unexposed group contains patients with BMI:18.5-25 and pulse: 50-80 respectively; when target factor is lab test, exposed group contains patients with abnormal result, unexposed group contains patients with normal result. Moderators significantly ($p \leqslant 0.05$) verified by subgroup model, subgroup analysis and both are marked with #, †, and * respectively. Significant interactions or interactions we suggest need to be concerned are shown in bold.

| Target factor | Effect change in meta | Moderator | Controlled population | % of sample account for | Subgroup model | | Subgroup analysis | | | |
|---|---|---|---|---|---|---|---|---|---|---|
| | | | | | OR (se) | OR change (p-value) | Risk in exposed group | Risk in unexposed group | OR (se) | OR change (p-value) |
| **Age** | ↑ | **Aldesleukin** | **#General** | **-** | **2.059 (0.307)** | **87.1% (0.0000)** | **58.8% (10/17)** | **52.6% (30/57)** | **1.286 (0.720)** | **-8.2% (0.8790)** |
| | | | **#Chemotherapy** | **98.50%** | **2.032 (0.303)** | **43.5% (0.0512)** | **58.8% (10/17)** | **52.6% (30/57)** | **1.286 (0.720)** | **-68.9% (0.0633)** |
| **Age** | ↑ | **Aminocaproic acid** | ***General** | **-** | **1.472 (0.148)** | **34.4% (0.0040)** | **27.0% (199/737)** | **12.1% (26/215)** | **2.689 (0.605)** | **106.3% (0.0015)** |
| | | | **Major cardiac surgery** | **76.80%** | **1.684 (0.215)** | **45.2% (0.0602)** | **29.4% (169/574)** | **10.1% (15/149)** | **3.728 (1.071)** | **78.8% (0.1793)** |
| **Age** | ↑ | **Tranexamic acid** | General | - | 1.016 (0.134) | -7.5% (0.5091) | 11.6% (92/792) | 6.8% (14/205) | 1.793 (0.535) | 30.3% (0.3777) |
| | | | Joint replacement | 54.70% | 1.014 (0.204) | 0.8% (0.9868) | 4.0% (17/428) | 1.1% (1/89) | 3.640 (3.770) | 26.7% (0.8328) |
| | | | **Major cardiac surgery** | **25.90%** | **1.020 (0.176)** | **-42.3% (0.0068)** | **26.9% (58/216)** | **12.8% (6/47)** | **2.508 (1.162)** | **-20.2% (0.6644)** |
| **Age** | ↑ | **Prochlorperazine** | **†General** | **-** | **1.256 (0.089)** | **14.7% (0.0621)** | **19.7% (291/1474)** | **8.4% (51/607)** | **2.682 (0.430)** | **108.8% (0.0000)** |
| | | | Major cardiac surgery | 46.40% | 1.414 (0.158) | -36.6% (0.0169) | 29.9% (215/719) | 12.1% (20/165) | 3.093 (0.780) | 2.9% (0.9520) |
| | | | Respiratory & Chest Procedures | 9.80% | 1.164 (0.160) | 5.5% (0.7886) | 7.9% (11/140) | 4.2% (3/71) | 1.933 (1.292) | 107.5% (0.3555) |

© 2022 Liu K et al. *JAMA Network Open.*

| Target factor | Effect change in meta | Moderator | Controlled population | % of sample account for | Subgroup model | | Subgroup analysis | | | |
|---|---|---|---|---|---|---|---|---|---|---|
| | | | | | OR (se) | OR change (p-value) | Risk in exposed group | Risk in unexposed group | OR (se) | OR change (p-value) |
| **Age** | ↑ | **Protamine sulfate** | **#General** | - | **1.326 (0.092)** | **21.2% (0.0073)** | **17.0% (276/1624)** | **11.4% (49/429)** | **1.588 (0.263)** | **20.6% (0.2690)** |
| | | | Major cardiac surgery | 49.50% | 1.436 (0.167) | -26.1% (0.1785) | 25.2% (182/722) | 10.6% (19/179) | 2.838 (0.730) | -31.4% (0.4342) |
| | | | PCI w/o AMI | 14.60% | 0.969 (0.205) | -3.1% (0.8675) | 6.9% (23/331) | 6.7% (3/45) | 1.045 (0.664) | -34.9% (0.6627) |
| **Age** | ↓ | **Rifaximin** | ***General** | - | **0.759 (0.085)** | **-31.5% (0.0008)** | **12.0% (26/216)** | **16.3% (52/320)** | **0.705 (0.182)** | **-49.8% (0.0081)** |
| | | | **Liver disease** | **32.10%** | **0.831 (0.120)** | **-8.1% (0.6798)** | **4.9% (3/61)** | **11.8% (11/93)** | **0.386 (0.260)** | **-52.2% (0.3690)** |
| | | | **Liver surgery** | **10.70%** | **0.850 (0.125)** | **-22.6% (0.1916)** | **33.3% (6/18)** | **30.3% (10/33)** | **1.150 (0.721)** | **46.4% (0.5820)** |
| | | | **Liver disease or surgery** | **42.90%** | **0.899 (0.128)** | **-17.1% (0.2817)** | **11.4% (9/79)** | **16.7% (21/126)** | **0.643 (0.275)** | **-34.1% (0.3912)** |
| **Age** | ↓ | **Levofloxacin** | ***General** | - | **1.007 (0.024)** | **-12.1% (0.0000)** | **12.0% (663/5546)** | **12.5% (634/5080)** | **0.952 (0.056)** | **-40.8% (0.0000)** |
| | | | **Infection** | **26.10%** | **0.904 (0.050)** | **-9.0% (0.2357)** | **11.6% (169/1460)** | **16.6% (241/1453)** | **0.658 (0.071)** | **-18.4% (0.2262)** |
| **Age** | ↓ | **Glucose** | ***General** | - | **1.074 (0.019)** | **-8.5% (0.0019)** | **13.1% (1364/10396)** | **10.8% (1193/11061)** | **1.249 (0.053)** | **-24.8% (0.0001)** |
| **Age** | ↓ | **Vancomycin** | ***General** | - | **1.001 (0.024)** | **-13.9% (0.0000)** | **13.5% (655/4844)** | **15.7% (654/4153)** | **0.837 (0.050)** | **-49.6% (0.0000)** |
| | | | **Systemic infection** | **15.50%** | **0.899 (0.068)** | **-10.9% (0.3522)** | **17.9% (108/603)** | **21.2% (165/780)** | **0.813 (0.112)** | **-22.5% (0.2501)** |
| | | | **Joint replacement** | **12.00%** | **0.938 (0.120)** | **-14.0% (0.4297)** | **4.8% (37/770)** | **1.8% (3/169)** | **2.793 (1.694)** | **-9.9% (0.8975)** |
| **Age** | ↓ | **Tazobactam** | ***General** | - | **0.976 (0.026)** | **-15.8% (0.0000)** | **15.1% (515/3407)** | **17.0% (716/4207)** | **0.868 (0.055)** | **-50.7% (0.0000)** |
| | | | ***Systemic infection** | **19.20%** | **0.871 (0.062)** | **-19.0% (0.0482)** | **18.0% (121/674)** | **22.7% (188/829)** | **0.746 (0.097)** | **-41.6% (0.0226)** |
| | | | **Other common infection** | **13.40%** | **0.895 (0.083)** | **-4.7% (0.7104)** | **11.4% (50/438)** | **18.0% (122/679)** | **0.588 (0.106)** | **14.2% (0.6158)** |
| | | | **#Infection** | **32.60%** | **0.889 (0.046)** | **-15.0% (0.0439)** | **15.4% (171/1112)** | **20.6% (310/1508)** | **0.702 (0.074)** | **-11.2% (0.4853)** |
| **Serum calcium** | ↑ | **Aminocaproic acid** | ***General** | - | **3.687 (0.692)** | **218.2% (0.0000)** | **32.6% (298/914)** | **11.1% (112/1007)** | **3.866 (0.474)** | **182.3% (0.0000)** |
| | | | **Major cardiac surgery** | **76.80%** | **4.060 (0.888)** | **-53.9% (0.0267)** | **33.9% (250/738)** | **10.7% (79/737)** | **4.267 (0.607)** | **27.0% (0.2961)** |
| **Serum calcium** | ↑ | **Prochlorperazine** | ***General** | - | **3.489 (0.490)** | **209.4% (0.0000)** | **25.2% (406/1610)** | **8.6% (194/2259)** | **3.589 (0.339)** | **167.2% (0.0000)** |

© 2022 Liu K et al. *JAMA Network Open.*

| Target factor | Effect change in meta | Moderator | Controlled population | % of sample account for | Subgroup model | | Subgroup analysis | | | |
|---|---|---|---|---|---|---|---|---|---|---|
| | | | | | OR (se) | OR change (p-value) | Risk in exposed group | Risk in unexposed group | OR (se) | OR change (p-value) |
| | | | ***Major cardiac surgery** | **46.40%** | **5.654 (1.160)** | **94.1% (0.0447)** | **35.8% (293/819)** | **11.0% (107/975)** | **4.519 (0.568)** | **119.2% (0.0031)** |
| | | | Respiratory & Chest Procedures | 9.80% | 1.051 (0.298) | 39.7% (0.3633) | 7.9% (12/152) | 6.2% (14/227) | 1.304 (0.532) | 61.3% (0.3826) |
| **Serum calcium** | ↑ | **Protamine sulfate** | ***General** | - | **3.258 (0.492)** | **189.4% (0.0000)** | **25.1% (351/1399)** | **8.4% (193/2285)** | **3.630 (0.353)** | **166.9% (0.0000)** |
| | | | **†Major cardiac surgery** | **49.50%** | **5.292 (1.193)** | **84.6% (0.0656)** | **33.2% (257/775)** | **8.7% (91/1048)** | **5.218 (0.697)** | **254.2% (0.0000)** |
| | | | PCI w/o AMI | 14.60% | 1.481 (0.390) | 48.4% (0.2994) | 9.2% (14/153) | 5.4% (21/386) | 1.751 (0.629) | 83.0% (0.2365) |
| **Serum calcium** | ↑ | **Atropine** | ***General** | - | **2.748 (0.332)** | **143.2% (0.0000)** | **23.5% (458/1946)** | **9.2% (299/3260)** | **3.048 (0.246)** | **127.0% (0.0000)** |
| | | | ***Major cardiac surgery** | **39.10%** | **5.925 (1.131)** | **314.5% (0.0000)** | **35.9% (317/882)** | **10.7% (124/1155)** | **4.665 (0.551)** | **195.1% (0.0180)** |
| | | | Cardiac Catheterization | 13.10% | 0.841 (0.213) | -8.1% (0.7966) | 5.9% (9/152) | 5.3% (28/530) | 1.128 (0.445) | 14.5% (0.7588) |
| **Serum calcium** | ↑ | **Tranexamic acid** | ***General** | - | **3.933 (1.002)** | **229.4% (0.0000)** | **12.6% (85/674)** | **6.0% (80/1337)** | **2.268 (0.371)** | **56.4% (0.0069)** |
| | | | Joint replacement | 54.70% | 0.968 (0.249) | 39.8% (0.3594) | 2.6% (12/457) | 3.0% (19/643) | 0.886 (0.331) | 38.8% (0.4234) |
| | | | ***Major cardiac surgery** | **25.90%** | **9.344 (2.589)** | **122.8% (0.0295)** | **47.3% (62/131)** | **9.5% (37/389)** | **8.548 (2.102)** | **280.1% (0.0000)** |
| **Serum calcium** | ↓ | **Oxycodone** | **†General** | - | **1.232 (0.049)** | **2.9% (0.6636)** | **10.6% (1875/17622)** | **8.1% (2343/29089)** | **1.359 (0.044)** | **-20.4% (0.0000)** |
| | | | ***Joint replacement and common orthopeadic surgery** | **12.90%** | **0.701 (0.123)** | **-60.8% (0.0021)** | **4.7% (143/3024)** | **5.7% (172/3009)** | **0.819 (0.095)** | **-64.6% (0.0048)** |
| **Serum calcium** | ↓ | **Spironolactone** | ***General** | - | **0.944 (0.120)** | **-23.7% (0.0379)** | **16.7% (361/2160)** | **15.0% (477/3184)** | **1.139 (0.087)** | **-23.5% (0.0009)** |
| | | | Liver disease | 13.60% | 0.405 (0.114) | -48.2% (0.0999) | 8.5% (39/460) | 11.2% (30/269) | 0.738 (0.189) | -25.3% (0.4361) |
| | | | Heart fail, cardiac catheterization exc ischemic heart disease, cardiac defibrillator & heart assist | 12.00% | 1.207 (0.381) | 0.4% (0.9888) | 24.7% (23/93) | 19.1% (105/549) | 1.389 (0.366) | 18.0% (0.5999) |
| **Serum calcium** | ↓ | **Fondaparinux** | ***General** | - | **0.711 (0.164)** | **-43.0% (0.0112)** | **5.7% (96/1697)** | **6.1% (96/1577)** | **0.925 (0.138)** | **-38.8% (0.0012)** |
| | | | **#Joint replacement** | **76.70%** | **0.580 (0.141)** | **-48.8% (0.0547)** | **4.1% (59/1432)** | **5.6% (60/1080)** | **0.731 (0.137)** | **-22.7% (0.4132)** |

© 2022 Liu K et al. *JAMA Network Open.*

| Target factor | Effect change in meta | Moderator | Controlled population | % of sample account for | Subgroup model | | Subgroup analysis | | | |
|---|---|---|---|---|---|---|---|---|---|---|
| | | | | | OR (se) | OR change (p-value) | Risk in exposed group | Risk in unexposed group | OR (se) | OR change (p-value) |
| **Serum calcium** | ↓ | **Rifaximin** | ***General** | **-** | **0.729 (0.174)** | **-41.1% (0.0169)** | **14.2% (148/1041)** | **14.2% (106/746)** | **1.001 (0.137)** | **-31.8% (0.0061)** |
| | | | Liver disease | 32.10% | 0.562 (0.160) | -1.0% (0.9891) | 6.8% (23/337) | 9.7% (23/237) | 0.682 (0.210) | -30.5% (0.3472) |
| | | | Liver surgery | 10.70% | 0.940 (0.155) | 28.9% (0.3470) | 32.8% (45/137) | 38.2% (21/55) | 0.792 (0.263) | -22.2% (0.5026) |
| | | | Liver disease or surgery | 42.90% | 0.612 (0.156) | -10.0% (0.7623) | 14.3% (68/474) | 15.1% (44/292) | 0.944 (0.198) | -5.9% (0.8087) |
| **Serum calcium** | ↓ | **Aldesleukin** | ***General** | **-** | **0.132 (0.033)** | **-89.5% (0.0000)** | **32.2% (47/146)** | **93.0% (107/115)** | **0.035 (0.014)** | **-97.6% (0.0000)** |
| | | | ***Chemotherapy** | **98.50%** | **0.125 (0.026)** | **-89.6% (0.0000)** | **32.2% (47/146)** | **96.4% (107/111)** | **0.018 (0.010)** | **-98.9% (0.0000)** |
| **Blood glucose** | ↑ | **Insulin,aspart, human/rdna** | General | - | 1.395 (0.073) | 2.6% (0.7325) | 14.9% (1041/6977) | 11.9% (1926/16182) | 1.298 (0.054) | -11.4% (0.0571) |
| | | | **Major cardiac surgery** | **8.30%** | **1.522 (0.467)** | **46.6% (0.3463)** | **32.8% (62/189)** | **17.1% (298/1743)** | **2.367 (0.396)** | **-3.7% (0.9548)** |
| **Blood glucose** | ↑ | **Glucose** | **#General** | **-** | **1.509 (0.077)** | **18.6% (0.0330)** | **16.6% (1057/6366)** | **11.5% (3716/32426)** | **1.538 (0.058)** | **-5.5% (0.3772)** |
| **Blood glucose** | ↑ | **Fentanyl** | ***General** | **-** | **1.498 (0.065)** | **20.6% (0.0395)** | **13.7% (1180/8616)** | **8.7% (4315/49729)** | **1.670 (0.059)** | **19.9% (0.0087)** |
| **Blood glucose** | ↑ | **Lactate** | **†General** | **-** | **1.564 (0.110)** | **14.6% (0.1019)** | **13.5% (573/4254)** | **7.8% (2076/26702)** | **1.847 (0.093)** | **27.5% (0.0001)** |
| | | | Joint replacement and common orthopeadic surgery | 19.80% | 2.441 (0.585) | 71.2% (0.1160) | 10.3% (54/524) | 4.6% (259/5597) | 2.368 (0.372) | 15.1% (0.7501) |
| **Blood glucose** | ↑ | **Benzoic acid** | General | - | 1.491 (0.096) | 8.9% (0.3001) | 19.4% (692/3558) | 13.8% (2383/17268) | 1.508 (0.072) | -5.8% (0.3346) |
| | | | Infection | 13.00% | 1.258 (0.280) | -6.4% (0.7640) | 20.3% (118/581) | 16.5% (349/2118) | 1.292 (0.153) | -22.2% (0.0908) |
| | | | **#Heart fail, cardiac catheterization exc ischemic heart disease, cardiac defibrillator & heart assist** | **8.80%** | **1.722 (0.393)** | **96.1% (0.0452)** | **24.3% (75/309)** | **14.5% (222/1527)** | **1.884 (0.285)** | **55.3% (0.3247)** |
| | | | **Major cardiac surgery** | **8.40%** | **1.755 (0.512)** | **101.9% (0.0629)** | **31.3% (50/160)** | **19.5% (310/1591)** | **1.878 (0.342)** | **-11.8% (0.7224)** |
| **Blood glucose** | ↑ | **Insulin regular,human buffered** | General | - | 1.567 (0.179) | 15.1% (0.2549) | 18.3% (391/2136) | 16.7% (934/5578) | 1.114 (0.074) | -29.9% (0.0000) |
| | | | **Major cardiac surgery** | **26.30%** | **1.262 (0.357)** | **-18.0% (0.4621)** | **30.2% (55/182)** | **20.8% (384/1847)** | **1.650 (0.283)** | **-65.2% (0.1080)** |

© 2022 Liu K et al. *JAMA Network Open.*

| Target factor | Effect change in meta | Moderator | Controlled population | % of sample account for | Subgroup model | | Subgroup analysis | | | |
|---|---|---|---|---|---|---|---|---|---|---|
| | | | | | OR (se) | OR change (p-value) | Risk in exposed group | Risk in unexposed group | OR (se) | OR change (p-value) |
| **Blood glucose** | ↓ | **Insulin, isophane** | ***General** | **-** | **0.988 (0.193)** | **-31.4% (0.0463)** | **12.2% (138/1134)** | **11.9% (133/1114)** | **1.022 (0.132)** | **-36.8% (0.0006)** |
| | | | **Diabetes** | **8.40%** | **0.589 (0.065)** | **-32.0% (0.1319)** | **2.8% (4/141)** | **8.3% (4/48)** | **0.321 (0.234)** | **-74.2% (0.1395)** |
| | | | ***Infection** | **17.80%** | **0.579 (0.144)** | **-57.0% (0.0011)** | **11.3% (24/212)** | **14.8% (28/189)** | **0.734 (0.219)** | **-55.3% (0.0088)** |
| | | | **#Liver disease or surgery** | **7.30%** | **0.956 (0.212)** | **-56.2% (0.0112)** | **13.3% (10/75)** | **10.0% (9/90)** | **1.385 (0.677)** | **-20.3% (0.6559)** |
| Blood glucose | ↓ | Tazobactam | General | - | 1.294 (0.098) | -12.8% (0.1277) | 21.3% (524/2458) | 15.6% (1752/11203) | 1.462 (0.081) | -8.3% (0.1948) |
| | | | Systemic infection | 19.20% | 0.971 (0.201) | -40.1% (0.2154) | 25.0% (127/508) | 19.6% (414/2115) | 1.370 (0.159) | -16.3% (0.4060) |
| | | | Other common infection | 13.40% | 1.471 (0.345) | 27.5% (0.4712) | 21.7% (81/374) | 13.0% (189/1459) | 1.858 (0.275) | 19.9% (0.4024) |
| | | | Infection | 32.60% | 1.157 (0.177) | -22.0% (0.2919) | 23.6% (208/882) | 16.9% (603/3574) | 1.521 (0.138) | -1.7% (0.9085) |
| Blood glucose | ↓ | Aluminium hydroxide | General | - | 1.414 (0.101) | -0.3% (0.9553) | 13.6% (451/3321) | 9.6% (1970/20618) | 1.488 (0.083) | -9.9% (0.1190) |
| | | | Joint replacement | 7.80% | 2.121 (0.773) | -27.4% (0.5779) | 11.2% (15/134) | 4.2% (73/1744) | 2.885 (0.863) | 35.2% (0.4748) |
| **Blood glucose** | ↓ | **Paracetamol** | General | - | 1.408 (0.057) | -3.3% (0.7408) | 12.3% (1098/8921) | 8.4% (4190/49991) | 1.534 (0.055) | -9.8% (0.1214) |
| | | | Infection | 12.70% | 1.310 (0.163) | -6.2% (0.8757) | 16.5% (228/1385) | 11.7% (715/6093) | 1.482 (0.123) | -14.1% (0.3510) |
| | | | **Joint replacement and common orthopeadic surgery** | **10.30%** | **2.691 (0.613)** | **142.7% (0.0081)** | **11.1% (60/542)** | **4.7% (260/5541)** | **2.528 (0.382)** | **29.1% (0.6751)** |
| Blood glucose | ↓ | Vancomycin | General | - | 1.305 (0.113) | -13.0% (0.1404) | 19.7% (493/2500) | 13.5% (1910/14169) | 1.577 (0.088) | -3.0% (0.6440) |
| | | | Systemic infection | 15.50% | 1.127 (0.232) | -27.1% (0.3896) | 23.7% (116/489) | 18.9% (395/2090) | 1.335 (0.160) | -23.9% (0.1809) |
| | | | Joint replacement | 12.00% | 1.738 (0.628) | -46.6% (0.2190) | 8.1% (11/136) | 4.5% (83/1863) | 1.887 (0.630) | -36.7% (0.2922) |
| **Blood glucose** | ↓ | **Acetaminophen** | **†General** | **-** | **1.385 (0.057)** | **-13.2% (0.2190)** | **12.4% (1257/10150)** | **8.5% (4788/56258)** | **1.519 (0.051)** | **-19.3% (0.0058)** |
| | | | Infection | 12.50% | 1.265 (0.150) | -30.0% (0.3072) | 16.5% (256/1555) | 11.9% (797/6715) | 1.463 (0.114) | -28.0% (0.0863) |
| | | | **Joint replacement and common orthopeadic surgery** | **9.40%** | **2.596 (0.582)** | **208.4% (0.0000)** | **11.4% (63/553)** | **4.8% (274/5668)** | **2.531 (0.373)** | **158.4% (0.3899)** |

© 2022 Liu K et al. *JAMA Network Open.*

| Target factor | Effect change in meta | Moderator | Controlled population | % of sample account for | Subgroup model | | Subgroup analysis | | | |
|---|---|---|---|---|---|---|---|---|---|---|
| | | | | | OR (se) | OR change (p-value) | Risk in exposed group | Risk in unexposed group | OR (se) | OR change (p-value) |
| **BMI>30** | ↑ | **Tazobactam** | General | - | 1.380 (0.128) | 2.8% (0.8126) | 20.4% (905/4426) | 14.8% (646/4365) | 1.480 (0.084) | 5.9% (0.4035) |
| | | | **†Systemic infection** | **19.30%** | **2.155 (0.460)** | **87.0% (0.0839)** | **25.2% (235/933)** | **19.2% (147/764)** | **1.413 (0.168)** | **55.7% (0.0421)** |
| | | | Other common infection | 13.40% | 1.291 (0.399) | -25.0% (0.4393) | 16.6% (85/512) | 14.9% (99/663) | 1.134 (0.183) | 8.5% (0.7247) |
| | | | **†Infection** | **32.70%** | **1.772 (0.316)** | **16.9% (0.5652)** | **22.1% (320/1445)** | **17.2% (246/1427)** | **1.366 (0.129)** | **35.6% (0.0497)** |
| BMI 25-30 | ↑ | Tazobactam | General | - | 1.060 (0.089) | -12.4% (0.1736) | 15.9% (561/3523) | 14.8% (646/4365) | 1.090 (0.068) | -11.0% (0.1221) |
| | | | Systemic infection | 18.60% | 1.190 (0.261) | 1.3% (0.9571) | 18.0% (127/706) | 19.2% (147/764) | 0.921 (0.124) | -3.6% (0.8795) |
| | | | Other common infection | 13.10% | 1.243 (0.359) | 32.9% (0.4745) | 13.2% (49/371) | 14.9% (99/663) | 0.867 (0.163) | 32.1% (0.3347) |
| | | | Infection | 31.70% | 1.176 (0.223) | 1.2% (0.9535) | 16.3% (176/1077) | 17.2% (246/1427) | 0.938 (0.101) | 10.0% (0.5981) |
| BMI>25 | ↑ | Tazobactam | General | - | 1.193 (0.092) | -5.4% (0.5213) | 18.4% (1466/7949) | 14.8% (646/4365) | 1.302 (0.067) | -1.7% (0.7810) |
| | | | Systemic infection | 19.50% | 1.364 (0.263) | 12.0% (0.6932) | 22.1% (362/1639) | 19.2% (147/764) | 1.190 (0.130) | 28.4% (0.2076) |
| | | | Other common infection | 12.60% | 1.155 (0.295) | -8.7% (0.8219) | 15.2% (134/883) | 14.9% (99/663) | 1.019 (0.147) | 13.9% (0.5395) |
| | | | Infection | 32.10% | 1.310 (0.199) | 4.5% (0.8331) | 19.7% (496/2522) | 17.2% (246/1427) | 1.175 (0.101) | 24.2% (0.1270) |
| BMI>30 | ↑ | Benzimidazole | General | - | 1.404 (0.082) | 3.7% (0.6698) | 11.6% (1680/14514) | 8.9% (924/10349) | 1.335 (0.058) | 2.2% (0.7352) |
| BMI 25-30 | ↑ | Benzimidazole | General | - | 1.227 (0.076) | 8.9% (0.3265) | 9.8% (1032/10554) | 8.9% (924/10349) | 1.106 (0.053) | -3.8% (0.5809) |
| BMI>25 | ↑ | Benzimidazole | General | - | 1.292 (0.068) | 7.1% (0.3499) | 10.8% (2712/25068) | 8.9% (924/10349) | 1.237 (0.050) | -0.1% (0.9800) |
| **BMI>30** | ↑ | **Calcium gluconate** | ***General** | **-** | **2.178 (0.401)** | **63.7% (0.0112)** | **15.5% (250/1608)** | **9.7% (133/1375)** | **1.719 (0.196)** | **32.4% (0.0182)** |
| | | | Cerebrovascular | 22.30% | 2.070 (0.540) | 28.9% (0.4895) | 7.7% (30/389) | 4.7% (13/277) | 1.697 (0.580) | 54.1% (0.2918) |
| **BMI 25-30** | ↑ | **Calcium gluconate** | **†General** | **-** | **1.552 (0.308)** | **35.5% (0.1486)** | **13.5% (177/1315)** | **9.7% (133/1375)** | **1.452 (0.177)** | **31.9% (0.0295)** |

© 2022 Liu K et al. *JAMA Network Open.*

| Target factor | Effect change in meta | Moderator | Controlled population | % of sample account for | Subgroup model | | Subgroup analysis | | | |
|---|---|---|---|---|---|---|---|---|---|---|
| | | | | | OR (se) | OR change (p-value) | Risk in exposed group | Risk in unexposed group | OR (se) | OR change (p-value) |
| | | | Cerebrovascular | 23.90% | 1.304 (0.311) | -20.0% (0.5279) | 6.0% (22/366) | 4.7% (13/277) | 1.299 (0.467) | -13.1% (0.7381) |
| **BMI>25** | ↑ | **Calcium gluconate** | ***General** | **-** | **1.828 (0.275)** | **50.7% (0.0086)** | **14.6% (427/2923)** | **9.7% (133/1375)** | **1.598 (0.168)** | **31.6% (0.0123)** |
| | | | Cerebrovascular | 24.00% | 1.766 (0.526) | 2.2% (0.9815) | 6.9% (52/755) | 4.7% (13/277) | 1.502 (0.478) | 16.6% (0.6822) |
| **BMI>30** | ↑ | **Insulin regular,human buffered** | General | - | 1.648 (0.200) | 24.6% (0.1038) | 19.7% (629/3193) | 15.6% (271/1742) | 1.332 (0.106) | 3.9% (0.6606) |
| | | | **#Major cardiac surgery** | **26.50%** | **2.215 (0.562)** | **101.3% (0.0140)** | **24.7% (226/914)** | **17.4% (69/396)** | **1.557 (0.238)** | **1.2% (0.9808)** |
| BMI 25-30 | ↑ | Insulin regular,human buffered | General | - | 1.418 (0.210) | 22.4% (0.2152) | 17.7% (369/2080) | 15.6% (271/1742) | 1.171 (0.102) | 6.5% (0.5077) |
| | | | Major cardiac surgery | 28.8% | 1.433 (0.362) | 29.8% (0.3860) | 19.9% (140/703) | 17.4% (69/396) | 1.178 (0.192) | -12.6% (0.7936) |
| **BMI>25** | ↑ | **Insulin regular,human buffered** | General | - | 1.491 (0.174) | 22.2% (0.1120) | 18.9% (998/5273) | 15.6% (271/1742) | 1.267 (0.095) | 5.3% (0.5246) |
| | | | **#Major cardiac surgery** | **28.70%** | **2.039 (0.531)** | **77.8% (0.0544)** | **22.6% (366/1617)** | **17.4% (69/396)** | **1.387 (0.201)** | **-4.9% (0.9133)** |
| BMI>30 | ↑ | Levofloxacin | General | - | 1.482 (0.122) | 11.0% (0.3075) | 14.8% (949/6433) | 11.6% (681/5854) | 1.315 (0.071) | -6.2% (0.3366) |
| | | | Infection | 26.30% | 1.544 (0.281) | -2.1% (0.9585) | 16.6% (271/1628) | 14.0% (225/1606) | 1.226 (0.120) | 8.4% (0.5890) |
| BMI 25-30 | ↑ | Levofloxacin | General | - | 1.108 (0.095) | -10.1% (0.2808) | 12.1% (605/4980) | 11.6% (681/5854) | 1.050 (0.063) | -13.5% (0.0490) |
| | | | Infection | 25.40% | 1.195 (0.229) | -12.5% (0.6331) | 12.6% (144/1145) | 14.0% (225/1606) | 0.883 (0.101) | -7.0% (0.6765) |
| BMI>25 | ↑ | Levofloxacin | General | - | 1.238 (0.082) | -3.0% (0.7043) | 13.6% (1554/11413) | 11.6% (681/5854) | 1.197 (0.059) | -9.5% (0.1057) |
| | | | Infection | 25.40% | 1.280 (0.200) | 0.4% (0.9833) | 15.0% (415/2773) | 14.0% (225/1606) | 1.080 (0.097) | 2.2% (0.8736) |
| **BMI>30** | ↓ | **Rifaximin** | General | - | 1.559 (0.415) | 13.7% (0.7162) | 17.2% (118/688) | 12.4% (60/484) | 1.463 (0.250) | 11.1% (0.5443) |
| | | | **#Liver disease** | **31.60%** | **0.787 (0.181)** | **-50.9% (0.0482)** | **6.6% (14/212)** | **7.6% (12/158)** | **0.860 (0.351)** | **-54.9% (0.1215)** |
| | | | **Liver surgery** | **11.20%** | **1.271 (0.186)** | **-35.9% (0.1971)** | **44.7% (38/85)** | **26.1% (12/46)** | **2.291 (0.917)** | **40.6% (0.4562)** |
| | | | **Liver disease or surgery** | **42.70%** | **1.194 (0.272)** | **-42.1% (0.1093)** | **17.5% (52/297)** | **11.8% (24/204)** | **1.592 (0.423)** | **-9.6% (0.7525)** |
| BMI 25-30 | ↓ | Rifaximin | General | - | 1.261 | 7.5% | 13.0% | 12.4% | 1.053 | -6.5% |

© 2022 Liu K et al. *JAMA Network Open.*

| Target factor | Effect change in meta | Moderator | Controlled population | % of sample account for | Subgroup model | | Subgroup analysis | | | |
|---|---|---|---|---|---|---|---|---|---|---|
| | | | | | OR (se) | OR change (p-value) | Risk in exposed group | Risk in unexposed group | OR (se) | OR change (p-value) |
| | | | | | (0.309) | (0.8603) | (69/532) | (60/484) | (0.199) | (0.7266) |
| | | | Liver disease | 32.80% | 1.085 (0.233) | -7.1% (0.8206) | 9.7% (17/175) | 7.6% (12/158) | 1.309 (0.516) | 9.7% (0.8598) |
| | | | Liver surgery | 9.70% | 0.984 (0.135) | -16.1% (0.5781) | 26.4% (14/53) | 26.1% (12/46) | 1.017 (0.466) | 2.4% (0.9637) |
| | | | Liver disease or surgery | 42.50% | 1.001 (0.252) | -21.9% (0.4777) | 13.6% (31/228) | 11.8% (24/204) | 1.180 (0.343) | 5.2% (0.8837) |
| BMI>25 | ↓ | Rifaximin | General | - | 1.342 (0.323) | 6.8% (0.8653) | 15.3% (187/1220) | 12.4% (60/484) | 1.279 (0.204) | 3.6% (0.8293) |
| | | | Liver disease | 32.00% | 0.867 (0.260) | -36.1% (0.2088) | 8.0% (31/387) | 7.6% (12/158) | 1.059 (0.375) | -32.5% (0.3908) |
| | | | Liver surgery | 10.80% | 1.116 (0.166) | -39.1% (0.1034) | 37.7% (52/138) | 26.1% (12/46) | 1.713 (0.649) | 31.7% (0.5211) |
| | | | Liver disease or surgery | 42.80% | 1.064 (0.253) | -41.2% (0.1315) | 15.8% (83/525) | 11.8% (24/204) | 1.408 (0.349) | -2.5% (0.9329) |
| BMI>30 | ↓ | 1-(4-(4-chlorophenyl)-3-phenyl-2-butenyl) -pyrrolidine 1,5-naphthalenedisulfonate | General | - | 1.393 (0.069) | 1.5% (0.8586) | 11.3% (2365/21021) | 8.7% (1062/12202) | 1.330 (0.052) | 7.2% (0.3109) |
| BMI 25-30 | ↓ | 1-(4-(4-chlorophenyl)-3-phenyl-2-butenyl) -pyrrolidine 1,5-naphthalenedisulfonate | General | - | 1.172 (0.056) | -4.0% (0.7012) | 9.4% (1355/14362) | 8.7% (1062/12202) | 1.093 (0.047) | -7.0% (0.3195) |
| BMI>25 | ↓ | 1-(4-(4-chlorophenyl)-3-phenyl-2-butenyl) -pyrrolidine 1,5-naphthalenedisulfonate | General | - | 1.254 (0.055) | -2.8% (0.7431) | 10.5% (3720/35383) | 8.7% (1062/12202) | 1.232 (0.045) | 1.9% (0.7675) |
| BMI>30 | ↓ | Paracetamol | General | - | 1.350 (0.073) | -7.8% (0.4474) | 10.2% (2306/22545) | 8.1% (1216/15023) | 1.294 (0.048) | -11.6% (0.0864) |
| BMI 25-30 | ↓ | Paracetamol | General | - | 1.135 (0.056) | -17.6% (0.1203) | 8.8% (1417/16088) | 8.1% (1216/15023) | 1.097 (0.045) | -10.6% (0.1479) |
| BMI>25 | ↓ | Paracetamol | General | - | 1.221 (0.056) | -13.9% (0.0860) | 9.6% (3723/38633) | 8.1% (1216/15023) | 1.211 (0.042) | -10.7% (0.0851) |
| BMI>30 | ↓ | 3-((l-menthyl )oxy ) propane-1,2-diol | General | - | 1.307 (0.131) | -6.1% (0.5549) | 8.4% (656/7822) | 6.8% (323/4744) | 1.253 (0.088) | -7.9% (0.2955) |
| | | | Joint replacement and common orthopeadic surgery | 18.40% | 1.579 (0.362) | -28.1% (0.3659) | 6.4% (107/1666) | 3.7% (24/651) | 1.793 (0.414) | 11.8% (0.7388) |

© 2022 Liu K et al. *JAMA Network Open.*

| Target factor | Effect change in meta | Moderator | Controlled population | % of sample account for | Subgroup model | | Subgroup analysis | | | |
|---|---|---|---|---|---|---|---|---|---|---|
| | | | | | OR (se) | OR change (p-value) | Risk in exposed group | Risk in unexposed group | OR (se) | OR change (p-value) |
| BMI 25-30 | ↓ | 3-((l-menthyl )oxy ) propane-1,2-diol | General | - | 1.087 (0.114) | -9.3% (0.3816) | 7.3% (408/5596) | 6.8% (323/4744) | 1.076 (0.083) | -6.5% (0.4343) |
| | | | Joint replacement and common orthopeadic surgery | 15.80% | 0.860 (0.241) | -43.6% (0.1128) | 3.4% (34/987) | 3.7% (24/651) | 0.932 (0.253) | -26.7% (0.4140) |
| BMI>25 | ↓ | 3-((l-menthyl )oxy ) propane-1,2-diol | General | - | 1.180 (0.095) | -7.0% (0.4132) | 7.9% (1064/13418) | 6.8% (323/4744) | 1.179 (0.078) | -7.3% (0.3061) |
| | | | Joint replacement and common orthopeadic surgery | 18.20% | 1.315 (0.397) | -29.5% (0.3253) | 5.3% (141/2653) | 3.7% (24/651) | 1.466 (0.330) | -0.9% (0.9789) |
| **BMI>30** | ↓ | **Benzodiazepine** | **†General** | **-** | **1.377 (0.072)** | **-0.8% (0.9438)** | **10.6% (2033/19158)** | **8.6% (1089/12636)** | **1.259 (0.050)** | **-13.0% (0.0357)** |
| | | | Joint replacement and common orthopeadic surgery | 12.00% | 1.916 (0.550) | 42.7% (0.3415) | 6.2% (175/2834) | 3.4% (33/982) | 1.893 (0.366) | 8.0% (0.8478) |
| BMI 25-30 | ↓ | Benzodiazepine | General | - | 1.143 (0.055) | -9.3% (0.3223) | 9.2% (1283/13913) | 8.6% (1089/12636) | 1.077 (0.046) | -11.5% (0.0914) |
| | | | Joint replacement and common orthopeadic surgery | 9.90% | 1.320 (0.330) | 38.2% (0.3284) | 3.9% (64/1643) | 3.4% (33/982) | 1.166 (0.254) | -5.6% (0.8964) |
| **BMI>25** | ↓ | **Benzodiazepine** | **†General** | **-** | **1.237 (0.049)** | **-4.1% (0.5780)** | **10.0% (3316/33071)** | **8.6% (1089/12636)** | **1.182 (0.043)** | **-12.3% (0.0319)** |
| | | | Joint replacement and common orthopeadic surgery | 11.90% | 1.612 (0.374) | 27.0% (0.4662) | 5.3% (239/4477) | 3.4% (33/982) | 1.622 (0.307) | 6.3% (0.8729) |
| Pulse>100 | ↑ | Fentanyl | General | - | 1.420 (0.088) | 10.8% (0.4532) | 12.3% (708/5740) | 8.4% (2592/30704) | 1.526 (0.069) | 8.8% (0.3572) |
| Pulse 80-100 | ↑ | Fentanyl | General | - | 1.116 (0.041) | 1.1% (0.8667) | 9.9% (2123/21549) | 8.4% (2592/30704) | 1.185 (0.036) | 0.9% (0.8865) |
| Pulse>80 or <50 | ↑ | Fentanyl | General | - | 1.184 (0.038) | 0.4% (0.9385) | 10.5% (2895/27587) | 8.4% (2592/30704) | 1.272 (0.036) | 3.5% (0.5458) |
| Pulse>100 | ↑ | Potassium chloride | General | - | 1.372 (0.084) | -4.7% (0.7650) | 12.2% (776/6375) | 8.7% (2790/32080) | 1.455 (0.063) | -12.7% (0.1936) |
| | | | Joint replacement and common orthopeadic surgery | | 1.199 (0.318) | 43.6% (0.2694) | 5.5% (32/580) | 5.8% (176/3036) | 0.949 (0.188) | 123.7% (0.4741) |
| Pulse 80-100 | ↑ | Potassium chloride | General | - | 1.126 (0.038) | 13.7% (0.1700) | 10.1% (2353/23380) | 8.7% (2790/32080) | 1.175 (0.035) | -1.3% (0.8509) |

© 2022 Liu K et al. *JAMA Network Open.*

| Target factor | Effect change in meta | Moderator | Controlled population | % of sample account for | Subgroup model | | Subgroup analysis | | | |
|---|---|---|---|---|---|---|---|---|---|---|
| | | | | | **OR (se)** | **OR change (p-value)** | **Risk in exposed group** | **Risk in unexposed group** | **OR (se)** | **OR change (p-value)** |
| | | | Joint replacement and common orthopeadic surgery | 10.20% | 0.859 (0.137) | 14.0% (0.6069) | 4.7% (124/2611) | 5.8% (176/3036) | 0.810 (0.098) | 27.3% (0.6451) |
| Pulse>80 or <50 | ↑ | Potassium chloride | General | - | 1.191 (0.041) | 8.1% (0.3581) | 10.6% (3197/30062) | 8.7% (2790/32080) | 1.249 (0.034) | -2.9% (0.6535) |
| | | | Joint replacement and common orthopeadic surgery | 10.10% | 0.897 (0.134) | 12.8% (0.6277) | 4.9% (158/3213) | 5.8% (176/3036) | 0.840 (0.095) | 25.5% (0.6467) |
| Pulse>100 | ↑ | Benzimidazole | General | - | 1.309 (0.091) | -14.8% (0.1340) | 12.3% (520/4235) | 8.9% (1767/19838) | 1.431 (0.076) | -8.1% (0.2858) |
| Pulse 80-100 | ↑ | Benzimidazole | General | - | 1.155 (0.053) | 7.7% (0.2791) | 10.6% (1559/14695) | 8.9% (1767/19838) | 1.214 (0.044) | 6.7% (0.2297) |
| Pulse>80 or <50 | ↑ | Benzimidazole | General | - | 1.191 (0.045) | 2.0% (0.7182) | 11.1% (2127/19136) | 8.9% (1767/19838) | 1.279 (0.043) | 4.1% (0.4167) |
| **Vancomycin** | ↑ | **Tazobactam** | ***General** | **-** | **1.968 (0.154)** | **53.8% (0.0000)** | **21.8% (1426/6542)** | **11.9% (850/7119)** | **2.056 (0.097)** | **56.9% (0.0000)** |
| | | | ***Systemic infection** | **19.20%** | **2.776 (0.580)** | **119.3% (0.0185)** | **24.4% (413/1692)** | **13.7% (128/931)** | **2.026 (0.224)** | **78.5% (0.0018)** |
| | | | **Other common infection** | **13.40%** | **2.555 (0.697)** | **77.9% (0.1168)** | **17.8% (163/917)** | **11.7% (107/916)** | **1.634 (0.220)** | **27.1% (0.2326)** |
| | | | ***Infection** | **32.60%** | **2.354 (0.383)** | **102.9% (0.0049)** | **22.1% (576/2609)** | **12.7% (235/1847)** | **1.943 (0.164)** | **49.0% (0.0029)** |
| **Vancomycin** | ↑ | **Lactate** | **#General** | **-** | **1.792 (0.108)** | **19.8% (0.0183)** | **11.5% (1012/8808)** | **7.4% (1637/22148)** | **1.626 (0.069)** | **-30.3% (0.0000)** |
| | | | Joint replacement and common orthopeadic surgery | 19.80% | 1.362 (0.270) | 7.8% (0.7895) | 5.4% (165/3044) | 4.8% (148/3077) | 1.134 (0.132) | 30.6% (0.5118) |
| **Vancomycin** | ↑ | **Glucose** | ***General** | **-** | **1.590 (0.070)** | **33.0% (0.0195)** | **16.9% (2246/13267)** | **9.9% (2527/25525)** | **1.855 (0.058)** | **175.3% (0.0000)** |
| **Vancomycin** | ↑ | **Budipine hcl** | **#General** | **-** | **1.831 (0.133)** | **25.1% (0.0132)** | **12.4% (901/7245)** | **8.5% (1617/19067)** | **1.533 (0.068)** | **-31.1% (0.0000)** |
| | | | Joint replacement and common orthopeadic surgery | 17.60% | 1.435 (0.313) | 18.6% (0.5973) | 5.3% (132/2491) | 5.3% (112/2132) | 1.009 (0.133) | -10.1% (0.6558) |
| **Vancomycin** | ↑ | **Morphine** | General | - | 1.688 (0.110) | 16.9% (0.0699) | 14.2% (1149/8065) | 7.6% (1912/25061) | 2.011 (0.080) | 7.5% (0.1774) |
| | | | **†Joint replacement and common orthopeadic surgery** | **12.10%** | **1.414 (0.321)** | **5.0% (0.8605)** | **6.6% (116/1763)** | **5.4% (120/2236)** | **1.242 (0.167)** | **76.0% (0.0135)** |
| **Vancomycin** | ↓ | **Simvastatin** | General | - | 1.350 | -14.6% | 15.8% | 9.3% | 1.824 | -5.9% |

© 2022 Liu K et al. *JAMA Network Open.*

| Target factor | Effect change in meta | Moderator | Controlled population | % of sample account for | Subgroup model | | Subgroup analysis | | | |
|---|---|---|---|---|---|---|---|---|---|---|
| | | | | | OR (se) | OR change (p-value) | Risk in exposed group | Risk in unexposed group | OR (se) | OR change (p-value) |
| | | | | | (0.137) | (0.1424) | (427/2710) | (818/8797) | (0.117) | (0.3929) |
| | | | Cardiac Catheterization | 8.90% | 1.164 (0.322) | 78.2% (0.2016) | 12.2% (5/41) | 6.6% (65/988) | 1.972 (0.975) | 105.9% (0.2455) |
| | | | Major cardiac surgery | 8.30% | 1.510 (0.416) | 55.5% (0.2305) | 22.9% (112/489) | 28.3% (133/470) | 0.753 (0.112) | 20.2% (0.3548) |
| | | | **†Joint replacement and common orthopeadic surgery** | **9.00%** | **1.031 (0.293)** | **-33.8% (0.2378)** | **5.8% (29/501)** | **9.0% (48/534)** | **0.622 (0.152)** | **-45.4% (0.0264)** |
| **Vancomycin** | ↓ | **Aminobutyrate** | ***General** | - | **1.295 (0.111)** | **-19.0% (0.0267)** | **15.7% (546/3467)** | **10.0% (1192/11947)** | **1.687 (0.094)** | **-15.5% (0.0080)** |
| | | | Cerebrovascular | 12.50% | 2.201 (0.746) | 18.0% (0.7242) | 9.6% (24/250) | 5.9% (98/1673) | 1.707 (0.407) | -58.0% (0.0258) |
| **Vancomycin** | ↓ | **Magnesium hydroxide** | ***General** | - | **1.401 (0.073)** | **-28.4% (0.0001)** | **12.2% (1333/10921)** | **7.5% (2322/30950)** | **1.714 (0.062)** | **-29.2% (0.0000)** |
| | | | Joint replacement and common orthopeadic surgery | 14.10% | 1.278 (0.244) | -10.6% (0.7548) | 5.3% (158/3000) | 5.0% (143/2885) | 1.066 (0.126) | -34.1% (0.2102) |
| **Vancomycin** | ↓ | **Benzoic acid** | **#General** | - | **1.366 (0.086)** | **-21.7% (0.0044)** | **19.8% (1163/5864)** | **12.8% (1912/14962)** | **1.689 (0.069)** | **-9.5% (0.0652)** |
| | | | Infection | 13.00% | 1.649 (0.381) | -26.4% (0.2474) | 21.2% (305/1439) | 12.9% (162/1260) | 1.823 (0.193) | -6.9% (0.5844) |
| | | | Heart fail, cardiac catheterization exc ischemic heart disease, cardiac defibrillator & heart assist | 8.80% | 1.407 (0.411) | 9.8% (0.8343) | 20.1% (51/254) | 15.5% (246/1582) | 1.364 (0.234) | -43.7% (0.2408) |
| | | | Major cardiac surgery | 8.40% | 1.149 (0.280) | 33.0% (0.4082) | 20.7% (195/941) | 20.4% (165/810) | 1.022 (0.121) | 171.9% (0.0001) |

© 2022 Liu K et al. *JAMA Network Open.*

**eTable 18.** Top 50 Features in Patient Similarity Calculation

These features affect the similar sample matching. To calculate importance of features in similar sample matching, we multiply weight of features in similarity measure by their standard deviation. According to the AUROC change of PMTL, we found sample matching were mainly affected by 50 features and among them, 46 features were modifiable.

| **Feature** | **Weight *Std** | **Weight** | **Rank** | **Feature** | **Weight *Std** | **Weight** | **Rank** |
|---|---|---|---|---|---|---|---|
| **Demo** | | | | **Drug-Antiinfectives** | | | |
| Age | 7.94 | 2.43 | 1 | Tazobactam | 2.25 | 2.77 | 8 |
| Female | 1.57 | 1.48 | 16 | Vancomycin | 1.98 | 2.26 | 11 |
| Male | 1.52 | 1.44 | 18 | Levofloxacin | 1.58 | 1.71 | 15 |
| White | 1.27 | 1.40 | 28 | Lactate | 1.17 | 1.12 | 36 |
| **Vital** | | | | Imidazole | 1.09 | 1.24 | 44 |
| Pulse | 6.00 | 2.60 | 2 | **Drug-Antithrombotic** | | | |
| Temp | 4.50 | 1.85 | 3 | Heparin,porcine | 1.50 | 1.48 | 20 |
| Bmi | 4.30 | 1.75 | 4 | Enoxaparin | 1.25 | 1.21 | 29 |
| Systolic | 3.71 | 2.13 | 5 | **Drug-Analgesics** | | | |
| Diastolic | 1.51 | 1.04 | 19 | Oxycodone | 1.40 | 1.34 | 21 |
| **Label** | | | | Morphine | 1.32 | 1.25 | 25 |
| Troponin I | 2.38 | 1.92 | 6 | Paracetamol | 1.04 | 1.15 | 48 |
| Albumin | 2.26 | 1.46 | 7 | **Drug-Psycholeptics** | | | |
| WBC | 2.10 | 2.14 | 10 | Benzodiazepine | 1.13 | 1.11 | 39 |
| AST | 1.74 | 1.43 | 12 | Phenothiazine | 1.11 | 1.07 | 41 |
| Calcium | 1.54 | 1.53 | 17 | **Drug-Irrigating solutions/ Nutrients** | | | |
| Platelets | 1.33 | 1.42 | 24 | Glucose | 1.62 | 1.52 | 14 |
| Glucose | 1.15 | 1.49 | 37 | Hexanetriol | 1.24 | 1.18 | 30 |
| BUN | 1.12 | 1.20 | 40 | Calcium chloride | 1.22 | 1.16 | 32 |
| Total Bilirubin | 1.03 | 0.98 | 49 | **Drug-Other** | | | |
| **Drug-Alimentary Tract** | | | | Benzoic acid | 2.16 | 2.29 | 9 |
| Magnesium sulfate | 1.36 | 1.31 | 23 | Insulin,aspart, human/rdna | 1.73 | 1.77 | 13 |
| Sennosides | 1.23 | 1.16 | 31 | Benzimidazole | 1.39 | 1.30 | 22 |
| Magnesium hydroxide | 1.19 | 1.12 | 35 | Docosapentaenoic acid | 1.31 | 1.25 | 26 |
| Bisacodyl | 1.10 | 1.05 | 42 | Budipine hcl | 1.20 | 1.19 | 33 |
| Polyethylene glycol 3350 | 1.10 | 1.09 | 43 | Diphenhydramine | 1.19 | 1.13 | 34 |
| | | | | Aminobutyrate | 1.05 | 1.24 | 47 |
| Famotidine | 1.09 | 1.07 | 45 | Pyrrolidine naphthalenedisulfonate | 1.00 | 1.00 | 50 |

© 2022 Liu K et al. *JAMA Network Open.*

**eReferences**


1. Matheny ME, Miller RA, Ikizler TA, et al. Development of inpatient risk stratification models of acute kidney injury for use in electronic health records. *Medical Decision Making*. 2010;30(6):639-650.
2. Pan SJ, Yang Q. A survey on transfer learning. *IEEE Transactions on knowledge and data engineering*. 2009;22(10):1345-1359.
3. Wettschereck D, Aha DW, Mohri T. A review and empirical evaluation of feature weighting methods for a class of lazy learning algorithms. *Artificial Intelligence Review*. 1997;11(1-5):273-314.
4. Yosinski J, Clune J, Bengio Y, Lipson H. How transferable are features in deep neural networks? 2014:3320-3328.
5. Moutafis P, Leng M, Kakadiaris IA. An overview and empirical comparison of distance metric learning methods. *IEEE transactions on cybernetics*. 2016;47(3):612-625.
6. DeLong ER, DeLong DM, Clarke-Pearson DL. Comparing the areas under two or more correlated receiver operating characteristic curves: a nonparametric approach. *Biometrics*. 1988:837-845.
7. Churpek MM, Carey KA, Edelson DP, et al. Internal and external validation of a machine learning risk score for acute kidney injury. *JAMA network open*. 2020;3(8):e2012892-e2012892.
8. Lei VJ, Luong T, Shan E, et al. Risk stratification for postoperative acute kidney injury in major noncardiac surgery using preoperative and intraoperative data. *JAMA network open*. 2019;2(12):e1916921-e1916921.
9. Agresti A. *An introduction to categorical data analysis (2nd edition)*. John Wiley & Sons; 2018:106-108.
10. Agresti A. *Categorical data analysis (2nd edition)*. John Wiley & Sons; 2003:70-71.
11. Liu K, Yuan B, Zhang X, et al. Characterizing the Temporal Changes in Association Between Modifiable Risk Factors and Acute Kidney Injury with Multi-view Analysis. *International Journal of Medical Informatics*. 2022:104785.
12. Anderson S, Eldadah B, Halter JB, et al. Acute kidney injury in older adults. *Journal of the American Society of Nephrology*. 2011;22(1):28-38.
13. Li Q, Zhao M, Zhou F. Hospital-acquired acute kidney injury in very elderly men: clinical characteristics and short-term outcomes. *Aging clinical and experimental research*. 2019:1-8.
14. Popplewell L, Forman S. Is there an upper age limit for bone marrow transplantation? *Bone marrow transplantation*. 2002;29(4):277-284.
15. Thongprayoon C, Cheungpasitporn W, Chewcharat A, Mao MA, Kashani KB. Serum ionised calcium and the risk of acute respiratory failure in hospitalised patients: A single-centre cohort study in the USA. *BMJ open*. 2020;10(3):e034325.
16. Klein GL. Burns: where has all the calcium (and vitamin D) gone? *Advances in nutrition*. 2011;2(6):457-462.
17. Klein GL. Burn-induced bone loss: importance, mechanisms, and management. *Journal of burns and wounds*. 2006;5


© 2022 Liu K et al. *JAMA Network Open.*

18. Yan Sd, Liu Xj, Peng Y, et al. Admission serum calcium levels improve the GRACE risk score prediction of hospital mortality in patients with acute coronary syndrome. *Clinical cardiology*. 2016;39(9):516-523.
19. Lu X, Wang Y, Meng H, et al. Association of admission serum calcium levels and in-hospital mortality in patients with acute ST-elevated myocardial infarction: an eight-year, single-center study in China. *PloS one*. 2014;9(6):e99895.
20. Gai P, Sun H, Sui L, Wang G. Hypocalcaemia after total knee arthroplasty and its clinical significance. *Anticancer research*. 2016;36(3):1309-1311.
21. Aderka D, Schwartz D, Dan M, Levo Y. Bacteremic hypocalcemia: A comparison between the calcium levels of bacteremic and nonbacteremic patients with infection. *Archives of internal medicine*. 1987;147(2):232-236.
22. Gerstein NS, Brierley JK, Windsor J, et al. Antifibrinolytic agents in cardiac and noncardiac surgery: a comprehensive overview and update. *Journal of cardiothoracic and vascular anesthesia*. 2017;31(6):2183-2205.
23. Lacko L, Wittke B, Zimmer G. Interaction of benzoic acid derivatives with the transport system of glucose in human erythrocytes. *Biochemical pharmacology*. 1981;30(12):1425-1431.
24. Gayathri M, Kannabiran K. Effect of 2-hydroxy-4-methoxy benzoic acid isolated from Hemidesmus indicus on erythrocyte membrane bound enzymes and antioxidant status in streptozotocin-induced diabetic rats. *Indian journal of pharmaceutical sciences*. 2012;74(5):474.
25. Mineo H, Ohdate T, Fukumura K, et al. Effects of benzoic acid and its analogues on insulin and glucagon secretion in sheep. *European journal of pharmacology*. 1995;280(2):149-154.
26. Jorge PF, Wieringa N, de Felice E, van der Horst IC, Lansink AO, Nijsten MW. The association of early combined lactate and glucose levels with subsequent renal and liver dysfunction and hospital mortality in critically ill patients. *Critical Care*. 2017;21(1):1-11.
27. Sotello D, Yang S, Nugent K. Glucose and lactate levels at admission as predictors of in-hospital mortality. *Cureus*. 2019;11(10)
28. Basu A, Veettil S, Dyer R, Peyser T, Basu R. Direct evidence of acetaminophen interference with subcutaneous glucose sensing in humans: a pilot study. *Diabetes technology & therapeutics*. 2016;18(S2):S2-43-S2-47.
29. Maahs DM, DeSalvo D, Pyle L, et al. Effect of acetaminophen on CGM glucose in an outpatient setting. *Diabetes Care*. 2015;38(10):e158-e159.
30. Zammit C, Liddicoat H, Moonsie I, Makker H. Obesity and respiratory diseases. *International journal of general medicine*. 2010;3:335.
31. Reagan JL, Ingham RR, Dalia S, et al. Association between obesity/overweight and leukemia: A meta-analysis of prospective cohort studies. American Society of Hematology; 2011.
32. Li S, Chen L, Jin W, et al. Influence of body mass index on incidence and prognosis of acute myeloid leukemia and acute promyelocytic leukemia: A meta-analysis. *Scientific reports*. 2017;7(1):1-10.
33. Castillo JJ, Reagan JL, Ingham RR, et al. Obesity but not overweight increases the incidence and mortality of leukemia in adults: a meta-analysis of prospective cohort studies. *Leukemia research*. 2012;36(7):868-875.
34. Castillo JJ, Mulkey F, Geyer S, et al. Relationship between obesity and clinical outcome in adults with acute myeloid leukemia: A pooled analysis from four CALGB (alliance) clinical trials. *American journal of hematology*. 2016;91(2):199-204.

© 2022 Liu K et al. *JAMA Network Open.*

35. Thomas D, Ikeda M, Deepika K, Medina C, Takacs P. Laparoscopic management of benign adnexal mass in obese women. *Journal of minimally invasive gynecology*. 2006;13(4):311-314.
36. Eltabbakh GH, Piver MS, Hempling RE, Recio FO. Laparoscopic surgery in obese women. *Obstetrics & Gynecology*. 1999;94(5):704-708.
37. Meng L, Mui E, Holubar MK, Deresinski SC. Comprehensive guidance for antibiotic dosing in obese adults. *Pharmacotherapy: The Journal of Human Pharmacology and Drug Therapy*. 2017;37(11):1415-1431.
38. Veillette JJ, Winans SA, Maskiewicz VK, Truong J, Jones RN, Forland SC. Pharmacokinetics and Pharmacodynamics of High-Dose Piperacillin–Tazobactam in Obese Patients. *European journal of drug metabolism and pharmacokinetics*. 2021;46(3):385-394.
39. Chung EK, Cheatham SC, Fleming MR, Healy DP, Shea KM, Kays MB. Population pharmacokinetics and pharmacodynamics of piperacillin and tazobactam administered by prolonged infusion in obese and nonobese patients. *The Journal of Clinical Pharmacology*. 2015;55(8):899-908.
40. Zakrison TL, Hille DA, Namias N. Effect of body mass index on treatment of complicated intra-abdominal infections in hospitalized adults: comparison of ertapenem with piperacillin-tazobactam. *Surgical infections*. 2012;13(1):38-42.
41. Fede G, Privitera G, Tomaselli T, Spadaro L, Purrello F. Cardiovascular dysfunction in patients with liver cirrhosis. *Annals of Gastroenterology: Quarterly Publication of the Hellenic Society of Gastroenterology*. 2015;28(1):31.
42. Ritter MA, Rohde A, Heuschmann PU, et al. Heart rate monitoring on the stroke unit. What does heart beat tell about prognosis? An observational study. *BMC neurology*. 2011;11(1):1-8.
43. Lee K-J, Kim BJ, Han M-K, et al. Effect of Heart Rate on Stroke Recurrence and Mortality in Acute Ischemic Stroke With Atrial Fibrillation. *Stroke*. 2020;51(1):162-169.
44. Rapenne T, Moreau D, Lenfant F, et al. Could heart rate variability predict outcome in patients with severe head injury?: A pilot study. *Journal of neurosurgical anesthesiology*. 2001;13(3):260-268.
45. Winchell RJ, Hoyt DB. Analysis of heart-rate variability: a noninvasive predictor of death and poor outcome in patients with severe head injury. *Journal of Trauma and Acute Care Surgery*. 1997;43(6):927-933.
46. Luther M, Caffrey A, Dosa D, Lodise TP, Laplante K. Vancomycin plus piperacillin/tazobactam and acute kidney injury in adults: a systematic review and meta-analysis. Oxford University Press; 2016:
47. Thangamani S, Mohammad H, Abushahba MF, et al. Exploring simvastatin, an antihyperlipidemic drug, as a potential topical antibacterial agent. *Scientific reports*. 2015;5(1):1-13.
48. Graziano TS, Cuzzullin MC, Franco GC, et al. Statins and antimicrobial effects: simvastatin as a potential drug against Staphylococcus aureus biofilm. *PloS one*. 2015;10(5):e0128098.
49. Wu L, Hu Y, Liu X, et al. Feature Ranking in Predictive Models for Hospital-Acquired Acute Kidney Injury. *Scientific reports*. 2018;8(1):17298.
50. Xu X, Ling Q, Wei Q, et al. An effective model for predicting acute kidney injury after liver transplantation. *Hepatobiliary & pancreatic diseases international: HBPD INT*. 2010;9(3):259-263.

© 2022 Liu K et al. *JAMA Network Open.*

51. Lee H-C, Yoon S, Yang S-M, et al. Prediction of acute kidney injury after liver transplantation: Machine learning approaches vs. logistic regression model. *Journal of clinical medicine*. 2018;7(11):428.
52. Park MH, Shim HS, Kim WH, et al. Clinical risk scoring models for prediction of acute kidney injury after living donor liver transplantation: a retrospective observational study. *PLoS One*. 2015;10(8):e0136230.
53. Ma B, Allen DW, Graham MM, et al. Comparative Performance of Prediction Models for Contrast-Associated Acute Kidney Injury After Percutaneous Coronary Intervention. *Circulation-Cardiovascular Quality and Outcomes*. Nov 2019;12(11)e005854. doi:10.1161/circoutcomes.119.005854
54. Chen YL, Fu NK, Xu J, et al. A simple preprocedural score for risk of contrast‐induced acute kidney injury after percutaneous coronary intervention. *Catheterization and Cardiovascular Interventions*. 2014;83(1):E8-E16.
55. Inohara T, Kohsaka S, Abe T, et al. Development and validation of a pre-percutaneous coronary intervention risk model of contrast-induced acute kidney injury with an integer scoring system. *The American journal of cardiology*. 2015;115(12):1636-1642.
56. Inohara T, Kohsaka S, Miyata H, et al. Performance and validation of the US NCDR acute kidney injury prediction model in Japan. *Journal of the American College of Cardiology*. 2016;67(14):1715-1722.
57. Tsai TT, Patel UD, Chang TI, et al. Validated contemporary risk model of acute kidney injury in patients undergoing percutaneous coronary interventions: insights from the National Cardiovascular Data Registry Cath‐PCI Registry. *Journal of the American Heart Association*. 2014;3(6):e001380.
58. Huang C, Murugiah K, Mahajan S, et al. Enhancing the prediction of acute kidney injury risk after percutaneous coronary intervention using machine learning techniques: A retrospective cohort study. *PLoS medicine*. 2018;15(11):e1002703.
59. Huang C, Li S-X, Mahajan S, et al. Development and Validation of a Model for Predicting the Risk of Acute Kidney Injury Associated With Contrast Volume Levels During Percutaneous Coronary Intervention. *Jama Network Open*. Nov 2019;2(11)e1916021. doi:10.1001/jamanetworkopen.2019.16021
60. Hoang Van S, Nguyen Minh K, Tran Nguyen Phuong H, Mai Tri L, Luu Truc P, Chau Ngoc H. Comparison Between Two Risk Models for Predicting Contrast-Induced Acute Kidney Injury in Patients with Reduced Glomerular Filtration Rate Undergoing Percutaneous Coronary Intervention. *Journal of Complementary Medicine Research*. 2020 2020;11(3):30-39. doi:10.5455/jcmr.2020.11.03.05
61. Blanco A, Rahim F, Nguyen M, et al. Performance of a pre-procedural Mehran score to predict acute kidney injury after percutaneous coronary intervention. *Nephrology (Carlton, Vic)*. Jan 2021;26(1):23-29. doi:10.1111/nep.13769
62. Araujo GN, Junior FP, Fuhr B, et al. Simplifying contrast-induced acute kidney injury prediction after primary percutaneous coronary intervention: the age, creatinine and ejection fraction score. *Cardiovascular intervention and therapeutics*. 2018;33(3):224-231.
63. Liu YH, Liu Y, Tan N, et al. Predictive value of GRACE risk scores for contrast-induced acute kidney injury in patients with ST-segment elevation myocardial infarction before undergoing primary percutaneous coronary intervention. *International urology and nephrology*. 2014;46(2):417-426.

© 2022 Liu K et al. *JAMA Network Open.*

64. Park HS, Kim CJ, Yi J-E, et al. Contrast volume/raw eGFR ratio for predicting contrast-induced acute kidney injury in patients undergoing percutaneous coronary intervention for myocardial infarction. *Cardiorenal medicine*. 2015;5(1):61-68.
65. Zambetti BR, Thomas F, Hwang I, et al. A web-based tool to predict acute kidney injury in patients with ST-elevation myocardial infarction: Development, internal validation and comparison. *PloS one*. 2017;12(7):e0181658.
66. Sun L, Zhu W, Chen X, et al. Machine Learning to Predict Contrast-Induced Acute Kidney Injury in Patients With Acute Myocardial Infarction. *Frontiers in Medicine*. Nov 13 2020;7592007. doi:10.3389/fmed.2020.592007
67. Xu F-b, Cheng H, Yue T, Ye N, Zhang H-j, Chen Y-p. Derivation and validation of a prediction score for acute kidney injury secondary to acute myocardial infarction in Chinese patients. *Bmc Nephrology*. May 30 2019;20195. doi:10.1186/s12882-019-1379-x
68. Lin H, Hou J, Tang H, et al. A novel nomogram to predict perioperative acute kidney injury following isolated coronary artery bypass grafting surgery with impaired left ventricular ejection fraction. *Bmc Cardiovascular Disorders*. Dec 10 2020;20(1)517. doi:10.1186/s12872-020-01799-1
69. Chen S-W, Chang C-H, Fan P-C, et al. Comparison of contemporary preoperative risk models at predicting acute kidney injury after isolated coronary artery bypass grafting: a retrospective cohort study. *BMJ open*. 2016;6(6):e010176.
70. Gursoy M, Hokenek AF, Duygu E, Atay M, Yavuz A. Clinical SYNTAX score can predict acute kidney injury following on-pump but not off-pump coronary artery bypass surgery. *Cardiorenal medicine*. 2015;5(4):297-305.
71. Palomba H, De Castro I, Neto A, Lage S, Yu L. Acute kidney injury prediction following elective cardiac surgery: AKICS Score. *Kidney international*. 2007;72(5):624-631.
72. Ho J, Reslerova M, Gali B, et al. Serum creatinine measurement immediately after cardiac surgery and prediction of acute kidney injury. *American Journal of Kidney Diseases*. 2012;59(2):196-201.
73. Jiang W, Xu J, Shen B, Wang C, Teng J, Ding X. Validation of four prediction scores for cardiac surgery-associated acute kidney injury in Chinese patients. *Brazilian journal of cardiovascular surgery*. 2017;32(6):481-486.
74. Jiang W, Teng J, Xu J, et al. Dynamic predictive scores for cardiac surgery–associated acute kidney injury. *Journal of the American Heart Association*. 2016;5(8):e003754.
75. Nah CW, Ti LK, Liu W, Ng RRG, Shen L, Chew STH. A clinical score to predict acute kidney injury after cardiac surgery in a Southeast-Asian population. *Interactive cardiovascular and thoracic surgery*. 2016;23(5):757-761.
76. Tseng P-Y, Chen Y-T, Wang C-H, et al. Prediction of the development of acute kidney injury following cardiac surgery by machine learning. *Critical Care*. Jul 31 2020;24(1)478. doi:10.1186/s13054-020-03179-9
77. Hu P, Chen Y, Wu Y, et al. Development and validation of a model for predicting acute kidney injury after cardiac surgery in patients of advanced age. *Journal of Cardiac Surgery*. Mar 2021;36(3):806-814. doi:10.1111/jocs.15249
78. Coulson T, Bailey M, Pilcher D, et al. Predicting Acute Kidney Injury After Cardiac Surgery Using a Simpler Model. *J Cardiothorac Vasc Anesth*. Mar 2021;35(3):866-873. doi:10.1053/j.jvca.2020.06.072

© 2022 Liu K et al. *JAMA Network Open.*

79. Hougardy J-M, Revercez P, Pourcelet A, et al. Chronic kidney disease as major determinant of the renal risk related to on-pump cardiac surgery: a single-center cohort study. *Acta Chirurgica Belgica*. 2016;116(4):217-224.
80. Crosina J, Lerner J, Ho J, et al. Improving the prediction of cardiac surgery–associated acute kidney injury. *Kidney international reports*. 2017;2(2):172-179.
81. Wong B, Onge JS, Korkola S, Prasad B. Validating a scoring tool to predict acute kidney injury (AKI) following cardiac surgery. *Canadian journal of kidney health and disease*. 2015;2(1):3.
82. Guan C, Li C, Xu L, et al. Risk factors of cardiac surgery-associated acute kidney injury: development and validation of a perioperative predictive nomogram. *Journal of Nephrology*. Dec 2019;32(6):937-945. doi:10.1007/s40620-019-00624-z
83. Li Y, Xu J, Wang Y, et al. A novel machine learning algorithm, Bayesian networks model, to predict the high-risk patients with cardiac surgery-associated acute kidney injury. *Clinical Cardiology*. Jul 2020;43(7):752-761. doi:10.1002/clc.23377
84. Kristovic D, Horvatic I, Husedzinovic I, et al. Cardiac surgery-associated acute kidney injury: risk factors analysis and comparison of prediction models. *Interactive cardiovascular and thoracic surgery*. 2015;21(3):366-373.
85. Kiers HD, van den Boogaard M, Schoenmakers MC, et al. Comparison and clinical suitability of eight prediction models for cardiac surgery-related acute kidney injury. *Nephrology Dialysis Transplantation*. 2012;28(2):345-351.
86. Ranucci M, Aloisio T, Cazzaniga A, Di Dedda U, Gallazzi C, Pistuddi V. Validation of renal-risk models for the prediction of non-renal replacement therapy cardiac surgery-associated acute kidney injury. *International journal of cardiology*. 2018;272:49-53.
87. Enger TB, Pleym H, Stenseth R, Greiff G, Wahba A, Videm V. A preoperative multimarker approach to evaluate acute kidney injury after cardiac surgery. *Journal of cardiothoracic and vascular anesthesia*. 2017;31(3):837-846.
88. Berg KS, Stenseth R, Wahba A, Pleym H, Videm V. How can we best predict acute kidney injury following cardiac surgery?: a prospective observational study. *European Journal of Anaesthesiology (EJA)*. 2013;30(11):704-712.
89. Jorge-Monjas P, Bustamante-Munguira J, Lorenzo M, et al. Predicting cardiac surgery–associated acute kidney injury: The CRATE score. *Journal of critical care*. 2016;31(1):130-138.
90. Birnie K, Verheyden V, Pagano D, et al. Predictive models for kidney disease: improving global outcomes (KDIGO) defined acute kidney injury in UK cardiac surgery. *Critical Care*. 2014;18(6):606.
91. Ko S, Jo C, Chang CB, et al. A web-based machine-learning algorithm predicting postoperative acute kidney injury after total knee arthroplasty. *Knee Surgery Sports Traumatology Arthroscopy*. 2020;doi:10.1007/s00167-020-06258-0
92. Bell S, Dekker FW, Vadiveloo T, et al. Risk of postoperative acute kidney injury in patients undergoing orthopaedic surgery—development and validation of a risk score and effect of acute kidney injury on survival: observational cohort study. *Bmj*. 2015;351
93. Patel M, Robinson C, Smith R, Collaborative ST. Prognostic model to predict postoperative acute kidney injury in patients undergoing major gastrointestinal surgery based on a national prospective observational cohort study (vol 2, pg 400, 2018). *Bjs Open*. Aug 2019;3(4):549-549. doi:10.1002/bjs5.50199

© 2022 Liu K et al. *JAMA Network Open.*

94. Li Y, Chen X, Shen Z, et al. Prediction models for acute kidney injury in patients with gastrointestinal cancers: a real-world study based on Bayesian networks. *Renal Failure*. Jan 1 2020;42(1):869-876. doi:10.1080/0886022x.2020.1810068
95. Li Y, Chen X, Wang Y, Hu J, Shen Z, Ding X. Application of group LASSO regression based Bayesian networks in risk factors exploration and disease prediction for acute kidney injury in hospitalized patients with hematologic malignancies. *Bmc Nephrology*. May 5 2020;21(1)162. doi:10.1186/s12882-020-01786-w
96. Motwani SS, McMahon GM, Humphreys BD, Partridge AH, Waikar SS, Curhan GC. Development and Validation of a Risk Prediction Model for Acute Kidney Injury After the First Course of Cisplatin. *Journal of clinical oncology: official journal of the American Society of Clinical Oncology*. 2018;36(7):682-688.
97. Xu N, Zhang Q, Wu G, Lv D, Zheng Y. Derivation and Validation of a Risk Prediction Model for Vancomycin-Associated Acute Kidney Injury in Chinese Population. *Therapeutics and Clinical Risk Management*. 2020 2020;16:539-550. doi:10.2147/tcrm.S253587
98. Pan C, Wen A, Li X, et al. Development and Validation of a Risk Prediction Model of Vancomycin - Associated Nephrotoxicity in Elderly Patients: A Pilot Study. *Clinical and translational science*. 2020;13(3):491-497.
99. Xie Y, Zhang Y, Tian R, et al. A prediction model of sepsis-associated acute kidney injury based on antithrombin III. *Clinical and Experimental Medicine*. Feb 2021;21(1):89-100. doi:10.1007/s10238-020-00656-x
100. Zhou J, Bai Y, Wang X, et al. A simple risk score for prediction of sepsis associated-acute kidney injury in critically ill patients. *Journal of Nephrology*. Dec 2019;32(6):947-956. doi:10.1007/s40620-019-00625-y
101. Deng F, Peng M, Li J, Chen Y, Zhang B, Zhao S. Nomogram to predict the risk of septic acute kidney injury in the first 24 h of admission: an analysis of intensive care unit data. *Renal Failure*. Jan 1 2020;42(1):428-436. doi:10.1080/0886022x.2020.1761832
102. Fan C, Ding X, Song Y. A new prediction model for acute kidney injury in patients with sepsis. *Annals of palliative medicine*. Feb 2021;10(2):1772-1778. doi:10.21037/apm-20-1117
103. Kate RJ, Perez RM, Mazumdar D, Pasupathy KS, Nilakantan V. Prediction and detection models for acute kidney injury in hospitalized older adults. *BMC medical informatics and decision making*. 2016;16(1):39.
104. Kate RJ, Pearce N, Mazumdar D, Nilakantan V. A continual prediction model for inpatient acute kidney injury. *Computers in Biology and Medicine*. Jan 2020;116103580. doi:10.1016/j.compbiomed.2019.103580
